# Genome-wide analysis identifies a novel *LINC-PINT* splice variant associated with vascular amyloid pathology in Alzheimer’s disease

**DOI:** 10.1186/s40478-021-01199-2

**Published:** 2021-05-21

**Authors:** Joseph S. Reddy, Mariet Allen, Charlotte C. G. Ho, Stephanie R. Oatman, Özkan İş, Zachary S. Quicksall, Xue Wang, Jiangli Jin, Tulsi A. Patel, Troy P. Carnwath, Thuy T. Nguyen, Kimberly G. Malphrus, Sarah J. Lincoln, Minerva M. Carrasquillo, Julia E. Crook, Takahisa Kanekiyo, Melissa E. Murray, Guojun Bu, Dennis W. Dickson, Nilüfer Ertekin-Taner

**Affiliations:** 1 Department of Quantitative Health Sciences, Mayo Clinic, Jacksonville, FL 32224 USA; 2 Department of Neuroscience, Mayo Clinic, Jacksonville, FL 32224 USA; 3 Department of Neurology, Mayo Clinic, Jacksonville, FL 32224 USA

**Keywords:** Genome-wide association study, Cerebral amyloid angiopathy, Alzheimer’s disease, CAA, AD, LINC-PINT, GWAS, Splicing

## Abstract

Cerebral amyloid angiopathy (CAA) contributes to accelerated cognitive decline in Alzheimer’s disease (AD) dementia and is a common finding at autopsy. The *APOE*ε4 allele and male sex have previously been reported to associate with increased CAA in AD. To inform biomarker and therapeutic target discovery, we aimed to identify additional genetic risk factors and biological pathways involved in this vascular component of AD etiology. We present a genome-wide association study of CAA pathology in AD cases and report sex- and *APOE*-stratified assessment of this phenotype. Genome-wide genotypes were collected from 853 neuropathology-confirmed AD cases scored for CAA across five brain regions, and imputed to the Haplotype Reference Consortium panel. Key variables and genome-wide genotypes were tested for association with CAA in all individuals and in sex and *APOE*ε4 stratified subsets. Pathway enrichment was run for each of the genetic analyses. Implicated loci were further investigated for functional consequences using brain transcriptome data from 1,186 samples representing seven brain regions profiled as part of the AMP-AD consortium. We confirmed association of male sex, AD neuropathology and *APOE*ε4 with increased CAA, and identified a novel locus, *LINC-PINT,* associated with lower CAA amongst *APO*Eε4-negative individuals (rs10234094-C, beta = −3.70 [95% CI −0.49—−0.24]; p = 1.63E-08). Transcriptome profiling revealed higher *LINC-PINT* expression levels in AD cases, and association of rs10234094-C with altered *LINC-PINT* splicing. Pathway analysis indicates variation in genes involved in neuronal health and function are linked to CAA in AD patients. Further studies in additional and diverse cohorts are needed to assess broader translation of our findings.

**Supplementary Information:**

The online version contains supplementary material available at 10.1186/s40478-021-01199-2.

## Introduction

Alzheimer’s disease (AD), the most common form of dementia affecting the elderly, is definitively diagnosed at autopsy by the presence of both extracellular amyloid plaques (NP) and intracellular neurofibrillary tangles (NFT). Amyloid beta peptide (Aβ), the primary constituent of amyloid plaques, can also be found deposited in the brain cerebrovasculature, referred to as cerebral amyloid angiopathy (CAA). The majority of AD cases exhibit some degree of CAA, which impairs blood vessel integrity and leads to more rapid cognitive decline [10, 18]. AD neuropathologic change (ADNC), male sex and the *APOE*ε4 AD risk allele are associated with increased CAA at autopsy [12, 44]. Identifying additional genetic risk factors for CAA, in AD, can nominate genes and pathways underlying this aspect of the disease, providing key insights for biomarker discovery and development of targeted therapies.

Genetic association studies have implicated a role for some known AD risk loci in CAA [8, 29, 59]. A genome-wide association study (GWAS) identified significant association at the *APOE* locus [7], and pleiotropy analysis with ADNC (NP and NFT) nominated novel loci [14]. This prior work focused on combined assessment of both AD cases and controls, and a dichotomous or ordinal CAA phenotype. Given that CAA is associated with ADNC, it is feasible that the risk profile for CAA in AD cases may differ from that in controls; however, with the exception of *APOE*ε4 [44], the impact of genetic variants on CAA in AD specifically is currently unknown. Furthermore, GWAS for Alzheimer’s disease and AD neuropathology have identified sex and genotype-specific genetic risk factors [17, 22, 49]. We hypothesize that similar genetic analysis for CAA in the context of the major risk factors of sex and *APOE*ε4 may likewise lead to novel insights. Moreover, enrichment strategies leveraging the full spectrum of genome-wide association results can identify biological pathways that may play a role in disease regardless of whether individual variants achieve genome-wide significance (GWS); however these approaches have not yet been reported for CAA GWAS. In summary, the genetic architecture underlying risk for CAA in AD cases, and in the context of sex and *APOE*ε4 genotype, is yet to be established.

In this study we aimed to capture the genetic landscape of CAA susceptibility in neuropathologically defined AD cases, characterize sex and *APOE*ε4 context-specific genetic associations, and identify biological pathways involved in the disease. Importantly we focused on AD cases only and leveraged a continuous CAA phenotype. The functional significance of implicated variants was explored using brain transcriptomic data.

## Materials and methods

### Post-mortem series

The Mayo Clinic Brain Bank was queried to identify participants with available tissue samples that met neuropathological criteria for Alzheimer’s disease (NINCDS-ADRDA [31]), with a Braak stage ≥ four, scored for CAA pathology, and an age at death of greater than 55 years. The study size was maximized to include all available samples that met these criteria (MC-CAA dataset). To assess key findings in the absence of significant ADNC, the Mayo Clinic Brain Bank was also queried to identify participants that were scored for CAA but did not meet criteria for a diagnosis of AD (non-AD dataset). Due to availability, only individuals recorded as North American Caucasian were included. This study was approved by the appropriate Mayo Clinic Institutional Review Board.

### Neuropathology

CAA severity was scored using Thioflavin-S staining across five brain regions (inferior parietal cortex, middle frontal cortex, motor cortex, superior temporal cortex and visual cortex). Semi-quantitative scores were assigned for each of the regions defined as; zero = no amyloid positive vessels; 0.5 = scattered amyloid deposition only in leptomeninges; one = scattered amyloid deposition in both leptomeningeal and cortical vessels; two = strong circumferential amyloid deposition in multiple cortical and leptomeningeal vessels; three = widespread strong amyloid deposition in leptomeningeal and cortical vessels; four = same as score three plus extravasation of amyloid deposition accompanied by dyshoric amyloid. Notably only eleven individuals had a score of four in any one brain region. Scores were averaged and square root transformed (sqrtCAA) in order to meet the assumptions of parametric statistical tests. Thal phase [52] and Braak stage [11] were likewise collected using established approaches, as previously described [34, 35]. To reduce the number of variables, the distribution of CAA scores across Thal phases and Braak stages were evaluated and categories combined when not variable (Table S1). Furthermore, Braak stage was provided with intermediate levels and redefined as follows: zero (0 &0.5), one (1 &1.5), two (2 & 2.5), three (3 & 3.5), four (4 & 4.5), five (5 & 5.5) or six (6).

### MC-CAA genetic data

Genomic DNA was isolated from brain tissue of 853 AD cases using the AutoGen245T instrument (AutoGen) according to manufacturer’s protocols, incubated with two µl (4 mg/ml) RNAseA solution (Qiagen) and stored at −80 degrees Celsius prior to transfer to the Mayo Clinic Genome Analysis Core (GAC) in Rochester MN, for genotyping. Genome-wide genotypes (GWG) were generated for study participants in two batches (Additional file 1: Table S1), batch A (N = 477) and batch B (N = 376), using the Infinium Omni2.5 Exome8 v1.3 (A) or v1.4 (B) array. Genotypes were exported to a comma-separated final report file using Illumina’s GenomeStudio software v1.9.4 and v2.0.3, respectively. Final report files were converted to PLINK (v1.9) [13, 38] formatted lgen, fam, and map files using in-house scripts. Following quality control (Additional file 1: Figures S1-S2) 32 samples were removed resulting in a total of 821 samples for analysis. Data was imputed to the haplotype reference consortium (HRC) panel [28] (Additional file 1: Figure S1); variants with in imputation R2 ≥ 0.7 and a MAF ≥ 2% were retained resulting in 1,282,922 genotyped variants and 5,441,346 imputed variants. PLINK [13] was used to generate minor allele frequency and Hardy–Weinberg p-value annotation for all reported variants [57]. Where applicable, imputed dosages were converted to hard calls with uncertainty > 0.1 set to missing.

### AMP-AD brain transcriptome datasets

Brain transcriptome datasets on the AD Knowledge Portal (Additional file 1: Table S2) were utilized for functional annotations. For gene and exon QTL analysis (statistical analysis) independently processed gene and exon counts, and accompanying whole-genome sequencing (WGS) genotypes from the Mayo RNASeq dataset [1, 2] were utilized. The Mayo RNAseq dataset comprises transcriptome measures from temporal cortex (TCX) and cerebellum (CER); RNA isolation, data collection, sequencing alignment, counting and QC has been described in detail elsewhere [1, 2]. Gene counts were normalized using conditional quantile normalization (CQN) [20], RPKM exon counts ((10^9 × exon counts) / (total mapped reads x length of exon)), mapped to ensembl GRCh37/hg19 assembly, were transformed by log2 (1 + RPKM). Whole-genome sequencing (WGS) data was collected from individuals who passed prior QC, and was likewise shared on the AD Knowledge Portal along with detailed methods (Additional file 1: Table S2). Independent processing and QC of WGS data is outlined in additional file 1: Figure S3. Genotypes were extracted from VCF files using PLINK [13]. Selected variants were annotated using online databases [9, 40].

For transcriptome profiling analysis, consensus reprocessed counts from the Mayo RNASeq and two additional brain transcriptome datasets; the Religious Orders Study and Rush Memory Aging Project (ROSMAP) [16, 33], and the Mount Sinai Brain Bank (MSBB) study [56] were collectively assessed (Additional file 1: Figure S4). To reduce between-study variability, the AMP-AD consortium reprocessed the raw format RNASeq data from these three studies through a consensus alignment, counting and quality control pipeline, as detailed on the AD Knowledge Portal (https://adknowledgeportal.synapse.org/, Synapse ID: syn17010685) and elsewhere [55]. Gene counts and metadata for all three studies were downloaded from the AD Knowledge Portal (Additional file 1: Table S2), and underwent subsequent quality control (Additional file 1: Table S3), and CQN normalization. Neuropathological information provided in the available metadata files was used to assign individuals as AD, control, or other (Additional file 1: Table S3); only AD cases or controls were utilized for transcriptome profiling analysis.

### Statistical analysis

In the MC-CAA dataset, to assess for any genotyping batch effects, key variables were tested for their association with genotyping batch using the Wilcoxon rank sum test (sqrtCAA), linear regression (age at death), or chi-square test (Sex, *APOE*ε4 dose, Thal phase, and Braak stage) Additional file 1: Table S1 & Figures S5-S10. These same variables were tested for association with sqrtCAA in a multivariable linear regression model in the full dataset, and in subsets based on sex (Male-only, Female-only) or *APOE*ε4 genotype (“*APOE*ε4-neg” = *APOE*ε22, ε23, or ε33; “*APOE*ε4-pos” = *APOE*ε24, ε34, or ε44) where sex or *APOE* were excluded from the model respectively. These analyses were carried out using R statistical software version 3.6.2.

For the genome-wide association study (GWAS), variant dosages were tested for association with sqrtCAA using linear regression in PLINK (v2.00a2LM) [13, 38], as an additive model adjusting for age, sex, batch, the first three principal components (PCs) accounting for population substructure, Thal phase and Braak stage. Models were also run without Thal phase and Braak stage, and both with and without *APOE*ε2 and ε4 alleles, for comparison. Sex (male-only or female-only), and *APOE*ε4 (*APOE*ε4-pos, or *APOE*ε4-neg) stratified analysis were likewise performed, adjusting for the same covariates, excluding sex as appropriate. Sex and *APOE*ε4 interaction models were run in R (v3.5.2) by including an interaction term (SNP*Sex, or SNP*APOEε4) in the regression model. Key variants were tested for association with dyshoric CAA by creating a binary variable where individuals with an average CAA score of 0.5–3 were grouped together and compared to those with a score of 4, using logistic regression run in R.

For all analysis p-values are reported. To determine genome-wide significance we applied a p-value threshold of 2.97E-08 which applies a Bonferroni adjustment for 1,679,420 SNPs that remained after filtering on an r2 of 0.8 [19] Further adjustments for analyses of subsets of the dataset were not applied. Association results of variants with a dbSNP reference SNP identifier, v142 from GWAS and interaction analyses were tested for enrichment of gene sets in the Gene Ontology [4, 53] database using GSA-SNP2 [36] software with selected options of European, padding of 20 kb, build GRCh37 (hg19), and pathway size window of 10–200.

WGS genotypes at the *LINC-PINT* locus were assessed for MAF and linkage disequilibrium (LD) with the index SNP using PLINK [13]. SNPs were tested for association with CQN gene expression levels (eQTL) using a linear mixed model implemented with the lme4 package [6] in R statistical software version 3.5.2. CQN expression value was the dependent variable, variant dosage (0, 1 or 2) was the independent variable. Similarly, for exon QTL (splicing QTL = sQTL), the variant genotypes were tested for association with the normalized log2FPKM exon expression values. All QTL models were adjusted for diagnosis, sex, age at death, RIN, tissue source, flowcell and the first three principle components, with flowcell being the random effects variable. Denominator degrees of freedom for test statistic was obtained using Kenward-Roger [23] restricted maximum likelihood approximation in the lmerTest package [25] in R.

*LINC-PINT* expression levels were assessed for differential expression between AD cases and controls, and for association with the expressed transcriptome in the reprocessed AMP-AD datasets using linear regression implemented in R statistical software version 3.5.2. Normalized *LINC-PINT* expression measures were the dependent variable, and either diagnosis or normalized gene expression levels were the independent variable; all analyses were adjusted for age at death, sex, RNA integrity number (RIN), and sequencing batch. In compliance with HIPAA, samples with age over 90 were censored and coded as “90” in all datasets for the purpose of analysis. Gene sets were tested for enrichment of gene ontology (GO) terms using the “anRichment” R package with p-values computed via the hypergeometric test. Tests for enrichment of cell type marker genes were carried out using Fisher’s exact test and previously defined cell type marker genes [2, 61]. False-discovery rate adjusted (Benjamini-Hochberg) q-values were calculated using R, as appropriate.

REVIGO [50] was used to organize significant GO terms from GSA-SNP2 and “anRichment” outputs based on similarity, and to generate summary figures using the “treemap” package implemented in R statistical software version v3.6.2. REVIGO settings used were “medium (0.7)” for allowed similarity, “Homo Sapiens” (Gene Ontology Jan 2017) as the database and “SimRel for the semantic similarity measure.

### RNAscope

To further validate and visualize the RNAseq expression measures for *LINC-PINT* we performed RNAscope using cerebellum tissue from 8 AD cases that were part of the Mayo RNAseq study and identified as having high or low *LINC-PINT* expression. Single nuclei suspensions were collected from human cerebellum following an established approach [39]. Nuclei were then stained with anti-HNA (ab216943) antibodies and sorted using FANS (BD FACSAria™ II Cell Sorter) to increase purity. Sorted nuclei were seeded to PLL-coated 8-well chamber slides and fixed with 4% PFA for 60 min at room temperature. An RNA probe that targets the *LINC-PINT* transcript was utilized in RNAscope® Fluorescent Multiplex (ACDBiotech—477,631) assay according to manufacturer’s instructions. DAPI was used to mark and visualize the cerebellar nuclei and five images per condition were captured via 63X objective of Confocal Laser Scanning Microscope (Zeiss). Images were processed via ZEN Black software (Zeiss). Cell Profiler pipeline was established to relate and assign the dots to respective nuclei; the pipeline was applied to each image. *LINC-PINT* intensity per image was calculated according to scoring criteria developed by manufacturer and an H-score was assigned to each image. Mann–Whitney test was used to assess the statistical significance of the variation in H-score.

### non-AD dataset

The Mayo Clinic Brain Bank was queried to identify additional participants scored for CAA pathology, and an age at death of greater than 55 years, without a pathological diagnosis of Alzheimer’s disease (non-AD). Amongst these participants, 265 were identified with existing available genotypes from a prior GWAS [21], 100 with existing whole genome sequence genotypes (Mayo RNAseq, genetic data, Table S2), and 217 with available DNA for genotyping, resulting in a sample size of 582 non-AD individuals. Taqman genotyping assays (Thermo Fisher Scientific, USA) were not available for the index variant, rs10234094, so we elected to investigate a SNP, rs1588770, that is in strong linkage disequilibrium (r2 = 1, D’ = 1, in the AD dataset) and had an available assay. Genotypes for rs1588770 were extracted from the prior GWAS and WGS study data for 365 participants. For the remaining 217 individuals, DNA was genotyped on 384 well plates according to manufacturer’s directions using the QuantStudio 7 Flex system (Thermo Fisher Scientific, USA). Similarly genotypes for the *APOE* tagging variants rs429358 and rs7412 were extracted from existing GWAS or WGS data, or from an in-house database based on prior Taqman genotyping. Variant rs1588770 (dominant model) was tested for association with sqrtCAA in the *APOE*ε4 defined subsets (Table S1) using multi-variable linear regression, with Age at death, Sex, Braak stage and Thal phase included as covariates. *APOE*ε4 status ( ±) was similarly tested for association with sqrtCAA in the overall non-AD dataset. AD diagnosis was tested for association with sqrt CAA adjusting for Age, Sex, *APOE*ε2 and *APOE*ε4. All statistical analyses for the non-AD dataset were performed using R statistical software v4.0.2.

### Data sharing

The data in this manuscript are available via the AD Knowledge Portal (https://adknowledgeportal.synapse.org). The AD Knowledge Portal is a platform for accessing data, analyses and tools generated by the Accelerating Medicines Partnership (AMP-AD) Target Discovery Program and other National Institute on Aging (NIA)-supported programs to enable open- science practices and accelerate translational learning. The data, analyses and tools are shared early in the research cycle without a publication embargo on secondary use. Data is available for general research use according to the following requirements for data access and data attribution (https://adknowledgeportal.synapse.org/DataAccess/Instructions). For access to content described in this manuscript see https://doi.org/10.7303/syn22228853.

## Results

### Cerebral amyloid angiopathy associates with sex, ***APOE***ε4 and AD neuropathology

A total of 821 neuropathologically confirmed AD cases from the Mayo Clinic Brain bank that were scored for CAA and passed genetic data quality control (Additional file 1: Figure S1) were included in this study. Participants had a mean age at death of 80.1 years. There are slightly more females than males, and a range of ADNC (Braak stage and Thal phase) is represented (Table 1, Additional file 1: Table S1). We confirmed that increased CAA levels associated (p < 1E-02) with the known risk factors of male sex, *APOE*ε4 and ADNC (Additional file 1: Table S4 & Figures S5-S8). Stratification based on sex revealed that *APOE*ε4 dose is similarly associated with CAA in both males and females. Thal phase and Braak stage are associated with higher CAA in the *APOE*ε4 negative and positive subsets, respectively (Additional file 1: Figures S9-S10). These results suggest that the contribution of known risk factors to CAA may vary depending on the *APOE* genotype.

**Table 1:** Characteristics of the dataset

Subset	N	N: Sex (%)		N: APOE ε4 dose (%)			Mean age at death (sd)	Mean CAA (sd)	N: Thal (%)				N: Braak stage (%)		
		Male	Female	0	1	2			2	3	4	5	4	5	6
All	821	370 (45%)	451 (55%)	287 (35%)	414 (50%)	120 (15%)	80.1 (8.6)	0.85 (0.76)	3 (0%)	60 (7%)	69 (8%)	689 (84%)	98 (12%)	280 (34%)	443 (54%)
*APOE*ε4 pos (ε4 +)	534	241 (45%)	293 (55%)	0 (0%)	414 (78%)	120 (22%)	80.5 (8.2)	0.96 (0.77)	1 (0%)	33 (6%)	48 (9%)	452 (85%)	65 (12%)	165 (31%)	304 (57%)
*APOE*ε4 neg (ε4-)	287	129 (45%)	158 (55%)	287 (100%)	0 (0%)	0 (0%)	79.4 (9.2)	0.72 (0.70)	2 (1%)	27 (9%)	21 (7%)	237 (83%)	33 (12%)	115 (40%)	139 (48%)
Males	370	370 (100%)	0 (0%)	129 (35%)	180 (49%)	61 (16%)	78.4 (8.1)	0.98 (0.79)	1 (0%)	31 (8%)	33 (9%)	305 (82%)	53 (14%)	136 (37%)	181 (49%)
Females	451	0 (0%)	451 (100%)	158 (35%)	234 (52%)	59 (13%)	81.5 (8.7)	0.79 (0.72)	2 (0%)	29 (6%)	36 (8%)	384 (85%)	45 (10%)	144 (32%)	262 (58%)

Characteristics are provided for all AD individuals and each of the subsets defined by *APOE*e4 carrier status ( ±) and Sex (M/F). N = number, SD = standard deviation, Thal “L” refers to the two Thal phase levels defined as low, Thal “H” refers to the two Thal phase levels defined as high in the converted binary trait

### In AD patients, variants at the *APOE* locus represent the strongest genetic risk factor for CAA

To identify genetic risk factors for CAA we performed a genome-wide association study. Analysis models were run with and without adjustment for AD neuropathology (Thal phase and Braak stage), and *APOE*ε2 and *APOE*ε4 alleles. There was no evidence of genomic inflation (λ < 1.010) and the results from the tested models are highly similar (Additional file 1; Figures S11-S12). We focused on AD neuropathology adjusted models without *APOE* as our primary analysis, but provide results for the other models where appropriate. CAA was most significantly associated with rs5117 (beta = 0.18, *p* = 9.42E-18), located in an intron of *APOC1* (Additional file 1: Table S5 & Figures S13-S14)*.* This SNP is in linkage disequilibrium (r2 = 0.89, D’ = 0.98) with rs429358 which tags the *APOE*ε4 allele. Rs5117 is no longer significant following adjustment for *APOE*ε2 and ε4 alleles, but the most significant variant (rs35136575) in this model is also at the *APOE* locus. Rs35136575 has similar association results with CAA in both *APOE*ε2/ε4 adjusted and unadjusted models, beta = 0.13, *p = *3.24E-07; beta = 0.13, *p* = 8.61E-07, respectively (Additional file 1: Table S5 & Figure S15). This variant is not in LD with the *APOE*ε4 allele tagging variant rs429358 in our dataset (r2 = 0.00032, D’ = 0.03) indicating that it represents an association signal independent of *APOE* at this locus. The *APOE*ε4 allele has been implicated in capillary CAA [5, 41, 51]. In support of this we found significant association of the tagging variant rs429358 with a CAA score of 4 when compared to a score of 0.5–3 (*p* = 5.8E-03). However, this variant was also associated with CAA when individuals with a score of 4 were removed (*p* = 3.90e-14), indicating that the *APOE*e4 allele confers risk for CAA both in the presence and absence of dyshoric pathology in our dataset of AD cases, although its effect may be stronger in the former. Outside of chr19q13, no additional variants reach genome-wide significance (GWS), although 28 variants at 13 loci have *p*-values of < 1E-05 (Additional file 1 Table S6). The results for these variants are largely consistent regardless of the model considered.

Gene-set enrichment analysis for results from the primary model identified 43 significant (q < 0.05) gene ontology biological processes (GO-BP) that broadly organize into seven groups (Additional file 1: Figure S16). The most significant terms in each group include regulation of axonogenesis, positive regulation of excitatory postsynaptic potential, xenobiotic catabolism, social behavior, guanosine-containing compound metabolism, response to ischemia, and synaptic vesicle localization (Additional file 1: Table S7). Many of these GO terms remain significant when using results adjusted for *APOE*ε4 and ε2 alleles, indicating that the implicated pathways likely reflect genetic contributions to CAA etiology independent of *APOE* (Additional file 1: Figure S17).

## Variants at the ***LINC-PINT*** locus are associated with CAA in AD patients who lack the ***APOE***ε4 risk allele

To identify genetic risk factors for CAA that may differ based on the biological context, we performed genome-wide association analysis in sex- or *APOE*ε4-stratified datasets. We also completed SNP-interaction analyses with these variables. Variants in LD with the *APOE*ε4 tagging variant rs429358 represent the most significant locus when males and females were assessed separately (Additional file 1: Figures S18-S19). In the sex interaction analysis no variants reached GWS (*p* < 2.9E-08), although a total of 115 variants at 15 loci had a p-value < 1E-05 (Additional file 1: Table S8 & Figure S20).

**Fig. 1:**
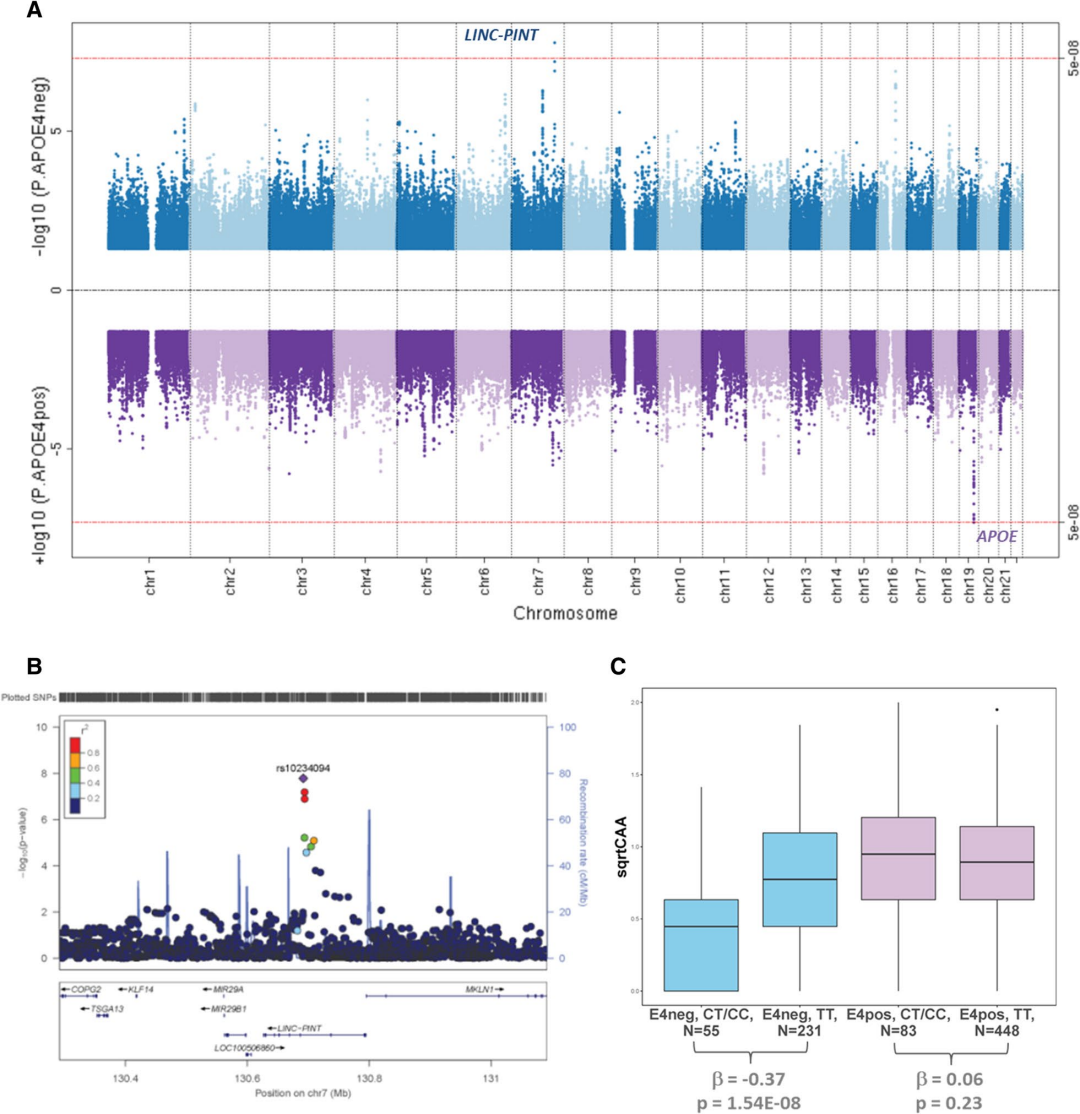
A variant at the *LINC-PINT* locus is associated with lower CAA levels in AD cases without the *APOE*ε4 risk allele. **A**. Miami plot illustrating results of genome-wide association study conducted in *APOE*ε4 non-carriers (ε4-neg, upper panel) and carriers (ε4-pos, lower panel) separately. **B**. Locus Zoom plot [37] showing association of variants at the *LINC-PINT* locus with CAA in the ε4-neg group. The most significant variant (rs10234094, Chr7: 130,961,759) is indicated in purple, with 500 kb flanking region 5’ and 3’ of this variant included in the plot. The association p-value is shown on the Y-axis and linear position on the chromosome on the X axis. Each point on the plot represents one variant; the colors of the points indicate the linkage disequilibrium (r2) value with the index variant (rs10234094). **C**. Boxplot illustrating distribution of sqrtCAA score (Y-axis) across 817 AD cases with respect to *APOE*ε4 carrier status and rs10234094 genotype under a dominant model (CT + CC vs TT). β = regression coefficient beta, *p = *p-value

Variants at the *APOE* locus were the most significant genetic risk factor for CAA in the *APOE*ε4pos subset. However, in the *APOE*ε4 negative (*APOE*ε4neg) subset we identified a genome-wide significant (GWS) association for variant rs10234094, located in an intron of *LINC-PINT* on chromosome 7. The minor allele of this variant is associated with lower CAA (beta =  − 0.37, *p = *1.63E-08, Fig. 1a, Table2). Rs10234094 was also the lead SNP in the *APOE*ε4 interaction analysis, (Additional file 1: Table S9 & Figure S21). The association of the *LINC-PINT* index variant with CAA remained significant in the analysis that utilized hard calls, instead of imputed dosages, and under a dominant model (Fig. 1b). The association was also consistent across the two genotyping batches (Additional file 1: Figure S22). Exclusion of individuals with the highest score of CAA (four) in any one brain region did not substantially impact the result (*p* = 5.25E-08). These findings indicate that the rs10234094-C allele affords protection from a higher burden of CAA pathology in AD cases in the absence of *APOE*ε4 and is unlikely to be driven by the presence of dyshoric CAA. This protective effect is negated by the strong risk of the *APOE*ε4 allele. Indeed, a single copy of the *APOE*ε4 allele is sufficient to neutralize the benefit of rs10234094-C (Additional file 1: Figures S23-S24).

**Table 2:** Genome-wide significant variants associated with CAA in AD cases identified at a novel locus *LINC-PINT* and the *APOE* locus

SNP ID	CHR	Position	Closest Gene	Function	A1	MAF	HWE *p*-value	APOE ε4 -ve		APOE ε4 + ve		All	
								BETA (95% CI)	*p*-value	BETA (95% CI)	*p*-value	BETA (95% CI)	*p*-value
rs10234094	7	130,691,759	*LINC-PINT*	ncRNA_intronic	C	0.09	5.10E-01	− **0.37 (-0.49—**− **0.24)**	**1.63E-08**	0.04 (-0.04—0.13)	3.02E-01	− 0.11 (-0.18—-0.03)	5.72E-03
rs5117	19	45,418,790	*APOC1*	intronic	C	0.41	2.73E-01	0.23 (0.04—0.42)	1.92E-02	**0.30 (0.37—0.23)**	**8.54E-15**	**0.19 (0.15—0.23)**	**9.42E-18**
rs429358	19	45,411,941	*APOE*	exonic	C	0.39	1.89E-01	0.57 (− 0.06—1.19)	7.55E-02	**0.32 (0.40—0.25)**	**9.35E-16**	**0.18 (0.14—0.23)**	**1.66E-16**

Gene-set enrichment analysis for the *APOE*ε4 and sex—interaction results indicates the presence of additional genetic changes in specific pathways that may drive context disparate vulnerability to CAA amongst AD cases. These include cell migration in sprouting angiogenesis, neuromuscular process and T cell costimulation (Additional file 1: Table S7 & Figure S25).

### CAA risk factors in the absence of ADNC

To determine the association of CAA risk factors in the absence of substantial ADNC we evaluated a group of 582 non-AD individuals from the Mayo Clinic Brain Bank, with available CAA scores. We observed a significant increase in CAA with *APOE*ε4 positivity (*p = *1.0E-07) amongst these participants, and these non-AD cases had significantly lower CAA than AD cases (*p = *1.74E-65). To evaluate the *LINC-PINT* locus we identified a variant, rs1588770, in strong LD (r2 = 1, D’ = 1) with the index variant. This variant was not associated with CAA in either of the *APOE*ε4 subsets (Figure S26). These results confirm that ADNC and *APOE*ε4 are risk factors for vascular amyloid, and indicate that in the absence of ADNC the *LINC-PINT* variant does not afford significant protection regardless of *APOE*ε4 genotype.

## *LINC-PINT* exon splicing is associated with the CAA GWAS index variant

**Fig. 2:**
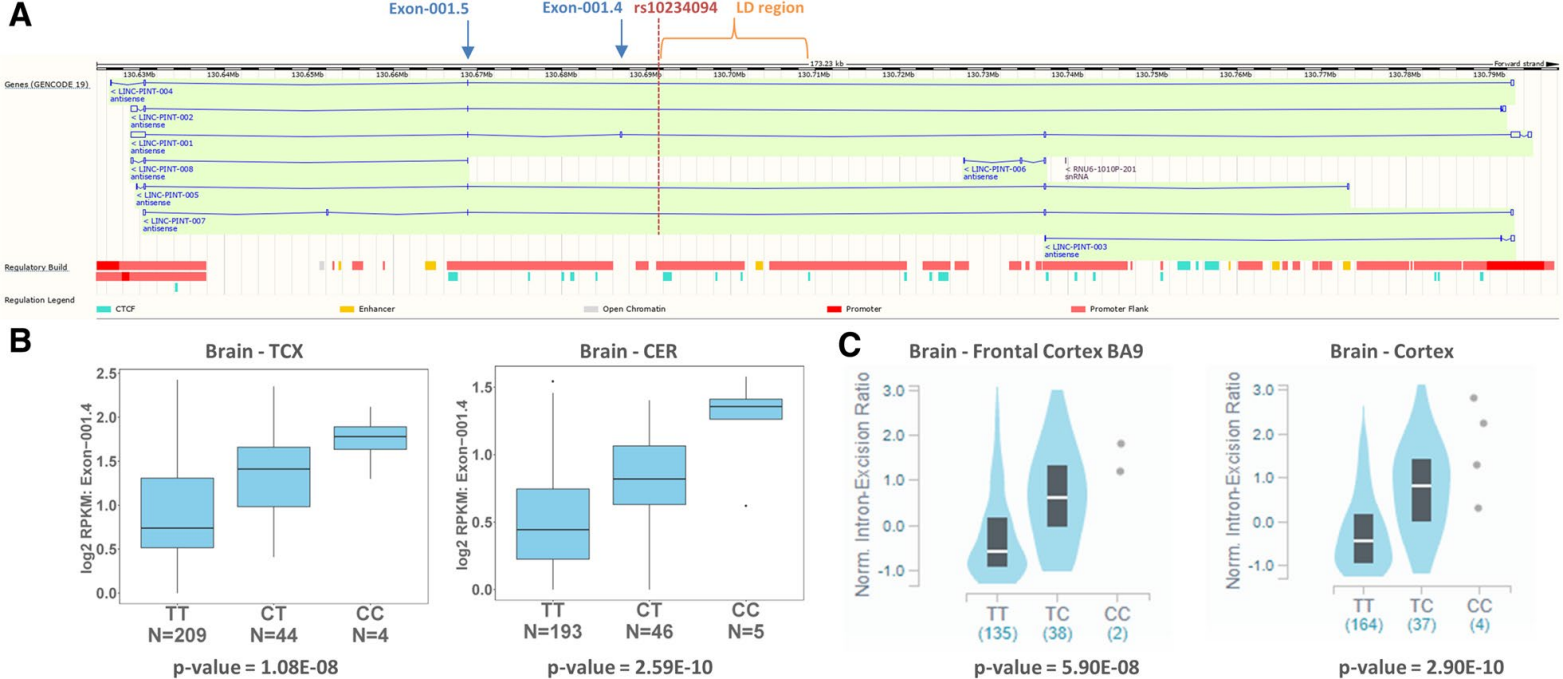
*LINC-PINT* variant rs10234094 is associated with alternative splicing of exon-001.4. **A**. *LINC-PINT* gene structure based on Ensembl [60] genome build GRCh37/hg19 (release 75)^26^, illustrates alternatively spliced exons, resulting transcripts, and Ensembl regulatory build annotation. Lead SNP, rs10234094, is indicated in red; the region identified to harbor SNPs in LD with the lead SNP is indicated in orange; Exon-001.4 (ENSE00001802751) and Exon-001.5 (ENSE00001786709) are indicated in blue. **B** normalized read counts that map to Exon-001.4 (ENSE00001802751) from RNAseq measures in temporal cortex (TCX) and cerebellum (CER) brain tissue are shown with respect to rs10234094 genotypes. **C** Normalized intron excision ratios for the intron between Exon-001.4 and Exon-001.5 from GTEx RNAseq data in Brain Frontal Cortex and Brain Cortex samples are shown with respect to rs10234094 genotypes, adapted from the GTEx online resource [15]. GTEx resource is based on genome build GRCh38/hg38 where co-ordinates for the intron between Exon-001.4 and Exon-001.5 are 130,984,109 to 131,002,197, which correspond to 130,686,955 to 130,668,869 for build GRCh37/hg19

To fine-map the *LINC-PINT* locus and explore the putative functional consequences of the lead variant, we used our existing data from a complementary study with available whole genome sequence (WGS) and RNASeq brain expression data from two brain regions (temporal cortex = TCX and cerebellum = CER; Additional file 1: Table S10). This dataset enables evaluation of a greater breadth of genetic variation at this locus, and assessment for association with gene expression measures collected from brain tissue. We identified 4,678 bi-allelic variants in the WGS data proximal (± 1 Mb) to the lead CAA GWAS variant, rs10234094, with a MAF ≥ 2%, of which nine met linkage disequilibrium (LD) thresholds of D’ ≥ 0.8 and r2 ≥ 0.2. These ten variants are located within a 17 kb window, in an intron of *LINC-PINT* (Fig. 2a). We hypothesized that these variants might influence brain gene expression of *LINC-PINT* or other proximal genes. Analysis of the nine genes *in-cis* with the lead SNP (± 1 MB) did not identify any eQTL that remained significant after Bonferroni adjustment for the number of tests. (Additional file 1: Table S11). *LINC-PINT* has 23 ensembl [60] defined exons (13 non-overlapping), some of which are alternatively spliced (Fig. 2a). Assessment of measured exon levels in TCX and CER revealed significantly higher levels of exon 4 (ENSE00001802751) in ensembl transcript 001 (ENST00000451786.1) associated with rs10234094-C, that remained significant after Bonferroni adjustment for 23 tests (Fig. 2b). Results from the GTEx portal (https://www.gtexportal.org/home/) provides strong independent support of these *LINC-PINT* splicing association results with rs10234094-C in brain tissue (Fig. 2c). No other *LINC-PINT* splicing events were associated with this variant in brain tissue [15] in GTEx or our dataset.

### Brain expression profiling of *LINC-PINT* reveals upregulation in AD and uncovers associated biological pathways

Increased brain expression of *LINC-PINT* has been previously reported for individuals with various neuropathological diseases, including AD [45]; given that ADNC is a risk factor for CAA, we decided to explore this further. We assessed levels of *LINC-PINT*, a non-coding RNA (ncRNA), for differential expression between neuropathological AD cases and controls across seven brain regions in a total of 1,186 samples from 800 individuals profiled as part of the AMP-AD consortium (Additional file 1: Table S12) [1, 16, 27, 56]. *LINC-PINT* expression was significantly higher in AD cases than controls in six of the brain regions, with a consistent direction of change across all seven (Table 3). This was further supported by RNAscope visualization of *LINC-PINT* expression in frozen cerebellum nuclei, from 4 cases with low and 4 cases with high expression based on prior RNAseq (Additional file 1: Figure S27).

**Table 3:** *LINC-PINT* (ENSG00000231721) has higher tissue level expression in neuropathologically defined AD cases than in controls

Dataset	Brain region	N	BETA (95%CI)	*p*-value
ROSMAP	DLPFC	475	0.09 (0.04—0.13)	2.91E-04
Mayo Clinic	CER	144	0.36 (0.20—0.52)	1.90E-05
Mayo Clinic	TCX	148	0.01 (-0.07—0.10)	7.95E-01
MSBB	BM10	124	0.15 (0.01—0.29)	3.26E-02
MSBB	BM22	103	0.24 (0.02—0.45)	3.07E-02
MSBB	BM36	86	0.20 (0.04—0.37)	1.45E-02
MSBB	BM44	106	0.29 (0.10—0.48)	3.03E-03

Differential expression assessed in seven datasets representing six brain regions. ROS = Religious Orders Study, MAP = Memory and Aging Project, MSBB = Mount Sinai Brain bank. CER = Cerebellum, TCX = Temporal Cortex, DLPFC = dorsolateral prefrontal cortex, BM = Brodmann area. se = standard error

**Fig. 3:**
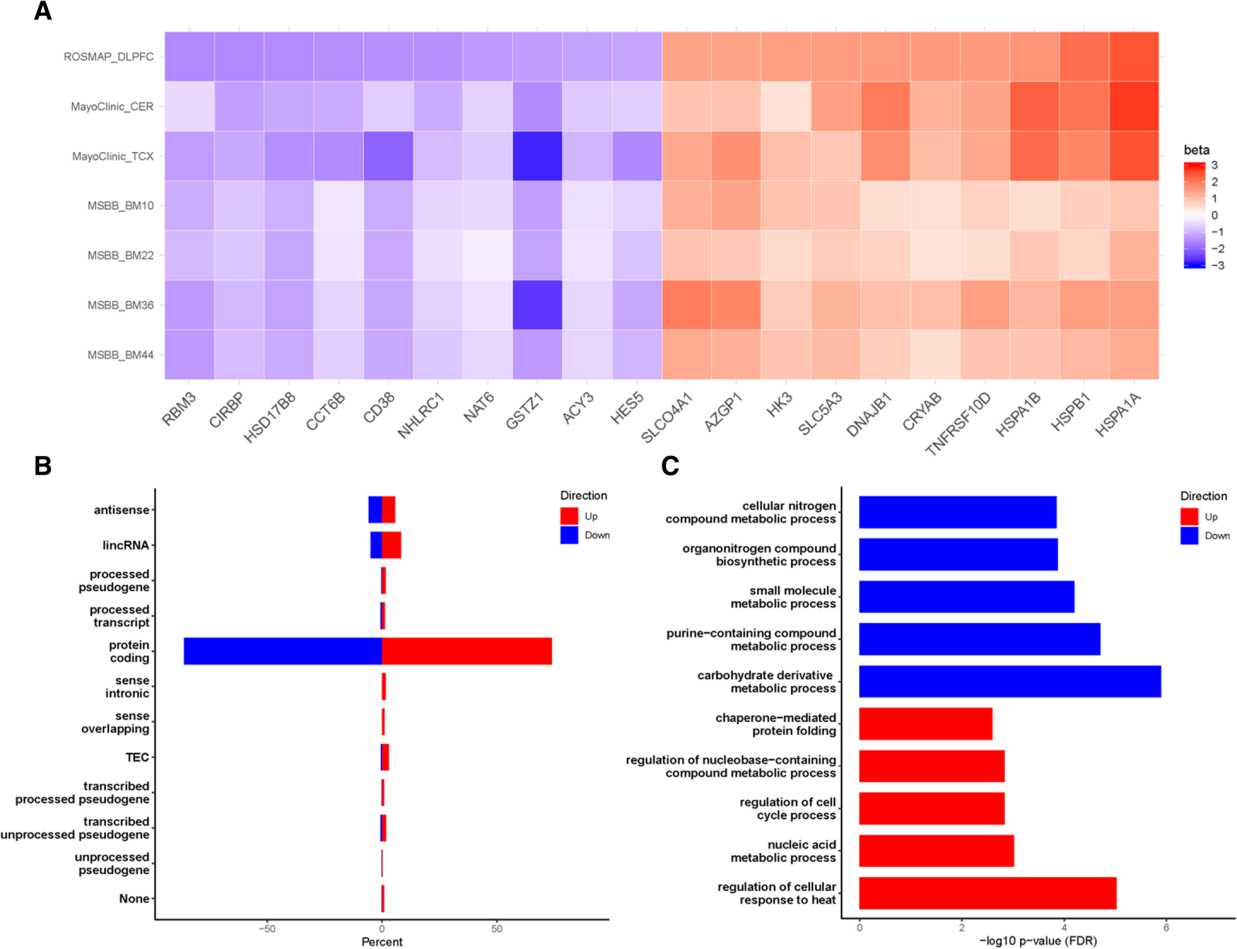
1,052 genes consistently associated with *LINC-PINT* expression across seven brain regions are enriched in gene ontology biological pathways.** A** Ten genes with the greatest degree of brain level correlations (beta) with *LINC-PINT* expression, either negatively (blue) or positively (red), in the largest dataset (ROSMAP) were selected. These genes have significant (*q* < 0.05) *LINC-PINT* level correlations across all datasets. The regression coefficient is shown in a heatmap for all seven brain regions; ROSMAP = religious order study (ROS) and memory and aging project (MAP); DLPFC = dorsolateral prefrontal cortex; CER = Cerebellum; TCX = Temporal Cortex; MSBB = Mount Sinai Brain Bank; BM = Brodmann area. **B**. Distribution of gene biotypes that are consistently negatively (blue) or positively (red) associated with *LINC-PINT* identifies a greater proportion of protein coding genes in the negatively associated gene set. Protein coding genes are expected to be over-represented overall due to three of the brain regions (DLPFC, TCX, CER) utilizing a poly-A selection library preparation approach. **C**. The five most significant (*q* < 0.05) gene ontology biological processes enriched for genes that are consistently negatively (blue) or positively (red) associated with *LINC-PINT* across seven brain regions. Biological process term is on the Y axis and the –log10 *p*-value for enrichment is on the X axis

We hypothesized that *LINC-PINT* levels may play a regulatory role and tested its levels for correlations with the expressed transcriptome across all AMP-AD datasets. There were 1,104 *LINC-PINT* correlated genes with a false-discovery rate (FDR) adjusted *q*-value < 0.05 in all seven brain regions. Of these, 95% were consistent in their direction of correlation. There were 494 genes with consistent positive (up regulated) and 558 with consistent negative correlations (downregulated) with brain *LINC-PINT* levels. Figure 3a depicts the top 10 correlated genes, in the largest dataset, ROSMAP DLPFC (N = 455), with consistent *LINC-PINT* correlations across all seven brain regions. *LINC-PINT* correlated genes were largely protein coding (86% of downregulated and 73% of upregulated genes); although other gene biotypes were also present (Fig. 3b). The sets of consistently up and down regulated genes were not enriched for cell-type specific marker genes [2, 61] indicating that these correlations are unlikely to reflect tissue co-expression due to predominant representation in a common cell type (data not shown). *LINC-PINT* correlated protein coding genes were evaluated for enrichment of gene ontology (GO) terms [4, 53]. This revealed biological pathways involved in metabolism, carbohydrate biosynthesis, ribosome biogenesis and chaperone-mediated protein transport enriched for downregulated genes (Fig. 3c, Additional file 1: Figure S28). Chromosome organization, cellular response to heat, cell cycle, protein folding and metabolism related terms are enriched for upregulated genes (Fig. 3c, Additional file 1: Figure S29). Many of the significant GO terms are enriched for both up and down regulated genes indicating these pathways may be particularly relevant to the brain expression and function of *LINC-PINT*.

## Discussion

We report the first genome-wide association study of quantitative CAA neuropathology in AD cases exclusively, and assessment of sex and *APOE*ε4 specific effects. Genome-wide significant association was identified at a novel locus, *LINC-PINT*—rs10234094*,* with lower CAA in *APOE*ε4 negative AD cases. Analysis of the brain transcriptome indicates that this intronic variant likely functions through altered splicing of a *LINC-PINT* exon. Further examination identified higher overall *LINC-PINT* levels in neuropathologic AD cases, and a set of 1,052 genes that show reproducible co-expression with *LINC-PINT* across seven brain regions. Pathway enrichment analysis implicates a role for *LINC-PINT* in protein folding and various metabolic processes in the brain.

*LINC-PINT*, located on chromosome 7q32.3, is a long non-coding RNA (lncRNA) that is regulated by p53 and implicated as a tumor suppressor in multiple forms of cancer, likely functioning via interaction with the Polycomb Repressive Complex 2 (PRC2) [30]. The GTEx resource [15] demonstrates *LINC-PINT* is expressed in multiple tissues and across multiple brain regions. Data from sorted populations of cells indicate that in the brain *LINC-PINT* has the highest expression in neuronal [45, 48] and microglial [61] cells; notably two cell types invariably involved in AD pathophysiology. In a recent lncRNA screen of brain tissue samples with multiple neurodegenerative conditions, increased expression of *LINC-PINT* was observed to be associated with Parkinson’s disease (PD), Huntington’s disease (HD) and AD [45]. *Linc-Pint* is also expressed in most mouse tissues including brain and its knock-out leads to growth retardation [42]. Knock-down of *LINC-PINT* in neuronal cells exacerbated cell death induced by oxidative stress, suggestive of a role for this lncRNA in neuroprotection [45].

More generally, a role for ncRNA in neurodegenerative diseases is emerging [58]. Many lncRNAs were found to be differentially expressed in brains of AD patients or mouse models and involved in biological processes including Aß metabolism, tau phosphorylation, neuroinflammation, synaptic plasticity and neuronal death [26]. Our results provide evidence for *LINC-PINT* specifically in modulating CAA pathology in AD. We nominate altered *LINC-PINT* splicing as the likely mechanism by which the index variant influences CAA. That we also find increased *LINC-PINT* in AD brains; as well as > 1,000 transcripts consistently correlated with it across seven brain regions suggest that this lncRNA may also have a broader role in AD pathophysiology.

The *LINC-PINT* locus was not previously identified in a GWAS of neuropathological AD endophenotypes that included CAA [7], however there are several key differences in the approaches used. The prior study included both AD cases and controls, a binary CAA phenotype (presence/absence) was employed, and neither sex nor *APOE*ε4- stratified analyses were reported. We were likewise unable to identify association of the *LINC-PINT* locus in a collection of individuals that did not meet neuropathological criteria for AD (non-AD). However we note that the level of CAA in these individuals is very low and postulate that the *LINC-PINT* variant may not afford discernable protection from vascular amyloid pathology in the absence of ADNC. Alternatively, any protection provided by this *LINC-PINT* variant may be difficult to detect due to the already lower levels of CAA in these non-AD individuals. Previous studies have indicated that *APOE*ε4 and ADNC are differentially associated with CAA when in the presence (CAA type I) or absence (CAA type II) of capillary involvement [5, 41, 51]. In the collection of AD cases studied here with advanced ADNC (88% Braak stage ≥ 5; 84% Thal phase = 5) a minority of cases (N = 11) had a CAA score of four, indicative of capillary involvement. The removal of these individuals did not substantially impact our key findings. Further studies in expanded cohorts will be needed to evaluate our findings in the context of disease (AD, non-AD, Control), capillary CAA, and disease severity (degree of AD neuropathologic change). The observed association for the *LINC-PINT* variant in AD cases lacking the *APOE*ε4 risk allele highlights the importance of stratification based on risk factors to discover novel loci that behave in a context-specific manner [22, 49].

Indeed, our study confirms that the *APOE* locus is the major genetic risk factor for CAA in AD cases both as a whole and in sex-stratified GWAS. Closer assessment of the locus identified a set of variants that maintain strong association with CAA after taking the *APOE*ε4 and ε2 alleles into account. The most significant of these, rs35136575 (*p = *3.24E-07), is located in the *APOE* hepatic control region 3’ of *APOE*, between *APOC1P1* and *APOC4*, and has previously been reported to associate with plasma levels of LDL-C and apoE, independent of *APOE*ε2/3/4 alleles [24]. These results lead to the hypothesis that variation at the *APOE* locus beyond *APOE*ε4 and ε2 influences risk for CAA. In-depth functional assessment of *APOE* locus variants including rs35136575 is needed to further define the mechanisms by which variation at this locus impacts CAA in AD.

The gene-set enrichment analysis results indicate that additional genetic variation likely contributes to CAA in AD cases. We identified several enriched biological processes related to neuronal/synaptic development and function. Dendritic spine and synaptic loss is reported in amyloid mouse models of AD as a consequence of amyloid pathology [47, 54], and neuronal death is a prominent feature of AD [46]. It should be noted that the gene-set enrichment results for CAA are driven by genetic associations and while they are not secondary to neuronal/synaptic loss due to AD pathology they may reflect selective vulnerability in the presence of CAA. We hypothesize this may occur as an event downstream of CAA, which induces reduced blood flow, or an upstream event leading to impairment in the neurovascular unit culminating in CAA [32]. Future studies should aim to determine if the observed gene-set enrichment is distinct to CAA, or common to accumulation of amyloid beta more generally, indicating a shared etiology with AD. This distinction may be important for application of biomarker or therapeutic strategies based on these findings.

There are multiple strengths to this study, including the focus on neuropathologically confirmed AD cases, the use of a continuous CAA phenotype, assessment of sex and *APOE*ε4-specific associations, and an integrative genomics approach that incorporates brain transcriptome and exon splicing data leading to mechanistic implications for the genetic findings. There are also several limitations. The sample size is relatively modest for a GWAS, although we utilized a quantitative trait that affords greater power than a dichotomous outcome [3]. This study was focused on individuals of northern European descent. It will be critical to extend this work to neuropathologic cohorts of non-European descent, as such cohorts become more available. The measures collected in this study are post-mortem and so reflect terminal neuropathological and transcriptional profiles. Future studies that evaluate neuroimaging outcomes reflective of CAA such as cerebral microbleeds and white matter hyperintensities can assess translation of our findings to the clinical disease course in AD. The neuropathological scale used to quantify extent of CAA does not account for CAA-related pathologic features such as concentric vessel splitting, fibrinoid necrosis, or paravascular blood deposits that reflect other aspects of CAA progression. Finally, while all AD cases met neuropathological criteria, many also harbor other neuropathological lesions [43]. Therefore, an imbalance of these or other comorbidities between subsets of individuals could potentially confound results, although our findings are robust to adjustment of Braak and Thal measures.

There are currently no treatments that can effectively delay or treat AD, of which CAA is an important component. We have characterized the genetic landscape of CAA in AD cases, providing evidence of additional genetic contribution to variability of this phenotype beyond *APOE*. We identified a novel CAA locus, *LINC-PINT* and a splice variant that attenuates CAA levels in AD patients lacking *APOE*ε4. Importantly this study provides biological insights that narrow the search space for identifying therapeutic targets to address this key neurovascular aspect of AD pathophysiology and highlights a precision medicine approach for future discoveries.

## Supplementary Information

**Additional file 1**.

## Data Availability

See “Data Sharing” section within Materials and Methods.

## Abbreviations

Aß: Amyloid ß
AD: Alzheimer’s disease
ADNC: Alzheimer’s disease neuropathologic change
CAA: Cerebral amyloid angiopathy
CER: Cerebellum
GWAS: Genome-wide association study
HRC: Haplotype reference consortium
MSBB: Mount Sinai Brain Bank
NFT: Neurofibrillary tangles
NP: Neuritic plaques
QTL: Quantitative trait locus
ROSMAP: Religious Orders Study and Rush Memory Aging Project
sqrtCAA: Square root transformed CAA scores
TCX: Temporal cortex
WGS: Whole-genome sequencing

## References

[1] Allen M, Carrasquillo MM, Funk C, Heavner BD, Zou F, Younkin CS (2016). Human whole genome genotype and transcriptome data for Alzheimer's and other neurodegenerative diseases. Sci Data.

[2] Allen M, Wang X, Burgess JD, Watzlawik J, Serie DJ, Younkin CS (2018). Conserved brain myelination networks are altered in Alzheimer's and other neurodegenerative diseases. Alzheimers Dement.

[3] Almasy L (2012). The role of phenotype in gene discovery in the whole genome sequencing era. Hum Genet.

[4] Ashburner M, Ball CA, Blake JA, Botstein D, Butler H, Cherry JM (2000). Gene ontology: tool for the unification of biology. Gene Ontol Consort Nat Genet.

[5] Attems J, Jellinger KA (2004). Only cerebral capillary amyloid angiopathy correlates with Alzheimer pathology–a pilot study. Acta Neuropathol.

[6] Bates D, Machler M, Bolker BM, Walker SC (2015). Fitting linear mixed-effects models using lme4. J Stat Softw.

[7] Beecham GW, Hamilton K, Naj AC, Martin ER, Huentelman M, Myers AJ (2014). Genome-wide association meta-analysis of neuropathologic features of Alzheimer's disease and related dementias. Plos Genet.

[8] Biffi A, Shulman JM, Jagiella JM, Cortellini L, Ayres AM, Schwab K (2012). Genetic variation at CR1 increases risk of cerebral amyloid angiopathy. Neurology.

[9] Boyle AP, Hong EL, Hariharan M, Cheng Y, Schaub MA, Kasowski M (2012). Annotation of functional variation in personal genomes using RegulomeDB. Genome Res.

[10] Boyle PA, Yu L, Nag S, Leurgans S, Wilson RS, Bennett DA (2015). Cerebral amyloid angiopathy and cognitive outcomes in community-based older persons. Neurology.

[11] Braak H, Braak E (1991). Neuropathological stageing of Alzheimer-related changes. Acta Neuropathol.

[12] Brenowitz WD, Nelson PT, Besser LM, Heller KB, Kukull WA (2015). Cerebral amyloid angiopathy and its co-occurrence with Alzheimer's disease and other cerebrovascular neuropathologic changes. Neurobiol Aging.

[13] Chang CC, Chow CC, Tellier LC, Vattikuti S, Purcell SM, Lee JJ (2015). Second-generation PLINK: rising to the challenge of larger and richer datasets. Gigascience.

[14] Chung J, Zhang X, Allen M, Wang X, Ma Y, Beecham G (2018). Genome-wide pleiotropy analysis of neuropathological traits related to Alzheimer's disease. Alzheimers Res Ther.

[15] Consortium GT, Laboratory DA, Coordinating Center -Analysis Working G, Statistical Methods groups-Analysis Working G, Enhancing Gg, Fund NIHC et al. (2017) Genetic effects on gene expression across human tissues. Nature 550:204–213. 10.1038/nature24277 10.1038/nature24277 PMC5776756 29022597

[16] De Jager PL, Ma Y, McCabe C, Xu J, Vardarajan BN, Felsky D (2018). A multi-omic atlas of the human frontal cortex for aging and Alzheimer's disease research. Sci Data.

[17] Dumitrescu L, Barnes LL, Thambisetty M, Beecham G, Kunkle B, Bush WS (2019). Sex differences in the genetic predictors of Alzheimer's pathology. Brain.

[18] Ellis RJ, Olichney JM, Thal LJ, Mirra SS, Morris JC, Beekly D (1996). Cerebral amyloid angiopathy in the brains of patients with Alzheimer's disease: the CERAD experience, Part XV. Neurology.

[19] Fadista J, Manning AK, Florez JC, Groop L (2016). The (in)famous GWAS P-value threshold revisited and updated for low-frequency variants. Eur J Hum Genet.

[20] Hansen KD, Irizarry RA, Wu Z (2012). Removing technical variability in RNA-seq data using conditional quantile normalization. Biostatistics.

[21] Hoglinger GU, Melhem NM, Dickson DW, Sleiman PM, Wang LS, Klei L (2011). Identification of common variants influencing risk of the tauopathy progressive supranuclear palsy. Nat Genet.

[22] Jun G, Ibrahim-Verbaas CA, Vronskaya M, Lambert JC, Chung J, Naj AC (2016). A novel Alzheimer disease locus located near the gene encoding tau protein. Mol Psychiatry.

[23] Kenward MG, Roger JH (1997). Small sample inference for fixed effects from restricted maximum likelihood. Biometrics.

[24] Klos K, Shimmin L, Ballantyne C, Boerwinkle E, Clark A, Coresh J (2008). APOE/C1/C4/C2 hepatic control region polymorphism influences plasma apoE and LDL cholesterol levels. Hum Mol Genet.

[25] Kuznetsova A, Brockhoff PB, Christensen RHB (2017). lmerTest package: tests in linear mixed effects models. J Stat Softw.

[26] Li D, Zhang J, Li X, Chen Y, Yu F, Liu Q (2020). Insights into lncRNAs in Alzheimer's disease mechanisms. RNA Biol.

[27] Logsdon B, Perumal TM, Swarup V, Wang M, Funk C, Gaiteri C et al. (2019) Meta-analysis of the human brain transcriptome identifies heterogeneity across human AD coexpression modules robust to sample collection and methodological approach. bioRxiv [Preprint]:10.1101/510420

[28] Loh PR, Danecek P, Palamara PF, Fuchsberger C, Y AR, H KF, (2016). Reference-based phasing using the Haplotype Reference Consortium panel. Nat Genet.

[29] Makela M, Kaivola K, Valori M, Paetau A, Polvikoski T, Singleton AB (2018). Alzheimer risk loci and associated neuropathology in a population-based study (Vantaa 85+). Neurol Genet.

[30] Marin-Bejar O, Marchese FP, Athie A, Sanchez Y, Gonzalez J, Segura V (2013). Pint lincRNA connects the p53 pathway with epigenetic silencing by the Polycomb repressive complex 2. Genome Biol.

[31] McKhann G, Drachman D, Folstein M, Katzman R, Price D, Stadlan EM (1984). Clinical diagnosis of Alzheimer's disease: report of the NINCDS-ADRDA Work Group under the auspices of Department of Health and Human Services Task Force on Alzheimer's Disease. Neurology.

[32] Montagne A, Zhao Z, Zlokovic BV (2017). Alzheimer's disease: A matter of blood-brain barrier dysfunction?. J Exp Med.

[33] Mostafavi S, Gaiteri C, Sullivan SE, White CC, Tasaki S, Xu J (2018). A molecular network of the aging human brain provides insights into the pathology and cognitive decline of Alzheimer's disease. Nat Neurosci.

[34] Murray ME, Graff-Radford NR, Ross OA, Petersen RC, Duara R, Dickson DW (2011). Neuropathologically defined subtypes of Alzheimer's disease with distinct clinical characteristics: a retrospective study. Lancet Neurol.

[35] Murray ME, Lowe VJ, Graff-Radford NR, Liesinger AM, Cannon A, Przybelski SA (2015). Clinicopathologic and 11C-Pittsburgh compound B implications of Thal amyloid phase across the Alzheimer's disease spectrum. Brain.

[36] Nam D, Kim J, Kim SY, Kim S (2010). GSA-SNP: a general approach for gene set analysis of polymorphisms. Nucleic Acids Res.

[37] Pruim RJ, Welch RP, Sanna S, Teslovich TM, Chines PS, Gliedt TP (2010). LocusZoom: regional visualization of genome-wide association scan results. Bioinformatics.

[38] Purcell S, Neale B, Todd-Brown K, Thomas L, Ferreira MA, Bender D (2007). PLINK: a tool set for whole-genome association and population-based linkage analyses. Am J Hum Genet.

[39] Renthal W, Boxer LD, Hrvatin S, Li E, Silberfeld A, Nagy MA (2018). Characterization of human mosaic Rett syndrome brain tissue by single-nucleus RNA sequencing. Nat Neurosci.

[40] Rentzsch P, Witten D, Cooper GM, Shendure J, Kircher M (2019). CADD: predicting the deleteriousness of variants throughout the human genome. Nucleic Acids Res.

[41] Richard E, Carrano A, Hoozemans JJ, van Horssen J, van Haastert ES, Eurelings LS (2010). Characteristics of dyshoric capillary cerebral amyloid angiopathy. J Neuropathol Exp Neurol.

[42] Sauvageau M, Goff LA, Lodato S, Bonev B, Groff AF, Gerhardinger C (2013). Multiple knockout mouse models reveal lincRNAs are required for life and brain development. Elife.

[43] Schneider JA, Arvanitakis Z, Bang W, Bennett DA (2007). Mixed brain pathologies account for most dementia cases in community-dwelling older persons. Neurology.

[44] Shinohara M, Murray ME, Frank RD, Shinohara M, DeTure M, Yamazaki Y (2016). Impact of sex and APOE4 on cerebral amyloid angiopathy in Alzheimer's disease. Acta Neuropathol.

[45] Simchovitz A, Hanan M, Yayon N, Lee S, Bennett ER, Greenberg DS (2020). A lncRNA survey finds increases in neuroprotective LINC-PINT in Parkinson's disease substantia nigra. Aging Cell.

[46] Spires TL, Hyman BT (2004). Neuronal structure is altered by amyloid plaques. Rev Neurosci.

[47] Spires TL, Meyer-Luehmann M, Stern EA, McLean PJ, Skoch J, Nguyen PT (2005). Dendritic spine abnormalities in amyloid precursor protein transgenic mice demonstrated by gene transfer and intravital multiphoton microscopy. J Neurosci.

[48] Srinivasan K, Friedman BA, Larson JL, Lauffer BE, Goldstein LD, Appling LL (2016). Untangling the brain's neuroinflammatory and neurodegenerative transcriptional responses. Nat Commun.

[49] Strickland SL, Reddy JS, Allen M, N'Songo A, Burgess JD, Corda MM (2020). MAPT haplotype-stratified GWAS reveals differential association for AD risk variants. Alzheimers Dement.

[50] Supek F, Bosnjak M, Skunca N, Smuc T (2011). REVIGO summarizes and visualizes long lists of gene ontology terms. PLoS ONE.

[51] Thal DR, Ghebremedhin E, Rub U, Yamaguchi H, Del Tredici K, Braak H (2002). Two types of sporadic cerebral amyloid angiopathy. J Neuropathol Exp Neurol.

[52] Thal DR, Rub U, Orantes M, Braak H (2002). Phases of A beta-deposition in the human brain and its relevance for the development of AD. Neurology.

[53] The Gene Ontology C (2019). The Gene Ontology Resource: 20 years and still going strong. Nucleic Acids Res.

[54] Tsai J, Grutzendler J, Duff K, Gan WB (2004). Fibrillar amyloid deposition leads to local synaptic abnormalities and breakage of neuronal branches. Nat Neurosci.

[55] Wan YW, Al-Ouran R, Mangleburg CG, Perumal TM, Lee TV, Allison K (2020). Meta-analysis of the Alzheimer's disease human brain transcriptome and functional dissection in mouse models. Cell Rep.

[56] Wang M, Beckmann ND, Roussos P, Wang E, Zhou X, Wang Q (2018). The Mount Sinai cohort of large-scale genomic, transcriptomic and proteomic data in Alzheimer's disease. Sci Data.

[57] Wigginton JE, Cutler DJ, Abecasis GR (2005). A note on exact tests of Hardy–Weinberg equilibrium. Am J Hum Genet.

[58] Wu YY, Kuo HC (2020). Functional roles and networks of non-coding RNAs in the pathogenesis of neurodegenerative diseases. J Biomed Sci.

[59] Yang HS, White CC, Chibnik LB, Klein HU, Schneider JA, Bennett DA (2017). UNC5C variants are associated with cerebral amyloid angiopathy. Neurol Genet.

[60] Yates AD, Achuthan P, Akanni W, Allen J, Allen J, Alvarez-Jarreta J (2020). Ensembl 2020. Nucleic Acids Res.

[61] Zhang Y, Sloan SA, Clarke LE, Caneda C, Plaza CA, Blumenthal PD (2016). Purification and characterization of progenitor and mature human astrocytes reveals transcriptional and functional differences with mouse. Neuron.

