# Supplementary material for: Genome-wide analysis identifies a novel LINC-PINT splice variant associated with vascular amyloid pathology in Alzheimer’s disease
Source: Acta Neuropathol Commun. 2021 May 21;9:93. doi: 10.1186/s40478-021-01199-2 (PMC8147512; doi:10.1186/s40478-021-01199-2)
Supplement: Supplementary file 1 — Additional file 1. [file 40478_2021_1199_MOESM1_ESM.pdf]

Genome-wide analysis identifies a *LINC-PINT* splice variant associated with vascular amyloid pathology in Alzheimer's disease.

Authors

Joseph S. Reddy Ph.D[1#], Mariet Allen Ph.D[2#], Charlotte C.G Ho[2], Stephanie R. Oatman[2], Özkan İş Ph.D[2], Zachary S. Quicksall[1], Xue Wang Ph.D[1], Jiangli Jin M.D[2], Tulsi A. Patel Ph.D[2], Troy P. Carnwath[2], Thuy T. Nguyen[2], Kimberly G. Malphrus[2], Sarah J. Lincoln[2], Minerva M. Carrasquillo Ph.D[2], Julia E. Crook Ph.D[1], Takahisa Kanekiyo M.D, Ph.D[2], Melissa E. Murray Ph.D[2], Guojun Bu Ph.D[2], Dennis W. Dickson M.D[2], Nilüfer Ertekin-Taner M.D, Ph.D[2,3, *]

Author Affiliations:

1) Mayo Clinic, Quantitative Health Sciences, Jacksonville, FL 32224 USA

2) Mayo Clinic, Department of Neuroscience, Jacksonville, FL 32224 USA

3) Mayo Clinic, Department of Neurology, Jacksonville, FL 32224 USA.

# Authors contributed equally.

* Corresponding Author

Corresponding Author Contact Information:

Mayo Clinic, Departments of Neurology and Neuroscience, 4500 San Pablo Road, Birdsall 3, Jacksonville, FL 32224. E-mail: taner.nilufer@mayo.edu, Phone: 904-953-7103, FAX: 904-953-7353.

Batch	Subset	N	N: Sex (%)		N: APOEε4 dose (%)			Mean age at death (SD)	Mean CAA (SD)	N: Thal (%)						N: Braak stage (%)						
			Male	Female	0	1	2			0 (L)	1 (L)	2 (M)	3 (M)	4 (H)	5 (H)	0 (L)	1 (L)	2 (L)	3 (L)	4	5	6
A	All	460	216 (47%)	244 (53%)	151 (33%)	246 (53%)	63 (14%)	80.2 (9.1)	0.82 (0.68)	0 (0%)	0 (0%)	3 (1%)	32 (7%)	38 (8%)	387 (84%)	0 (0%)	0 (0%)	0 (0%)	0 (0%)	69 (15%)	147 (32%)	244 (53%)
	Males	216	216 (100%)	0 (0%)	71 (33%)	113 (52%)	32 (15%)	78.5 (8.8)	0.91 (0.69)	0 (0%)	0 (0%)	1 (0%)	18 (8%)	16 (7%)	181 (84%)	0 (0%)	0 (0%)	0 (0%)	0 (0%)	40 (19%)	76 (35%)	100 (46%)
	Females	244	0 (0%)	244 (100%)	80 (33%)	133 (54%)	31 (13%)	81.8 (9.1)	0.75 (0.66)	0 (0%)	0 (0%)	2 (1%)	14 (6%)	22 (9%)	206 (84%)	0 (0%)	0 (0%)	0 (0%)	0 (0%)	29 (12%)	71 (29%)	144 (59%)
	APOEε4 pos (E4+)	309	145 (47%)	164 (53%)	0 (0%)	246 (80%)	63 (20%)	80.8 (8.7)	0.92 (0.70)	0 (0%)	0 (0%)	1 (0%)	15 (5%)	28 (9%)	265 (86%)	0 (0%)	0 (0%)	0 (0%)	0 (0%)	48 (16%)	87 (28%)	174 (56%)
	APOEε4 neg (E4-)	151	71 (47%)	80 (53%)	151 (100%)	0 (0%)	0 (0%)	79.1 (9.8)	0.63 (0.58)	0 (0%)	0 (0%)	2 (1%)	17 (11%)	10 (7%)	122 (81%)	0 (0%)	0 (0%)	0 (0%)	0 (0%)	21 (14%)	60 (40%)	70 (46%)
B	All	361	154 (43%)	207 (57%)	136 (38%)	168 (46%)	57 (16%)	80.0 (7.9)	0.94 (0.84)	0 (0%)	0 (0%)	0 (0%)	28 (8%)	31 (9%)	302 (84%)	0 (0%)	0 (0%)	0 (0%)	0 (0%)	29 (8%)	133 (37%)	199 (55%)
	Males	154	154 (100%)	0 (0%)	58 (38%)	67 (44%)	29 (19%)	78.4 (7.0)	1.07 (0.91)	0 (0%)	0 (0%)	0 (0%)	13 (8%)	17 (11%)	124 (81%)	0 (0%)	0 (0%)	0 (0%)	0 (0%)	13 (8%)	60 (39%)	81 (53%)
	Females	207	0 (0%)	207 (100%)	78 (38%)	101 (49%)	28 (14%)	81.1 (8.3)	0.84 (0.77)	0 (0%)	0 (0%)	0 (0%)	15 (7%)	14 (7%)	178 (86%)	0 (0%)	0 (0%)	0 (0%)	0 (0%)	16 (8%)	73 (35%)	118 (57%)
	APOEε4 pos (E4+)	225	96 (43%)	129 (57%)	0 (0%)	168 (75%)	57 (25%)	80.1 (7.5)	1.02 (0.85)	0 (0%)	0 (0%)	0 (0%)	18 (8%)	20 (9%)	187 (83%)	0 (0%)	0 (0%)	0 (0%)	0 (0%)	17 (8%)	78 (35%)	130 (58%)
	APOEε4 neg (E4-)	136	58 (43%)	78 (57%)	136 (100%)	0 (0%)	0 (0%)	79.7 (8.5)	0.82 (0.81)	0 (0%)	0 (0%)	0 (0%)	10 (7%)	11 (8%)	115 (85%)	0 (0%)	0 (0%)	0 (0%)	0 (0%)	12 (9%)	55 (40%)	69 (51%)
nAD	All	582	317 (55%)	265 (45%)	455 (78%)	118 (20%)	9 (2%)	75.3 (7.8)	0.20 (0.45)	239 (41%)	126 (22%)	53 (9%)	122 (21%)	27 (5%)	15 (3%)	91 (16%)	69 (12%)	234 (40%)	151 (26%)	36 (6%)	0 (0%)	1 (0%)
	Males	317	317 (100%)	0 (0%)	244 (77%)	68 (21%)	5 (2%)	74.6 (7.2)	0.22 (0.46)	135 (43%)	77 (24%)	23 (7%)	62 (20%)	14 (4%)	6 (2%)	57 (18%)	32 (10%)	134 (42%)	80 (25%)	13 (4%)	0 (0%)	1 (0%)
	Females	265	0 (0%)	265 (100%)	211 (80%)	50 (19%)	4 (2%)	76.3 (8.5)	0.18 (0.41)	104 (39%)	49 (19%)	30 (11%)	60 (23%)	13 (5%)	9 (3%)	34 (13%)	37 (14%)	100 (38%)	71 (27%)	23 (9%)	0 (0%)	0 (0%)
	APOEε4 pos (E4+)	127	73 (57%)	54 (43%)	0 (0%)	118 (93%)	9 (7%)	74.4 (8.1)	0.69 (0.46)	16 (13%)	25 (20%)	16 (13%)	45 (35%)	15 (12%)	10 (8%)	20 (16%)	10 (8%)	52 (41%)	34 (27%)	11 (9%)	0 (0%)	0 (0%)
	APOEε4 neg (E4-)	455	244 (54%)	211 (46%)	455 (100%)	0 (0%)	0 (0%)	75.6 (7.8)	0.30 (0.13)	223 (49%)	101 (22%)	37 (8%)	77 (17%)	12 (3%)	5 (1%)	71 (16%)	59 (13%)	182 (40%)	117 (26%)	25 (5%)	0 (0%)	1 (0%)

Table S1. Characteristics of the genotyping batches A and B presented separately and the non-AD follow up dataset. Genome-wide genotyping was completed on AD cases in two batches referred to as Batch A and Batch B. A separate non-AD dataset was utilized for assessment of key CAA risk factors. Dataset characteristics are provided for all individuals and each of the subsets defined by Sex (M/F) and *APOE*ε4 carrier status (+/-). N = number, SD = standard deviation, Thal "L" refers to the two Thal phase levels defined as low, Thal "M" refers to the two Thal phases defined as medium, Thal "H" refers to the two Thal phase levels defined as high. Similarly Braak stages 0-3 were classified as "low (L) and grouped together. Any potential genotyping batch (A and B) differences for key variables were assessed (statistical analysis). There are more cases with a Braak stage of four in genotyping batch A (p-value = 2.2E-03). None of the other variables were significantly different, indicating absence of systematic differences between the two genome-wide genotyping batches.

Dataset	Data Type	Description	SynapseID	DoD	Utility
MC-CAA	Metadata	Individual human	syn22228853	na	CAA-GWAS
MC-CAA	Metadata	Assay SNP array, Batches A and B	syn22228853	na	CAA-GWAS
MC-CAA	Genetic Data	Genotyped variant genotypes, Batch A	syn22228853	na	CAA-GWAS
MC-CAA	Genetic Data	Imputed Variant Dosages, Batch A	syn22228853	na	CAA-GWAS
MC-CAA	Genetic Data	Genotyped variant genotypes, Batch B	syn22228853	na	CAA-GWAS
MC-CAA	Genetic Data	Imputed Variant Dosages, Batch B	syn22228853	na	CAA-GWAS
MC-CAA	Analysis Results	CAA GWAS from primary model	syn22228853	na	CAA-GWAS
Mayo RNAseq TCX	RNASeq Expression	CQN normalized gene counts	syn22228853	na	eQTL
Mayo RNAseq CER	RNASeq Expression	CQN normalized gene counts	syn22228853	na	eQTL
Mayo RNAseq TCX	RNASeq Expression	RPKM exon counts, CQN *LINC-PINT*	syn22228853	na	sQTL, eQTL
Mayo RNAseq CER	RNASeq Expression	RPKM exon counts, CQN *LINC-PINT*	syn22228853	na	sQTL, eQTL
Mayo RNAseq TCX	Metadata	Individual human and RNAseq	syn22228853	na	eQTL, sQTL, DEG
Mayo RNAseq CER	Metadata	Individual human and RNAseq	syn22228853	na	eQTL, sQTL, DEG
Mayo RNAseq	Genetic Data	WGS genotypes[a]	syn22228853	na	eQTL and sQTL
Mayo RNAseq TCX	RNASeq Expression	Consensus processed RNASeq raw counts	syn8690799	10/2/2019	*LINC-PINT* DEG
Mayo RNAseq CER	RNASeq Expression	Consensus processed RNASeq raw counts	syn8690904	10/2/2019	*LINC-PINT* DEG
ROSMAP	RNASeq Expression	Consensus processed RNASeq raw counts	syn8691134	10/2/2019	*LINC-PINT* DEG
ROSMAP	Metadata	ID Key	syn3382527	10/2/2019	*LINC-PINT* DEG
ROSMAP	Metadata	Individual human	syn3191087	10/2/2019	*LINC-PINT* DEG
ROSMAP	Metadata	Assay RNAseq	syn21088596	1/2/2020	*LINC-PINT* DEG
MSBB	RNASeq Expression	Consensus processed RNASeq raw counts	syn8691099	10/2/2019	*LINC-PINT* DEG
MSBB	Metadata	Individual human	syn6101474	11/22/2019	*LINC-PINT* DEG
MSBB	Metadata	Assay RNAseq	syn6100548	10/2/2019	*LINC-PINT* DEG

Table S2: Data from AMP-AD knowledge portal utilized in this study. All data utilized in this study has either been generated by the study authors, is available on the AMP-AD knowledge portal, or both. Dataset names correspond to those referenced throughout the manuscript. DoD = Date of download, "na" indicates data that was generated by study authors and shared within the AMP-AD knowledge portal. SynapseID's can be searched directly within synapse only (https://www.synapse.org/#!Synapse:syn2580853/wiki/409840). **a**: WGS genotypes utilized in this study were generated through independent processing of raw sequence data as outlined in **Figure S3**. Note that we provide the synapse ID of VCF files generated by a separate AMP-AD pipeline for reference. For access to content described in this manuscript see https://doi.org/10.7303/syn22228853.

Dataset	Mayo RNAseq[g]	Mayo RNAseq[g]	ROSMAP[h]	MSBB[i]	MSBB[i]	MSBB[i]	MSBB[i]
**Brain Region**	TCX	CER	DLPFC	BM10	BM22	BM36	BM44
**N: GeneCounts[a]**	**278**	**278**	**639**	**265**	**264**	**267**	**230**
**N: Metadata[b]**	0	0	4	3	5	4	2
**N: Sex check[c]**	2	3	1	3	0	0	0
**N: RIN[d]**	0	0	0	34	38	48	37
**N: PCA outlier[e]**	2	3	3	0	0	0	0
**N: Other[f]**	15	26	0	7	33	48	5
**N: Retained**	**259**	**246**	**631**	**218**	**188**	**167**	**186**
**N: AD**	80	79	295	80	70	56	73
**N: Con**	68	65	180	44	33	30	33
**N: Other/unknown**	111	102	156	94	85	81	80

Table S3: AMP-AD reprocessed RNASeq datasets from Mayo Clinic, ROSMAP and MSBB: Quality Control and Diagnosis. Gene count and metadata files were downloaded from the AMP-AD knowledge portal (**Table S2**) and subject to inspection and quality control. **N**= number; **a**: unique samples ID's per dataset and brain region in the downloaded gene count files; **b**: samples with RNASeq gene counts that were either missing from metadata files, or had inconsistent values; **c**: samples with inconsistent sex between that indicated in the metadata files and that inferred based on expression of Y chromosome genes; **d**: samples with a RIN< 5; **e**: samples identified as gene expression outliers based on principal components analysis (>4SD from mean, PC1 or PC2) of the reprocessed gene counts (counts per million); **f**: samples flagged based on study-specific criteria. For Mayo Clinic these include samples listed in QC tables on the AMP-AD knowledge portal (**Table S2**), with the exception of four TCX and three CER samples where QC criteria are specific to genetic data; for MSBB these include samples with rRNA >5% and samples with the lowest number of reads for each pair of samples with two sets of RNASeq data from a unique individual (duplicates); samples listed in rows b-f were excluded from analysis. **g**: Mayo RNAseq[5] samples were diagnosed by a single neuropathologist according to NINCDS-ADRDA[16] criteria, where AD's have a Braak stage ≥4, while controls had a Braak stage ≤ 3; **h**: for the purpose of this study, ROSMAP[17] participants were diagnosed based on available neuropathological data, where AD cases have a Braak stage ≥4 and CERAD score ≤ 2, while controls a had Braak stage ≤ 3 and CERAD score ≥3; **i**: MSBB[18] individuals were classified as AD with Braak ≥ 4, and CERAD neuropathology category ≥ 2; controls were defined as Braak ≤ 3, and CERAD neuropathology category = 1. Note that ROSMAP[17] and MSBB[18] categorize CERAD[19] scores differently. Samples not fitting the diagnostic criteria, missing the necessary metadata, or with other defined diagnosis[5] were designated as "other/unknown" and excluded from our analyses.

Variable	All (N=821)		Females only (N=451)		Males only (N=370)		*APOE* ε4pos (N=534)		*APOE* ε4neg (N=287)	
	beta (95% CI)	p-value	beta (95% CI)	p-value	beta (95% CI)	p-value	beta (95% CI)	p-value	beta (95% CI)	p-value
Age	0.00 (-0.00, 0.01)	1.02E-01	0.00 (-0.00, 0.01)	1.21E-01	0.00 (-0.00, 0.01)	4.80E-01	0.01 (0.00, 0.01)	2.31E-02	0.00 (-0.01, 0.01)	7.49E-01
Sex (F)	-0.12 (-0.18, -0.06)	**4.73E-05**	-	-	-	-	-0.14 (-0.21, -0.07)	**1.86E-04**	-0.11 (-0.22, -0.00)	4.36E-02
APOE ε4 dose	0.19 (0.14, 0.23)	**5.13E-17**	0.19 (0.13, 0.25)	**1.56E-09**	0.18 (0.12, 0.24)	**9.84E-09**	-	-	-	
Thal Phase[a]	0.20 (0.09, 0.32)	**3.63E-04**	0.17 (0.01, 0.33)	3.57E-02	0.24 (0.08, 0.39)	**3.40E-03**	0.08 (-0.07, 0.23)	2.89E-01	0.37 (0.18, 0.55)	**1.22E-04**
Braak stage 5	0.11 (0.01, 0.21)	2.81E-02	0.09 (-0.05, 0.23)	2.21E-01	0.12 (-0.01, 0.26)	7.95E-02	0.13 (0.01, 0.25)	3.39E-02	0.07 (-0.12, 0.25)	4.67E-01
Braak stage 6	0.12 (0.02, 0.22)	1.56E-02	0.08 (-0.06, 0.22)	2.52E-01	0.15 (0.02, 0.29)	2.93E-02	0.16 (0.04, 0.27)	**8.11E-03**	0.08 (-0.11, 0.27)	4.09E-01

Table S4. Key variables associated with CAA in AD cases. Association of known risk factors for CAA variables were tested in the overall group of AD cases and in subsets of the data using multi-variable linear regression. Results presented are for the model including all variables listed. N = number, F= Female, a = Samples with a Thal phase of 2 or 3 were grouped as “Thal Low”, while Thal phase of 4 or 5 were grouped as “Thal High”. p-value < 1E-02 are bolded to indicate significance after taking 5 variables being tested into account.

SNP	CHR	POS (hg19)	Closest Gene	Function	Tested allele	MAF	HWE-P	All, Braak, Thal adj		All, Braak, Thal, APOE adj		All		All, APOE adj	
								Beta (95%CI)	p-value	Beta (95%CI)	p-value	Beta (95%CI)	p-value	Beta (95%CI)	p-value
rs12972156	19	45387459	*NECTIN2*	intronic	G	0.33	0.20	0.12 (0.08 - 0.17)	5.69E-08	-0.01 (-0.08 - 0.04)	5.60E-01	0.13 (0.09 - 0.18)	1.18E-08	-0.01 (-0.08 - 0.04)	6.16E-01
rs12972970	19	45387596	*NECTIN2*	intronic	A	0.33	0.20	0.12 (0.08 - 0.17)	5.55E-08	-0.01 (-0.08 - 0.04)	5.63E-01	0.13 (0.09 - 0.18)	1.15E-08	-0.01 (-0.08 - 0.04)	6.19E-01
rs34342646	19	45388130	*NECTIN2*	intronic	A	0.33	0.17	0.12 (0.08 - 0.17)	5.74E-08	-0.01 (-0.08 - 0.04)	5.65E-01	0.13 (0.09 - 0.18)	1.17E-08	-0.01 (-0.08 - 0.04)	6.24E-01
rs283815	19	45390333	*NECTIN2*	intronic	G	0.41	0.05	0.16 (0.11 - 0.20)	6.81E-13	0.01 (-0.06 - 0.09)	6.77E-01	0.17 (0.12 - 0.21)	6.42E-14	0.02 (-0.05 - 0.10)	5.94E-01
rs6857	19	45392254	*NECTIN2*	UTR3	T	0.39	0.10	0.17 (0.13 - 0.22)	4.75E-15	0.06 (-0.02 - 0.15)	1.33E-01	0.18 (0.14 - 0.22)	4.20E-16	0.07 (-0.01 - 0.16)	1.08E-01
rs71352238	19	45394336	*TOMM40*	upstream	C	0.33	0.15	0.13 (0.08 - 0.17)	3.09E-08	-0.01 (-0.08 - 0.04)	5.98E-01	0.13 (0.09 - 0.18)	6.45E-09	-0.01 (-0.08 - 0.05)	6.46E-01
rs184017	19	45394969	*TOMM40*	intronic	G	0.41	0.08	0.16 (0.11 - 0.20)	8.21E-13	0.01 (-0.06 - 0.09)	7.37E-01	0.16 (0.12 - 0.21)	7.99E-14	0.01 (-0.06 - 0.09)	6.55E-01
rs2075650	19	45395619	*TOMM40*	intronic	G	0.33	0.37	0.13 (0.08 - 0.17)	1.49E-08	-0.01 (-0.08 - 0.05)	6.61E-01	0.14 (0.09 - 0.18)	2.75E-09	-0.01 (-0.07 - 0.05)	7.28E-01
rs157581	19	45395714	*TOMM40*	exonic	C	0.41	0.08	0.16 (0.11 - 0.20)	6.05E-13	0.01 (-0.06 - 0.09)	7.23E-01	0.16 (0.12 - 0.21)	5.85E-14	0.01 (-0.06 - 0.10)	6.42E-01
rs34095326	19	45395844	*TOMM40*	intronic	A	0.26	0.45	0.12 (0.07 - 0.17)	2.15E-07	0.00 (-0.04 - 0.06)	7.72E-01	0.13 (0.08 - 0.18)	5.68E-08	-0.01 (-0.04 - 0.07)	7.15E-01
rs34404554	19	45395909	*TOMM40*	intronic	G	0.33	0.29	0.13 (0.08 - 0.17)	1.48E-08	-0.01 (-0.08 - 0.05)	6.55E-01	0.14 (0.09 - 0.18)	2.67E-09	-0.01 (-0.07 - 0.05)	7.25E-01
rs11556505	19	45396144	*TOMM40*	exonic	T	0.33	0.29	0.13 (0.08 - 0.17)	1.48E-08	-0.01 (-0.08 - 0.05)	6.55E-01	0.14 (0.09 - 0.18)	2.66E-09	-0.01 (-0.07 - 0.05)	7.25E-01
rs157582	19	45396219	*TOMM40*	intronic	T	0.41	0.12	0.16 (0.11 - 0.20)	3.21E-13	0.01 (-0.06 - 0.10)	6.50E-01	0.17 (0.12 - 0.21)	3.60E-14	0.02 (-0.06 - 0.10)	6.03E-01
rs59007384	19	45396665	*TOMM40*	intronic	T	0.41	0.27	0.16 (0.12 - 0.20)	5.81E-14	0.03 (-0.05 - 0.11)	4.66E-01	0.17 (0.12 - 0.21)	8.34E-15	0.03 (-0.05 - 0.11)	4.72E-01
rs8106922	19	45401666	*TOMM40*	intronic	G	0.30	0.80	-0.11 (-0.15 - -0.06)	6.55E-07	-0.02 (-0.07 - 0.03)	4.36E-01	-0.12 (-0.16 - -0.07)	1.04E-07	-0.02 (-0.07 - 0.02)	3.28E-01
rs34878901	19	45402477	*TOMM40*	intronic	T	0.30	0.74	-0.12 (-0.16 - -0.07)	1.55E-07	-0.02 (-0.07 - 0.02)	3.36E-01	-0.13 (-0.17 - -0.08)	1.83E-08	-0.03 (-0.08 - 0.02)	2.30E-01
rs1160985	19	45403412	*TOMM40*	intronic	T	0.32	0.33	-0.12 (-0.16 - -0.08)	2.51E-08	-0.03 (-0.08 - 0.01)	1.50E-01	-0.13 (-0.17 - -0.09)	2.74E-09	-0.04 (-0.09 - 0.00)	9.28E-02
rs760136	19	45403858	*TOMM40*	intronic	G	0.32	0.33	-0.12 (-0.16 - -0.08)	2.50E-08	-0.03 (-0.08 - 0.01)	1.50E-01	-0.13 (-0.17 - -0.09)	2.73E-09	-0.04 (-0.09 - 0.00)	9.28E-02
rs741780	19	45404431	*TOMM40*	intronic	C	0.32	0.33	-0.12 (-0.16 - -0.08)	2.50E-08	-0.03 (-0.08 - 0.01)	1.50E-01	-0.13 (-0.17 - -0.09)	2.72E-09	-0.04 (-0.09 - 0.00)	9.27E-02
rs1038025	19	45404972	*TOMM40*	intronic	C	0.32	0.33	-0.12 (-0.16 - -0.08)	2.46E-08	-0.03 (-0.08 - 0.01)	1.50E-01	-0.13 (-0.17 - -0.09)	2.67E-09	-0.04 (-0.09 - 0.00)	9.23E-02
rs1038026	19	45405062	*TOMM40*	intronic	G	0.32	0.33	-0.12 (-0.16 - -0.08)	2.53E-08	-0.03 (-0.08 - 0.01)	1.49E-01	-0.13 (-0.17 - -0.09)	2.75E-09	-0.04 (-0.09 - 0.00)	9.17E-02
rs1305062	19	45405521	*TOMM40*	intronic	C	0.30	0.74	-0.12 (-0.16 - -0.07)	7.98E-08	-0.02 (-0.08 - 0.02)	2.88E-01	-0.13 (-0.17 - -0.08)	8.62E-09	-0.03 (-0.08 - 0.01)	1.90E-01
rs10119	19	45406673	*TOMM40*	UTR3	A	0.48	0.25	0.17 (0.12 - 0.21)	5.89E-15	0.06 (-0.00 - 0.13)	7.57E-02	0.17 (0.13 - 0.22)	4.33E-16	0.06 (-0.00 - 0.14)	5.59E-02
rs7259620	19	45407788	*TOMM40*	downstream	A	0.32	0.26	-0.12 (-0.16 - -0.08)	1.89E-08	-0.03 (-0.08 - 0.01)	1.30E-01	-0.13 (-0.18 - -0.09)	1.81E-09	-0.04 (-0.09 - 0.00)	7.58E-02
rs769449	19	45410002	*APOE*	intronic	A	0.33	0.26	0.13 (0.09 - 0.18)	2.94E-09	-0.06 (-0.14 - 0.01)	1.08E-01	0.14 (0.10 - 0.19)	5.61E-10	-0.06 (-0.14 - 0.01)	1.18E-01
rs769450	19	45410444	*APOE*	intronic	A	0.31	0.19	-0.11 (-0.16 - -0.07)	1.32E-07	-0.02 (-0.07 - 0.02)	3.69E-01	-0.13 (-0.17 - -0.08)	1.23E-08	-0.03 (-0.08 - 0.02)	2.38E-01
**rs429358**	**19**	**45411941**	***APOE***	**exonic**	**C**	**0.39**	**0.19**	**0.18 (0.14 - 0.22)**	**1.66E-16**	**0.11 (-0.10 - 0.33)**	**3.14E-01**	**0.18 (0.14 - 0.23)**	**2.07E-17**	**0.10 (-0.12 - 0.32)**	**3.76E-01**
rs75627662	19	45413576	*APOE*	downstream	T	0.35	0.25	0.14 (0.09 - 0.18)	7.39E-10	-0.06 (-0.14 - 0.01)	1.11E-01	0.14 (0.10 - 0.19)	1.14E-10	-0.06 (-0.14 - 0.01)	1.30E-01
rs10414043	19	45415713	*APOC1*	intergenic	A	0.32	0.15	0.14 (0.09 - 0.18)	1.84E-09	-0.05 (-0.13 - 0.02)	1.86E-01	0.14 (0.10 - 0.19)	3.33E-10	-0.05 (-0.13 - 0.02)	2.09E-01
rs7256200	19	45415935	*APOC1*	intergenic	T	0.32	0.11	0.14 (0.09 - 0.18)	1.89E-09	-0.05 (-0.13 - 0.02)	1.84E-01	0.14 (0.10 - 0.19)	3.44E-10	-0.05 (-0.13 - 0.02)	2.05E-01
rs483082	19	45416178	*APOC1*	intergenic	T	0.42	0.28	0.18 (0.14 - 0.22)	1.37E-17	0.14 (-0.04 - 0.32)	1.28E-01	0.19 (0.15 - 0.23)	1.07E-18	0.15 (-0.03 - 0.34)	1.08E-01
rs59325138	19	45416291	*APOC1*	intergenic	T	0.31	0.74	-0.14 (-0.18 - -0.09)	4.06E-10	-0.06 (-0.11 - -0.01)	1.74E-02	-0.14 (-0.19 - -0.10)	1.16E-10	-0.06 (-0.11 - -0.01)	1.78E-02
rs438811	19	45416741	*APOC1*	upstream	T	0.42	0.28	0.18 (0.14 - 0.22)	1.22E-17	0.14 (-0.03 - 0.33)	1.13E-01	0.19 (0.15 - 0.23)	9.43E-19	0.15 (-0.02 - 0.34)	9.49E-02
**rs5117**	**19**	**45418790**	***APOC1***	**intronic**	**C**	**0.41**	**0.27**	**0.18 (0.14 - 0.23)**	**9.42E-18**	**0.15 (-0.01 - 0.32)**	**7.96E-02**	**0.19 (0.15 - 0.23)**	**7.22E-19**	**0.16 (-0.01 - 0.33)**	**6.60E-02**
rs12721046	19	45421254	*APOC1*	intronic	A	0.34	0.00	0.11 (0.07 - 0.16)	5.66E-07	-0.07 (-0.14 - 0.00)	5.30E-02	0.12 (0.07 - 0.17)	2.29E-07	-0.07 (-0.14 - -0.00)	4.54E-02
rs12721056	19	45421744	*APOC1*	intronic	T	0.27	0.24	-0.14 (-0.19 - -0.09)	1.09E-08	-0.06 (-0.11 - -0.00)	2.48E-02	-0.14 (-0.19 - -0.09)	1.37E-08	-0.05 (-0.11 - -0.00)	4.59E-02
rs12721051	19	45422160	*APOC1*	intronic	G	0.41	0.02	0.16 (0.12 - 0.21)	4.15E-13	-0.01 (-0.11 - 0.08)	7.83E-01	0.17 (0.12 - 0.21)	1.58E-13	-0.02 (-0.13 - 0.07)	5.87E-01
rs56131196	19	45422846	*APOC1*	downstream	A	0.41	0.02	0.16 (0.12 - 0.21)	4.22E-13	-0.01 (-0.11 - 0.08)	7.93E-01	0.17 (0.12 - 0.21)	1.59E-13	-0.02 (-0.13 - 0.07)	5.98E-01
rs4420638	19	45422946	*APOC1*	downstream	G	0.41	0.02	0.16 (0.12 - 0.21)	5.94E-13	-0.01 (-0.11 - 0.08)	7.40E-01	0.17 (0.12 - 0.21)	2.37E-13	-0.03 (-0.13 - 0.07)	5.42E-01
rs78959900	19	45423636	*APOC1*	intergenic	A	0.26	0.38	-0.14 (-0.19 - -0.09)	2.57E-08	-0.05 (-0.11 - -0.00)	3.33E-02	-0.14 (-0.19 - -0.09)	4.38E-08	-0.05 (-0.10 - 0.00)	6.80E-02
rs111789331	19	45427125	*APOC1P1*	intergenic	A	0.34	0.01	0.12 (0.07 - 0.17)	2.12E-07	-0.06 (-0.13 - 0.01)	1.00E-01	0.13 (0.08 - 0.17)	8.26E-08	-0.06 (-0.13 - 0.00)	8.85E-02
rs4803770	19	45427353	*APOC1P1*	intergenic	G	0.29	0.41	-0.14 (-0.18 - -0.09)	8.91E-09	-0.05 (-0.11 - -0.00)	2.95E-02	-0.14 (-0.19 - -0.09)	5.43E-09	-0.05 (-0.10 - -0.00)	3.92E-02
rs66626994	19	45428234	*APOC1P1*	intergenic	A	0.34	0.01	0.12 (0.07 - 0.17)	3.09E-07	-0.06 (-0.13 - 0.00)	8.55E-02	0.13 (0.08 - 0.17)	1.25E-07	-0.06 (-0.13 - 0.00)	7.46E-02
rs4803772	19	45428459	*APOC1P1*	intergenic	T	0.26	0.17	-0.14 (-0.19 - -0.09)	2.27E-08	-0.06 (-0.11 - -0.00)	2.34E-02	-0.14 (-0.19 - -0.09)	2.76E-08	-0.05 (-0.11 - -0.00)	4.18E-02
**rs35136575**	**19**	**45439163**	***APOC1P1***	**intergenic**	**G**	**0.24**	**0.81**	**0.13 (0.08 - 0.18)**	**8.61E-07**	**0.13 (0.08 - 0.18)**	**3.24E-07**	**0.12 (0.07 - 0.18)**	**2.57E-06**	**0.12 (0.07 - 0.18)**	**1.09E-06**
rs73045691	19	45440529	*APOC4*	intergenic	A	0.32	0.48	0.11 (0.07 - 0.16)	4.21E-07	0.08 (0.04 - 0.13)	1.42E-04	0.12 (0.07 - 0.17)	2.10E-07	0.09 (0.04 - 0.13)	1.04E-04
rs34041051	19	45442349	*APOC4*	intergenic	C	0.33	0.67	0.11 (0.07 - 0.16)	3.12E-07	0.08 (0.04 - 0.13)	1.11E-04	0.12 (0.07 - 0.17)	1.55E-07	0.09 (0.04 - 0.13)	8.02E-05
rs35336243	19	45442519	*APOC4*	intergenic	T	0.33	0.67	0.11 (0.07 - 0.16)	3.12E-07	0.08 (0.04 - 0.13)	1.10E-04	0.12 (0.07 - 0.17)	1.55E-07	0.09 (0.04 - 0.13)	8.00E-05
rs73045696	19	45443088	*APOC4*	intergenic	C	0.33	0.67	0.11 (0.07 - 0.16)	3.98E-07	0.08 (0.04 - 0.13)	1.34E-04	0.12 (0.07 - 0.16)	2.10E-07	0.08 (0.04 - 0.13)	1.01E-04
rs59859410	19	45443504	*APOC4*	intergenic	G	0.33	0.67	0.12 (0.07 - 0.16)	2.53E-07	0.08 (0.04 - 0.13)	8.36E-05	0.12 (0.07 - 0.17)	1.21E-07	0.09 (0.04 - 0.13)	5.84E-05
rs112784534	19	45444566	*APOC4*	upstream	T	0.33	0.87	0.12 (0.07 - 0.16)	2.23E-07	0.08 (0.04 - 0.13)	7.32E-05	0.12 (0.07 - 0.17)	1.09E-07	0.09 (0.04 - 0.13)	5.19E-05
rs5167	19	45448465	*APOC4*	exonic	G	0.37	1.00	0.10 (0.06 - 0.15)	8.66E-07	0.07 (0.03 - 0.11)	3.72E-04	0.11 (0.07 - 0.15)	3.30E-07	0.08 (0.03 - 0.12)	2.28E-04
rs4803791	19	45523583	*RELB*	intronic	A	0.25	0.84	0.12 (0.07 - 0.17)	6.68E-07	0.09 (0.04 - 0.13)	1.75E-04	0.12 (0.07 - 0.17)	5.78E-07	0.09 (0.04 - 0.13)	2.52E-04

Table S5: Variants associated with CAA at p< 1E-06 on Chr19 cannot be entirely explained by the *APOE*ε4 risk allele. Association results for SNPs on Chr19 with a p-value <1E-05 in the primary model (adjusted for Braak and Thal, without *APOE*) are provided across four models tested. **Bolded** rows represent the most significant SNP (**rs5117**), the *APOE*ε4 tagging SNP (**rs429358**) and the top SNP (r**s35136575**) that remains significant after adjustment for *APOE*ε2 and ε4 alleles. "All" indicates that the full cohort (N=821) was tested, "Braak", "Thal", and "APOE" adj are indicated when included in the model as covariates; Age, Sex, batch and the first three population principal components PC's were included in all models. Full set of results for the primary model can be found on the AMP-AD knowledge portal (**Table S2**).

SNP	CHR	POS (hg19)	Closet Gene	Function	Tested allele	MAF	HWE-P	All, Braak, Thal adj		All, Braak, Thal, APOE adj		All		All, APOE adj	
								Beta (95%CI)	p-value	Beta (95%CI)	p-value	Beta (95%CI)	p-value	Beta (95%CI)	p-value
rs12142431	1	61398276	*NFIA-AS2*	intergenic	C	0.06	0.73	-0.20 (-0.29, -0.11)	5.51E-06	-0.18 (-0.26, -0.09)	2.99E-05	-0.19 (-0.28, -0.10)	2.34E-05	-0.17 (-0.25, -0.08)	8.93E-05
rs12138551	1	61398591	*NFIA-AS2*	intergenic	A	0.06	0.73	-0.20 (-0.29, -0.11)	6.02E-06	-0.18 (-0.26, -0.09)	3.23E-05	-0.19 (-0.28, -0.10)	2.57E-05	-0.17 (-0.25, -0.08)	9.65E-05
rs17303886	1	61400587	*NFIA-AS2*	intergenic	C	0.06	0.73	-0.20 (-0.29, -0.11)	6.29E-06	-0.18 (-0.26, -0.09)	3.30E-05	-0.19 (-0.28, -0.10)	3.14E-05	-0.17 (-0.25, -0.08)	1.13E-04
rs185818224	2	107942483	*MIR548AU*	ncRNA_intronic	A	0.02	1.00	0.37 (0.21, 0.53)	4.05E-06	0.34 (0.19, 0.50)	8.38E-06	0.35 (0.18, 0.51)	2.22E-05	0.32 (0.17, 0.47)	4.07E-05
rs113984219	4	3931801	*FAM86EP*	intergenic	C	0.05	0.16	-0.26 (-0.38, -0.15)	4.96E-06	-0.25 (-0.36, -0.14)	4.76E-06	-0.27 (-0.39, -0.16)	3.15E-06	-0.26 (-0.37, -0.15)	3.85E-06
rs74463826	6	150461804	*PPP1R14C*	intergenic	T	0.07	0.58	-0.18 (-0.26, -0.10)	5.98E-06	-0.16 (-0.24, -0.09)	2.01E-05	-0.17 (-0.25, -0.09)	3.36E-05	-0.15 (-0.23, -0.07)	9.15E-05
rs76498200	6	150466373	*PPP1R14C*	intronic	G	0.07	0.58	-0.18 (-0.26, -0.10)	9.26E-06	-0.16 (-0.24, -0.08)	3.28E-05	-0.17 (-0.25, -0.08)	4.90E-05	-0.15 (-0.23, -0.07)	1.44E-04
rs13226070	7	2682232	*TTYH3*	intronic	C	0.23	0.31	-0.12 (-0.17, -0.06)	7.51E-06	-0.11 (-0.16, -0.06)	8.76E-06	-0.12 (-0.17, -0.06)	7.87E-06	-0.11 (-0.16, -0.06)	1.02E-05
rs118189570	7	100858604	*PLOD3*	intronic	C	0.02	1.00	0.32 (0.18, 0.47)	5.96E-06	0.27 (0.14, 0.41)	6.96E-05	0.33 (0.18, 0.47)	6.66E-06	0.28 (0.14, 0.42)	6.74E-05
rs2471279	7	139937237	*JHDM1D-AS1*	intergenic	A	0.27	0.08	0.10 (0.06, 0.15)	5.05E-06	0.10 (0.06, 0.14)	3.06E-06	0.10 (0.05, 0.15)	8.42E-06	0.10 (0.05, 0.14)	4.43E-06
rs1503365	8	68435188	*CPA6*	intronic	G	0.36	0.55	0.10 (0.05, 0.14)	6.28E-06	0.09 (0.05, 0.13)	1.34E-05	0.09 (0.05, 0.14)	1.35E-05	0.08 (0.04, 0.13)	3.57E-05
rs1503366	8	68435226	*CPA6*	intronic	C	0.36	0.55	0.10 (0.05, 0.14)	6.27E-06	0.09 (0.05, 0.13)	1.34E-05	0.09 (0.05, 0.14)	1.35E-05	0.08 (0.04, 0.13)	3.57E-05
rs17429185	8	68436385	*CPA6*	intronic	T	0.36	0.55	0.10 (0.05, 0.14)	6.44E-06	0.09 (0.05, 0.13)	1.34E-05	0.09 (0.05, 0.14)	1.37E-05	0.08 (0.04, 0.13)	3.54E-05
rs13271535	8	68436405	*CPA6*	intronic	A	0.36	0.55	0.10 (0.05, 0.14)	6.46E-06	0.09 (0.05, 0.13)	1.34E-05	0.09 (0.05, 0.14)	1.37E-05	0.08 (0.04, 0.13)	3.54E-05
rs34860297	8	68436547	*CPA6*	intronic	C	0.36	0.60	0.10 (0.05, 0.14)	6.88E-06	0.09 (0.05, 0.13)	1.35E-05	0.09 (0.05, 0.14)	1.43E-05	0.08 (0.04, 0.13)	3.52E-05
rs1682076	8	68524513	*CPA6*	intronic	T	0.17	0.21	0.13 (0.07, 0.18)	5.44E-06	0.11 (0.06, 0.17)	2.37E-05	0.12 (0.07, 0.18)	1.22E-05	0.11 (0.06, 0.17)	4.57E-05
rs6580887	12	52967080	*KRT74*	intronic	G	0.17	0.39	-0.12 (-0.18, -0.07)	8.02E-06	-0.11 (-0.16, -0.06)	3.15E-05	-0.12 (-0.18, -0.07)	1.23E-05	-0.11 (-0.16, -0.05)	5.49E-05
rs7302968	12	53004603	*KRT73*	exonic	A	0.17	0.81	-0.12 (-0.18, -0.07)	8.96E-06	-0.11 (-0.16, -0.05)	4.92E-05	-0.12 (-0.17, -0.06)	1.74E-05	-0.10 (-0.16, -0.05)	1.01E-04
rs61521894	12	53007046	*KRT73-AS1*	ncRNA_intronic	T	0.18	0.90	-0.12 (-0.17, -0.07)	7.60E-06	-0.10 (-0.16, -0.05)	4.49E-05	-0.12 (-0.17, -0.06)	1.56E-05	-0.10 (-0.15, -0.05)	9.68E-05
rs75026370	12	97349366	*NEDD1*	intergenic	A	0.03	0.29	0.29 (0.16, 0.42)	5.88E-06	0.26 (0.13, 0.38)	3.20E-05	0.27 (0.14, 0.40)	2.69E-05	0.23 (0.11, 0.36)	1.52E-04
rs148966106	12	97487004	*NEDD1*	intergenic	T	0.03	0.37	0.28 (0.16, 0.40)	7.07E-06	0.25 (0.13, 0.37)	2.42E-05	0.26 (0.14, 0.39)	3.18E-05	0.23 (0.11, 0.35)	1.02E-04
rs2650167	12	117967072	*KSR2*	intronic	A	0.48	1.00	-0.09 (-0.13, -0.05)	5.91E-06	-0.09 (-0.13, -0.05)	8.03E-06	-0.09 (-0.14, -0.05)	6.37E-06	-0.09 (-0.13, -0.05)	8.97E-06
rs7161937	15	90853933	*NGRN*	intergenic	G	0.18	0.66	-0.14 (-0.20, -0.08)	2.99E-06	-0.13 (-0.18, -0.07)	6.98E-06	-0.14 (-0.20, -0.08)	5.61E-06	-0.12 (-0.18, -0.07)	1.56E-05
rs1431441	20	13026863	*SPTLC3*	intronic	T	0.32	0.87	-0.10 (-0.14, -0.05)	8.92E-06	-0.08 (-0.12, -0.04)	8.25E-05	-0.09 (-0.14, -0.05)	1.74E-05	-0.08 (-0.12, -0.04)	1.16E-04
rs3789353	20	13029437	*SPTLC3*	intronic	A	0.32	0.87	-0.10 (-0.14, -0.05)	6.55E-06	-0.08 (-0.13, -0.04)	5.93E-05	-0.10 (-0.14, -0.05)	1.38E-05	-0.08 (-0.13, -0.04)	8.80E-05
rs2327682	20	13034005	*SPTLC3*	intronic	A	0.32	0.87	-0.10 (-0.14, -0.05)	6.56E-06	-0.08 (-0.13, -0.04)	5.94E-05	-0.10 (-0.14, -0.05)	1.38E-05	-0.08 (-0.13, -0.04)	8.81E-05
rs4814191	20	13034010	*SPTLC3*	intronic	G	0.32	0.87	-0.10 (-0.14, -0.05)	6.56E-06	-0.08 (-0.13, -0.04)	5.94E-05	-0.10 (-0.14, -0.05)	1.38E-05	-0.08 (-0.13, -0.04)	8.81E-05
rs6078905	20	13034705	*SPTLC3*	intronic	C	0.32	0.87	-0.10 (-0.14, -0.05)	6.56E-06	-0.08 (-0.13, -0.04)	5.93E-05	-0.10 (-0.14, -0.05)	1.38E-05	-0.08 (-0.13, -0.04)	8.80E-05

Table S6. Variants associated with CAA at p< 1E-05 in the primary model are largely consistent across analysis models regardless of AD neuropathology or *APOE*ε2, ε4 adjustment. Association results are shown for SNPs with a p-value <1E-05 in the primary model (adjusted for Braak and Thal, without *APOE*), across four models tested. Additional abbreviations and explanation as in **Table S5**. Note: *NGRN* = Neugrin, not to be confused with Neurogranin (*NRGN*).

Analysis	Pathway ID[a]	Pathway Name	Size	Count[b]	Z-score	p-value	q-value	Top ranked genes[c]
Primary Model	GO:2000463	positive regulation of excitatory postsynaptic potential	19	18	4.84	6.45E-07	2.08E-03	*SHANK1; DRD4; GRIN1; NLGN1; STX1B; SHANK3; NETO1*
Primary Model	GO:0050770	regulation of axonogenesis	165	158	4.10	2.03E-05	1.19E-02	*APOE; POU3F2; WNT5A; GRIN1; CACNA1A; SEMA4B; TWF2; SLIT3; LPAR3; RTN4; SHOX2; NRG1; SEMA3B; LRRC4C; SEMA6B; NGF; EFNA5; PLXND1; ROBO1; ROBO2; ARHGDIA; SSH3; BDNF*
Primary Model	GO:0035176	social behaviour	45	42	3.97	3.58E-05	1.63E-02	*SHANK1; VPS13A; MKKS; DRD4; GRIN1; SHANK2; SHANK3*
Primary Model	GO:1901068	guanosine-containing compound metabolism	41	35	3.73	9.46E-05	2.29E-02	*AK3; NME2; NME1-NME2; TJP2; IMPDH1; AMPD2; RHOQ*
Primary Model	GO:0042178	xenobiotic catabolic process	10	10	3.53	2.06E-04	2.71E-02	*ACSL1; PON3; CRYZ; UGT1A1*
Primary Model	GO:0002931	response to ischemia	26	25	3.43	2.98E-04	3.25E-02	*CIB1; CPEB4; FAM175B; MAP2K6; PPIF; HYOU1; FAIM2*
Primary Model	GO:0097479	synaptic vesicle localization	84	79	3.25	5.70E-04	4.31E-02	*DENND1A; PIP5K1C; NLGN1; STX4; STX1B; SEPT5; BLOC1S5; AP3B2*
APOE ε4 interaction	GO:0072210	metanephric nephron development	33	33	4.05	2.61E-05	4.20E-02	*SALL1; GDNF; IRX1; LAMB2; PKD1; PDGFRB; TFAP2B*
Sex interaction	GO:0002042	cell migration involved in sprouting angiogenesis	15	15	4.10	2.10E-05	2.39E-02	*NR4A1; ITGB1; SLIT2; ROBO1; EPHB4; EGR3*
Sex interaction	GO:0050905	neuromuscular process	110	108	5.23	8.67E-08	5.59E-04	*RBFOX1; CSMD1; POU4F1; GBX1; DRD2; PDE8B; TNR; DRD1; USH1G; SCN1A; FGF14; ATP8A2; KCNMA1; DRD3; ADCY5; CDH23; PARK2; SHANK1; SLC6A3; CACNA1A; RBFOX2*
Sex interaction	GO:0031295	T cell costimulation	77	68	4.47	3.84E-06	1.24E-02	*CD3D; CD3G; GRAP2; CD247; TNFSF14; CD3E; TMIGD2; HLA-DPB1; HLA-DPA1; VAV1; HLA-DRB5; CD80; CDC42; TNFSF4*

Table S7: Lead gene ontology biological processes enriched for genes associated with CAA for three models. Gene set enrichment analysis results from GSA-SNP2 using gene-ontology (2017) as the background pathway reference. Primary model refers to analysis results from the model testing SNPs for association with CAA while adjusting for Age, Sex, batch, PC1-3, Braak and Thal. *APOE*ε4 interaction model is the same as the primary model with the addition of *APOE*ε4 and an *APOE*ε4 *SNP interaction term. Sex interaction model is the same as the primary model with the addition of a sex*SNP interaction term. a = Gene Ontology pathway ID; b = the number of pathway genes with at least one variant present in the GWAS results file; c = list of genes with a gene score ≥ 1.

SNP	CHR	POS (hg19)	Closest Gene	Function	Tested Allele	MAF	HWE p-value	Sex-Int p-value	Females Beta (95% CI)	Females p-value	Males Beta (95% CI)	Males p-value
rs6729667	2	10489582	*HPCAL1*	intronic	G	0.21	0.27	9.84E-08	0.14 (0.07 - 0.21)	1.58E-04	-0.15 (-0.22 - -0.07)	2.61E-04
rs117114154	8	123526558	*SMILR*	ncRNA_intronic	G	0.06	0.20	1.93E-07	0.26 (0.14 - 0.38)	1.37E-05	-0.19 (-0.32 - -0.07)	3.14E-03
rs77377847	3	19659617	*KCNH8*	intergenic	A	0.03	1.00	1.16E-06	-0.25 (-0.4 - -0.10)	1.16E-03	0.36 (0.17 - 0.54)	1.81E-04
rs139792624	5	164425094	*LOC102546299*	intergenic	G	0.03	1.00	1.32E-06	0.34 (0.16 - 0.52)	2.85E-04	-0.38 (-0.59 - -0.17)	4.85E-04
rs994754	15	97559159	*SPATA8*	intergenic	T	0.10	1.00	2.74E-06	-0.16 (-0.25 - -0.06)	1.02E-03	0.18 (0.07 - 0.29)	1.39E-03
rs1789892	4	100250658	*ADH1C*	intergenic	A	0.40	0.94	4.23E-06	0.11 (0.06 - 0.17)	6.85E-05	-0.09 (-0.16 - -0.02)	8.67E-03
rs76546671	2	155379552	*LOC100144595*	intergenic	G	0.03	1.00	5.41E-06	-0.33 (-0.53 - -0.14)	8.18E-04	0.31 (0.11 - 0.51)	2.70E-03
rs13382310	2	20736364	*HS1BP3-IT1*	intergenic	T	0.26	0.58	6.37E-06	-0.11 (-0.17 - -0.04)	1.50E-03	0.11 (0.04 - 0.18)	2.94E-03
rs147787000	11	107480790	*ELMOD1*	intronic	A	0.03	1.00	6.77E-06	0.47 (0.26 - 0.67)	1.40E-05	-0.18 (-0.37 - 0.02)	7.85E-02
rs77733101	13	51807788	*FAM124A*	intronic	T	0.19	0.18	7.13E-06	0.11 (0.04 - 0.18)	3.17E-03	-0.15 (-0.23 - -0.07)	2.99E-04
rs72762782	16	4072015	*ADCY9*	intronic	G	0.13	0.88	8.50E-06	-0.12 (-0.21 - -0.03)	6.53E-03	0.17 (0.08 - 0.26)	1.91E-04
rs78786429	8	126784103	*LINC00861*	intergenic	A	0.08	0.52	8.81E-06	0.16 (0.04 - 0.28)	7.01E-03	-0.21 (-0.34 - -0.09)	7.52E-04
rs12475694	2	190772608	*PMS1*	intergenic	T	0.28	1.00	8.90E-06	-0.08 (-0.15 - -0.02)	8.59E-03	0.13 (0.06 - 0.2)	2.10E-04
rs72823866	17	36669125	*ARHGAP23*	downstream	G	0.02	1.00	9.90E-06	0.22 (0.04 - 0.39)	1.40E-02	-0.42 (-0.64 - -0.2)	2.30E-04
rs145151025	5	164303176	*LINC01947*	intergenic	A	0.03	1.00	9.94E-06	0.27 (0.1 - 0.45)	1.91E-03	-0.35 (-0.55 - -0.15)	8.04E-04

Table S8. Variants associated with CAA at p< 1E-05 in the sex interaction analysis model. Association results are shown for the most significant SNP per locus, with a p-value < 1E-05 in the sex interaction model (adjusted for Braak and Thal). Results for these SNPs in the sex stratified analysis are also provided. Abbreviations as in **Table S5**.

SNP	CHR	POS (hg19)	*Closest Gene*	Function	Tested Allele	MAF	HWE p-value	APOE-Int p-value	APOE-Positive beta (95%CI)	APOE-Positive p-value	APOE-Negative beta (95%CI)	APOE-Negative p-value
rs10234094	7	130691759	*LINC-PINT*	ncRNA_intronic	C	0.09	0.51	1.07E-07	0.04 (-0.04 - 0.13)	3.02E-01	-0.37 (-0.49 - -0.24)	1.63E-08
rs71619047	4	181945567	*LINC00290*	intergenic	A	0.04	0.63	2.59E-07	0.24 (0.10 - 0.38)	7.42E-04	-0.33 (-0.50 - -0.16)	1.59E-04
rs13207159	6	150421688	*ULBP3*	intergenic	A	0.25	0.71	5.31E-07	-0.05 (-0.10 - 0.00)	7.68E-02	0.19 (0.11 - 0.28)	3.64E-06
rs77731990	2	184556860	*NUP35*	intergenic	G	0.08	0.31	7.23E-07	-0.14 (-0.23 - -0.05)	1.47E-03	0.28 (0.13 - 0.42)	2.61E-04
rs58143459	4	101223313	*LOC101929353*	intergenic	C	0.19	1.00	7.27E-07	-0.07 (-0.13 - -0.01)	1.89E-02	0.23 (0.13 - 0.33)	4.45E-06
rs8059669	16	68845211	*CDH1*	intronic	C	0.14	0.87	1.03E-06	-0.12 (-0.20 - -0.04)	1.37E-03	0.18 (0.08 - 0.29)	4.26E-04
rs7604021	2	225636059	*DOCK10*	intronic	A	0.41	0.51	1.37E-06	0.08 (0.03 - 0.13)	4.38E-04	-0.13 (-0.21 - -0.05)	4.93E-04
rs11694957	2	230419057	*DNER*	intronic	T	0.33	0.07	1.89E-06	-0.04 (-0.10 - 0.00)	9.88E-02	0.18 (0.10 - 0.26)	6.32E-06
rs1955	15	51238095	*DCAF13P3*	ncRNA_exonic	A	0.24	0.92	2.07E-06	0.09 (0.03 - 0.14)	1.53E-03	-0.16 (-0.25 - -0.07)	4.69E-04
rs764589	16	66183383	*CDH5*	intergenic	T	0.09	0.60	2.22E-06	-0.15 (-0.25 - -0.06)	5.95E-04	0.23 (0.08 - 0.37)	1.80E-03
rs116915314	6	98809185	*MIR2113*	intergenic	G	0.03	0.51	2.64E-06	-0.20 (-0.34 - -0.06)	3.50E-03	0.42 (0.19 - 0.65)	4.01E-04
rs28562159	8	133021149	*EFR3A*	intronic	A	0.31	0.57	2.73E-06	-0.05 (-0.11 - -0.00)	3.45E-02	0.16 (0.08 - 0.23)	3.57E-05
rs4415069	5	112866908	*YTHDC2*	intronic	T	0.47	0.67	3.39E-06	-0.09 (-0.14 - -0.04)	2.22E-04	0.12 (0.04 - 0.19)	1.22E-03
rs149890934	2	72183011	*CYP26B1*	intergenic	T	0.03	1.00	3.86E-06	0.16 (0.01 - 0.32)	3.29E-02	-0.51 (-0.76 - -0.26)	5.39E-05
rs9950268	18	4925256	*LINC01892*	intergenic	G	0.42	0.17	3.92E-06	-0.10 (-0.16 - -0.05)	3.17E-05	0.08 (0.01 - 0.16)	2.65E-02
rs62166368	2	34678735	*LINC01317*	intergenic	G	0.07	0.59	4.63E-06	-0.14 (-0.24 - -0.04)	5.94E-03	0.22 (0.09 - 0.36)	7.68E-04
rs13425074	2	7188585	*RNF144A*	intronic	T	0.02	1.00	5.00E-06	-0.17 (-0.34 - -0.00)	5.01E-02	0.51 (0.26 - 0.77)	8.45E-05
rs4380195	2	16013590	*MYCNUT*	intergenic	A	0.30	0.28	5.24E-06	0.02 (-0.03 - 0.07)	4.44E-01	-0.18 (-0.25 - -0.11)	1.35E-06
rs27047	5	1412251	*SLC6A3*	intronic	C	0.24	0.45	5.47E-06	-0.08 (-0.14 - -0.02)	4.96E-03	0.15 (0.06 - 0.23)	5.55E-04
rs7125147	11	110703407	*ARHGAP20*	intergenic	T	0.27	0.48	6.56E-06	0.07 (0.02 - 0.13)	6.41E-03	-0.14 (-0.22 - -0.06)	5.35E-04
rs2852772	18	42262869	*SETBP1*	intronic	A	0.17	0.90	7.64E-06	-0.10 (-0.17 - -0.04)	9.61E-04	0.15 (0.05 - 0.25)	2.63E-03
rs6517101	21	33868483	*EVA1C*	intronic	G	0.34	0.43	8.02E-06	-0.09 (-0.14 - -0.03)	7.72E-04	0.11 (0.03 - 0.19)	5.57E-03
rs73932448	2	60117115	*MIR4432HG*	intergenic	A	0.04	1.00	8.87E-06	-0.16 (-0.29 - -0.03)	1.66E-02	0.36 (0.16 - 0.56)	4.88E-04
rs12513273	4	163242929	*FSTL5*	intergenic	A	0.04	0.64	9.27E-06	0.13 (0.01 - 0.25)	2.69E-02	-0.37 (-0.56 - -0.17)	2.76E-04
rs117101753	6	103322776	*GRIK2*	intergenic	A	0.03	0.39	9.75E-06	0.37 (0.20 - 0.54)	1.88E-05	-0.22 (-0.43 - -0.01)	3.46E-02

Table S9. Variants associated with CAA at p< 1E-05 in the *APOE*ε4 interaction analysis model. Association results are shown for the most significant SNP per locus, with a p-value <1E-05 in the *APOE*ε4 interaction model (adjusted for Braak and Thal). Results for these SNPs in the *APOE* stratified analysis are also provided. The most significant SNP at the *LINC-PINT* locus is genome wide significant in the *APOE*-negative subset. Abbreviations as for **Table S5**.

Dataset	Data type	Brain region	AMP-AD Knowledge Portal ID[a]	N	N: Diagnosis AD	Control	PSP	PA	N: Females (%)	N: *APOE* ε4 dose (%) 0	1	2	Mean Age at Death (SD)	Mean RIN (SD)
Mayo Clinic	RNAseq + WGS	CER	syn5550404	244	79	65	78	22	120 (49%)	175 (72%)	63 (26%)	6 (2%)	80.4 (9.1)	8.2 (0.9)
Mayo Clinic	RNAseq + WGS	TCX	syn5550404	257	79	67	81	30	126 (49%)	185 (72%)	64 (25%)	8 (3%)	80.7 (9.0)	8.2 (0.9)

Table S10. Characteristics of Mayo Clinic brain eQTL dataset. Gene expression measures collected from two brain regions (CER = Cerebellum, TCX = Temporal Cortex) using RNA sequencing on from individuals that likewise had available whole genome sequence (WGS) data. AD = Alzheimer's disease, PSP = Progressive supranuclear palsy, PA = pathologic aging, SD = standard deviation, a: Synapse Ids provided for datasets can be searched for on the AMP-AD knowledge portal https://adknowledgeportal.synapse.org

SNP	CHR	POS	REF	ALT	MAF	HWE-P	R2	D'	CADD13 PHRED	Regulome score	TCX: *LINC-PINT* Beta (se)	TCX: *LINC-PINT* P	CER: *LINC-PINT* Beta (se)	CER: *LINC-PINT* P	eQTL TCX Gene	eQTL TCX Beta	eQTL TCX P	eQTL-CER Gene	eQTL-CER Beta	eQTL-CER P
**rs10234094**	**7**	**130691759**	**T**	**C**	**0.10**	**0.28**	**1.00**	**1.00**	**7.67**	**3a**	**0.02 (0.03)**	**6.10E-01**	**0.11 (0.06)**	**9.52E-02**	***TMEM209***	**-0.12**	**1.97E-02**	**ns**	**ns**	**ns**
rs10227122	7	130693683	C	G	0.19	1.00	0.48	0.98	1.54	4	0.02 (0.03)	4.10E-01	0.09 (0.05)	8.89E-02	ns	ns	ns	ns	ns	ns
rs1588770	7	130693823	A	G	0.11	0.28	0.93	0.98	3.77	4	0.02 (0.03)	5.49E-01	0.12 (0.06)	6.14E-02	*TMEM209*	-0.12	1.09E-02	*CEP41*	-0.12	3.52E-02
rs10256380	7	130693931	A	G	0.11	0.28	0.93	0.98	6.21	2b	0.02 (0.03)	5.49E-01	0.12 (0.06)	6.14E-02	*TMEM209*	-0.12	1.09E-02	*CEP41*	-0.12	3.52E-02
rs115718933	7	130694165	G	A	0.11	0.28	0.91	0.98	0.96	2a	0.02 (0.03)	5.49E-01	0.12 (0.06)	6.14E-02	*TMEM209*	-0.12	1.09E-02	*CEP41*	-0.12	3.52E-02
rs1588769	7	130696720	T	C	0.20	0.76	0.47	1.00	3.20	5	0.02 (0.03)	3.94E-01	0.08 (0.05)	1.35E-01	ns	ns	ns	ns	ns	ns
rs112785937	7	130696890	A	C	0.11	0.09	0.92	1.00	0.03	2b	0.02 (0.03)	5.60E-01	0.12 (0.06)	5.69E-02	*TMEM209*	-0.12	1.37E-02	*CEP41*	-0.13	2.34E-02
rs10668568	7	130699914	GTT	G	0.33	0.49	0.22	0.97	15.94	3a	-0.03 (0.02)	2.61E-01	0.07 (0.05)	1.59E-01	ns	ns	ns	ns	ns	ns
rs765965	7	130704375	A	C	0.15	1.00	0.64	0.98	0.28	4	-0.03 (0.03)	3.81E-01	0.02 (0.05)	6.59E-01	*KLHDC10*	0.06	1.12E-02	ns	ns	ns
rs12234702	7	130709113	C	T	0.10	0.24	0.78	0.90	2.98	7	0.02 (0.03)	6.62E-01	0.13 (0.07)	4.90E-02	*TMEM209*	-0.15	3.78E-03	*CEP41*	-0.14	2.91E-02

Table S11: Fine-mapping and annotation of *LINC-PINT* locus variants. Variants in linkage disequilibrium (D' > 0.8, r2 > 0.2) with the lead SNP **rs10234094,** and with a MAF > 2%, were identified using whole genome sequencing data from the "Mayo Clinic RNAseq study" available in the AMP-AD knowledge portal (**Table S2**). CADD (Combined Annotation Dependent Depletion) score indicates deleteriousness of variants where higher score indicates increased predicted pathogenicity[9]. Regulome score provides predicted regulatory annotation where lower score indicates higher regulatory functional potential[8]. Association with brain gene expression levels of *LINC-PINT* are provided for all variants. Association with brain gene expression levels of other proximal genes (+/-1Mb) are shown when nominally significant (p<0.05). TCX = temporal cortex, CER = cerebellum.

Dataset	Brain region	AMP-AD Knowledge Portal ID[a]	Data type	AMP-AD Knowledge Portal ID[a]	Diagnosis	N	N: Females (%)	N: *APOE* ε4 dose (%)				Mean Age at Death (SD)	Mean RIN (SD)
								0	1	2	NA		
Mayo Clinic	CER	syn5550404	RNAseq	syn8690904	AD	79	47 (59%)	38 (48%)	36 (46%)	5 (6%)	0 (0%)	82.5 (7.7)	8.4 (0.7)
					Con	65	28 (43%)	56 (86%)	9 (14%)	0 (0%)	0 (0%)	82.0 (8.6)	7.8 (0.9)
Mayo Clinic	TCX	syn5550404	RNAseq	syn8690799	AD	80	49 (61%)	38 (48%)	35 (44%)	7 (8%)	0 (0%)	82.6 (7.7)	8.6 (0.6)
					Con	68	34 (50%)	60 (88%)	8 (12%)	0 (0%)	0 (0%)	82.2 (8.5)	7.8 (1.0)
ROSMAP	DLFPC	syn3219045	RNAseq	syn8691134	AD	295	206 (70%)	187 (63%)	104 (35%)	3 (1%)	1 (0.3%)	88.0 (3.2)	7.0 (0.9)
					Con	180	100 (56%)	162 (90%)	17 (9.4%)	1 (0.6%)	0 (0%)	84.8 (5.6)	7.2 (1.0)
MSBB	BM10	syn3159438	RNAseq	syn8691099	AD	80	57 (71%)	32 (40%)	21 (26%)	2 (2.5%)	25 (31%)	85.3 (6.0)	6.7 (0.8)
					Con	44	23 (52%)	25 (57%)	4 (9%)	0 (0%)	15 (34%)	82.7 (8.0)	6.8 (0.8)
MSBB	BM22	syn3159438	RNAseq	syn8691099	AD	70	49 (70%)	28 (40%)	15 (21%)	1 (1.4%)	26 (37%)	84.0 (6.9)	6.3 (1.0)
					Con	33	18 (55%)	17 (52%)	2 (6.1%)	0 (0%)	14 (42%)	82.8 (8.3)	6.3 (0.8)
MSBB	BM36	syn3159438	RNAseq	syn8691099	AD	56	41 (73%)	24 (43%)	8 (14%)	1 (2%)	23 (41%)	85.6 (6.1)	6.6 (1.0)
					Con	30	14 (47%)	18 (60%)	2 (7%)	0 (0%)	10 (33%)	82.2 (9.2)	6.8 (0.9)
MSBB	BM44	syn3159438	RNAseq	syn8691099	AD	73	50 (69%)	31 (42%)	13 (18%)	2 (3%)	27 (37%)	85.2 (6.2)	7.9 (1.8)
					Con	33	17 (52%)	18 (55%)	3 (9%)	0 (0%)	12 (36%)	82.4 (8.4)	8.5 (1.7)

Table S12. Characteristics of AMP-AD gene expression datasets. Gene expression measures collected from six brain regions (CER = Cerebellum, TCX = Temporal Cortex (aka BM22), DLPFC = dorsolateral prefrontal cortex, BM = Brodman area) using RNA sequencing. AD = Alzheimer's disease, SD = standard deviation. **a:** Synapse IDs provided for datasets can be searched for on the AMP-AD knowledge portal https://adknowledgeportal.synapse.org

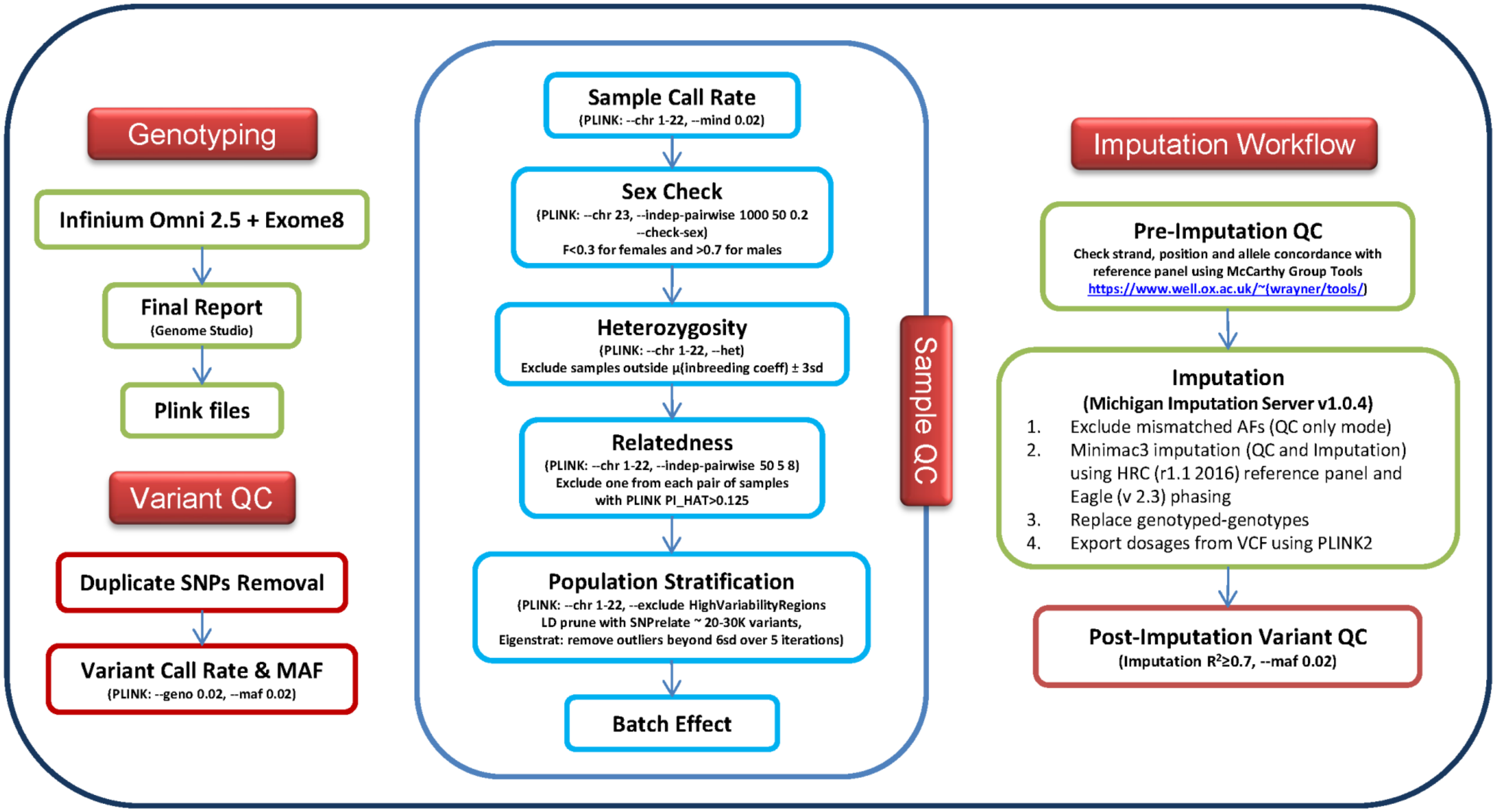

Figure S1. Genome wide genotyping was performed using the Infinium Omni 2.5+Exome 8 v1.3 (cohort A) or v1.4 (cohort B). Genotypes were exported to comma separated final report file using Genome Studio software. Final report files were the converted to PLINK[3] LGEN and MAP format using in-house Perl scripts. **Variant QC**: Duplicate variants were evaluated for missingness and those with the best genotyping rate were retained. Variants with a genotyping rate equal to or greater than 98% and a minor allele frequency (MAF) of 2% or more were retained. **Sample QC**: Samples with a genotyping rate less than 98% or having discordant sex or those with a PLINK heterozygosity estimate (F) beyond three standard deviations (μ(F)±3sd) were excluded. One sample from each pair of related samples (PLINK PI_HAT>0.125), with the best call rate was retained. Population outliers were excluded using Eigenstrat[20,21] which was set to remove outliers of up to 6 standard deviations of the top 10 principal components (PCs) over five iterations, while refitting PCs after each iteration of outlier removal. Imputation Workflow: Prior to imputation, variant strand, alleles and position and alleles were aligned to the HRC reference panel[22] using tools provided by the McCarthy Group (https://www.well.ox.ac.uk/~wrayner/tools/). Genotypes were uploaded to the Michigan Imputation Server[23] and run in "QC only" mode to identify and remove variants with mismatched allele frequencies. Genotypes were then imputed to the HRC (r1.1.2016) reference panel with Eagle (v2.3) phasing[24]. Since imputation replaces genotyped-genotypes with imputed doses, original genotypes were reinserted back into the VCFs using in-house scripts. Dosages were then exported from the VCF using PLINK2. Variants with and imputation R2 ≥ 0.7 and MAF ≥ 2% were retained for downstream analysis. Variants were annotated using ANNOVAR[25].

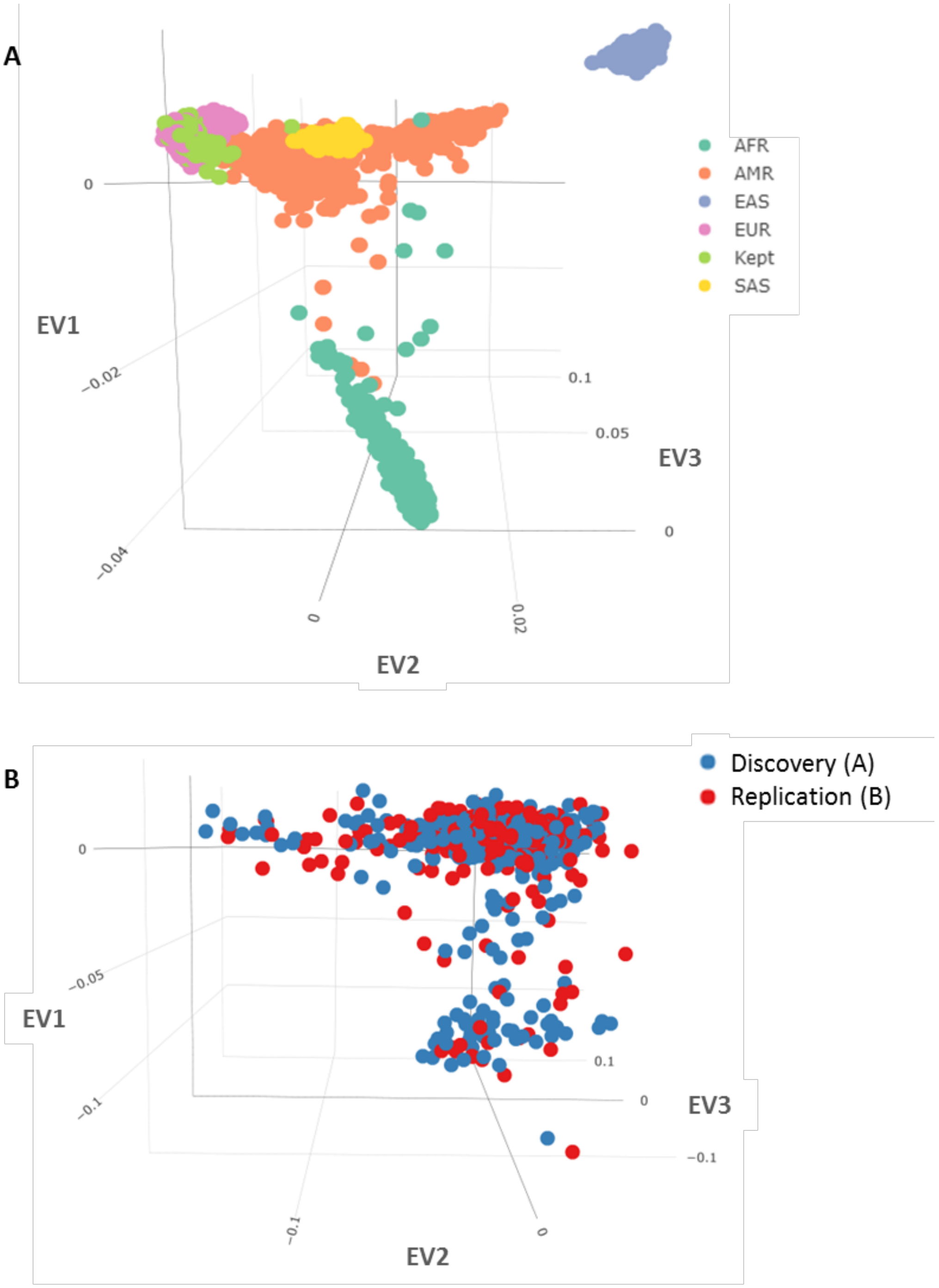

FigureS2: Principal components analysis showing the first three eigenvectors (EV1-3) of samples retained following quality control in the combined cohort (N=821). A. Superimposed with 1000 genomes super populations and **B.** Zoomed in view of study cohort samples, colored by batch.

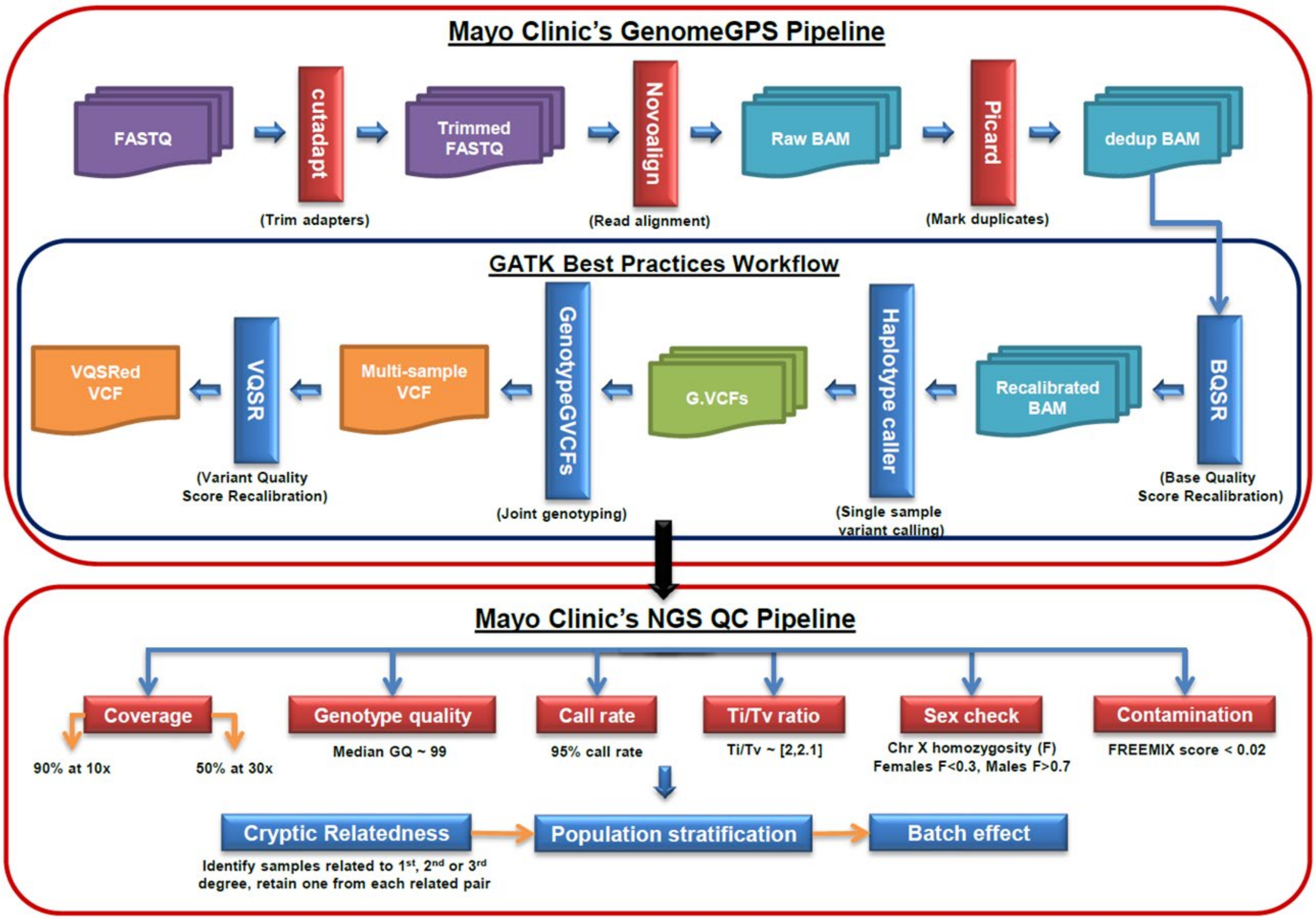

Figure S3. AMP-AD WGS QC. DNA Samples were processed and sequenced as described on the AMP-AD knowledge portal: https://adknowledgeportal.synapse.org/, Synapse ID: **syn10901601**. FastQ files from sequencing were processed through Mayo Clinic's GenomeGPS pipeline. Briefly, reads were aligned to the reference (hg19) using Novoalign and variant calling and genotyping was performed while implementing GATK's Best Practices Workflow[26]. Samples were QC-ed for coverage (at least 90% covered at 10x and 50% covered at 30x), genotyping quality (median GQ of 99), call rate (95%), transition to transversion (Ti/Tv) ratio (between 2 and 2.1), sex (PLINK inbreeding coefficient of the X-chromosome for males>0.7 and females <0.3) and contamination (VerifyBamID[27] FREEMIX score less than 0.02). Subsequently, samples were evaluated for relatedness up to 3rd degree, population substructure and sequencing batch effects. Variants passing VQSR filter, having a genotyping rate of 95% or more, a Bonferroni adjusted Hardy-Weinberg p-value greater than 0.05 in controls and BLAT[28] score less than four were retained for downstream analysis. All 349 samples and 19,357,792 variants passed QC.

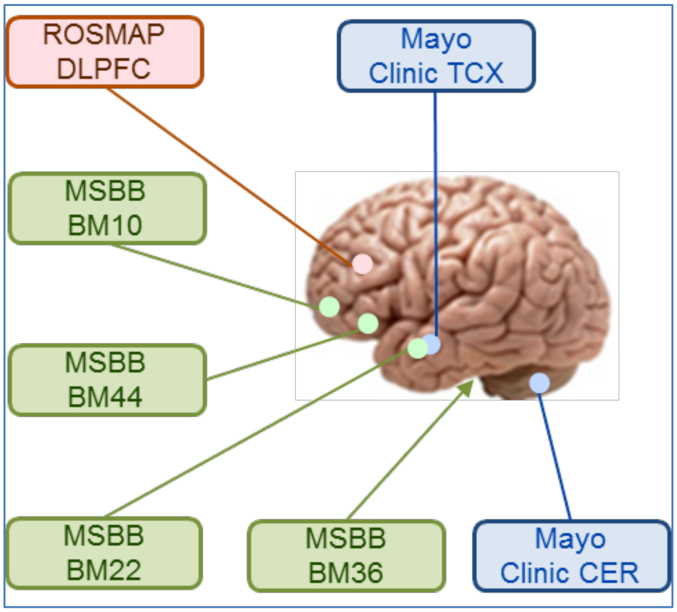

Figure S4. The AMP-AD consortium generated RNAseq brain transcriptome measures from seven brain regions as part of three independent studies. Raw data was reprocessed through a consensus pipeline to reduce between study variability (https://adknowledgeportal.synapse.org/, Synapse ID: **syn17010685**). Rigorous quality control of RNASeq data and associated metadata was performed (**Tables S2 – S3**) and raw counts were CQN normalized. The "expressed transcriptome" was defined based on the distribution of CQN values, with a threshold set for each dataset as follows: Mayo Clinic TCX = 0.5; Mayo Clinic CER = -2; ROSMAP DLPFC = 1.5; MSBB-BM10 = 0.5; MSBB-BM22 = 0.5; MSBB-BM36 = 0.5; MSBB-BM44 = 2.5. ROSMAP = Religious orders study and memory aging project, MSBB = Mount Sinai brain bank; BM = Brodmann Area; DLPFC = dorsolateral prefrontal cortex; TCX = temporal cortex; CER = cerebellum.

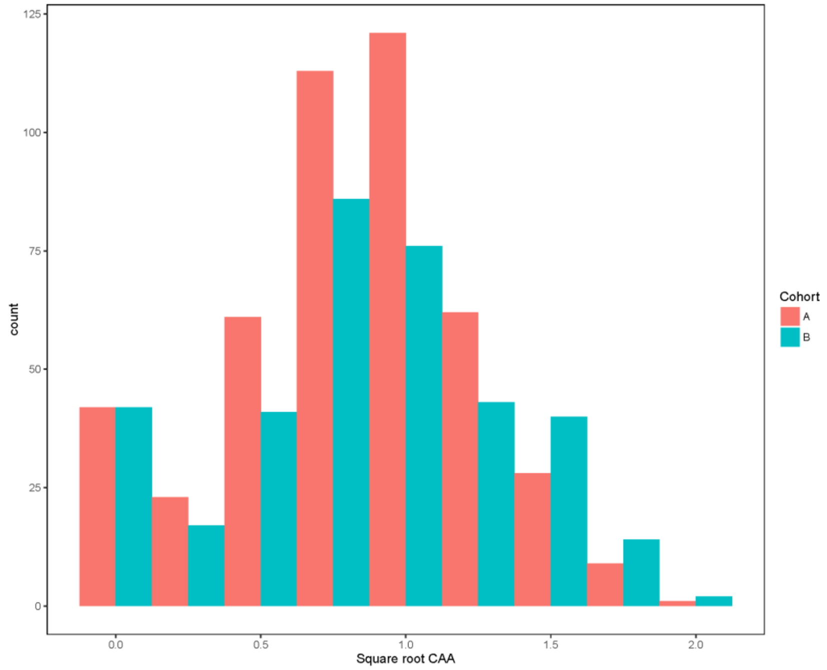

Figure S5: Distribution of square root transformed average CAA scores in two study batches (A and B). The distribution of CAA scores is not significantly different between the two study batches.

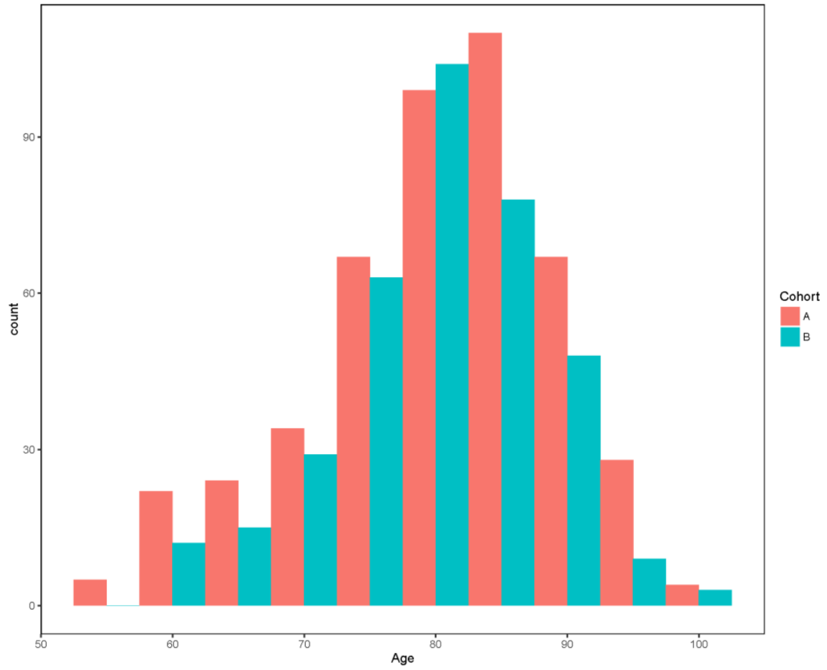

Figure S6: Distribution of age at death in two study batches (A and B). The distribution of age at death is not significantly different between the two study batches.

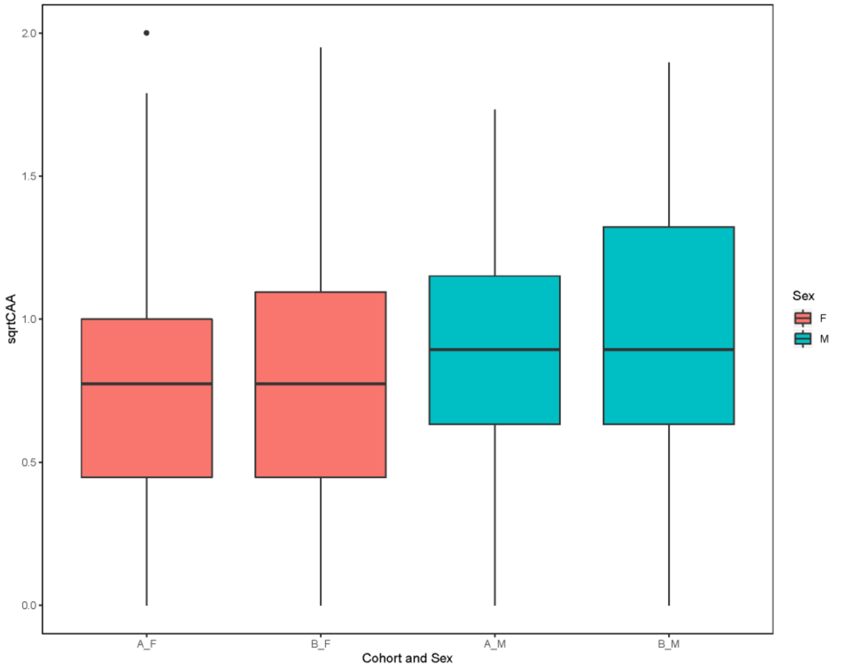

Figure S7: Distribution of square root transformed average CAA scores by Sex in batches A and B (N=821). Higher CAA scores are observed in males than in females across both batches**.**

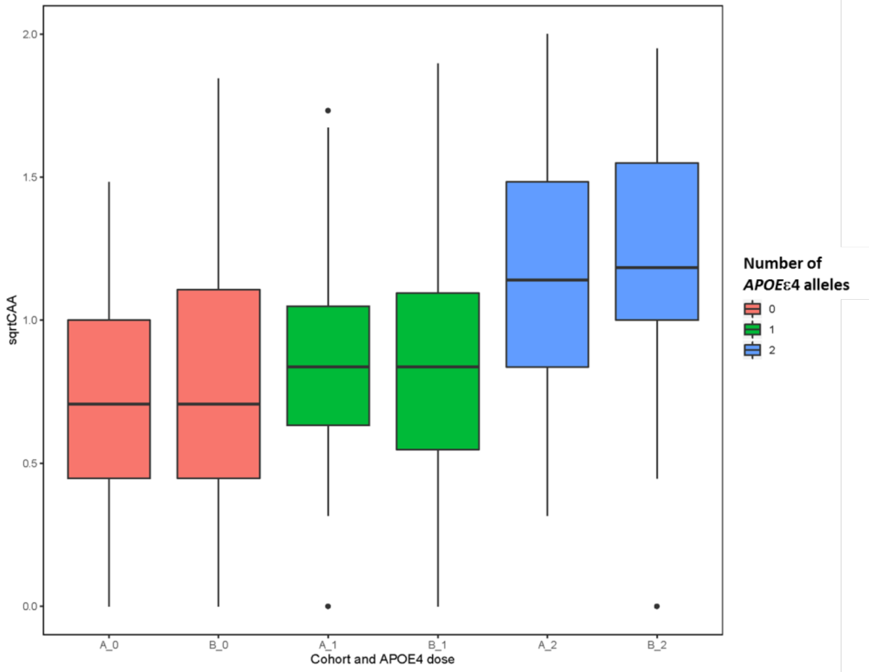

Figure S8: Distribution of square root transformed average CAA scores by *APOE*ε4 dose in batches A and B (N=821). *APOE*ε4 dose is associated with increased CAA score in both study batches.

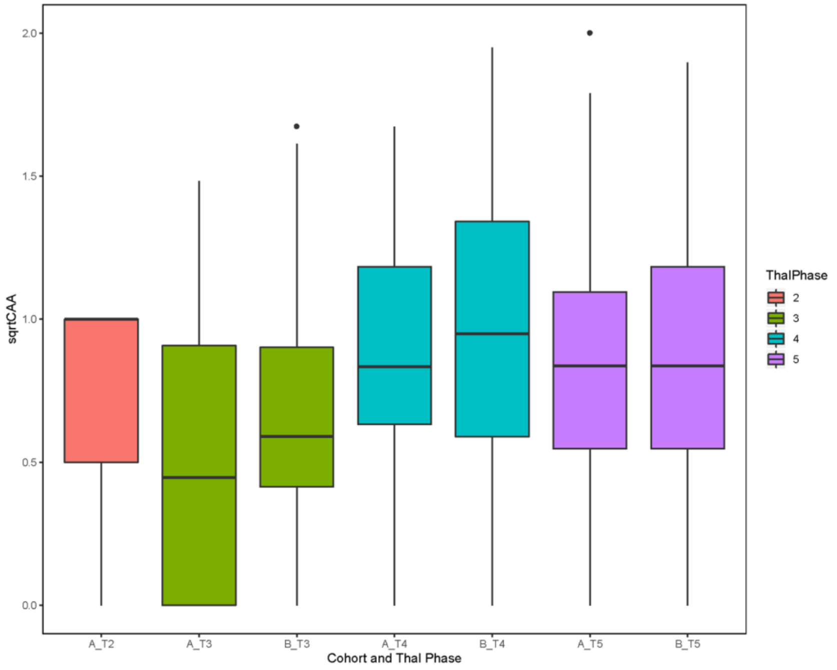

Figure S9: Distribution of square root transformed average CAA scores by Thal phase in batches A and B (N=821). Samples with a Thal phase of 2 or 3 were grouped as "Thal Medium", while Thal phase of 4 or 5 were grouped as "Thal High". Thal phase "high" trends with increased CAA score in both study batches. Note that Thal phase was grouped due to there being no significant difference in sqrtCAA between Thal phase 4 and 5.

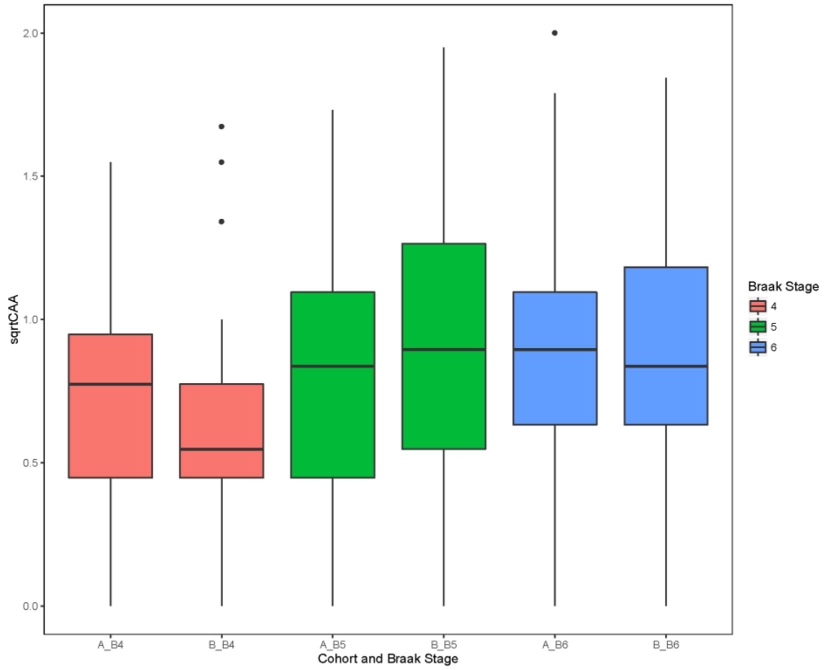

Figure S10: Distribution of square root transformed average CAA scores by Braak score in batches A and B (N=821). A trend for higher CAA score with increasing Braak score is observed across both study batches.

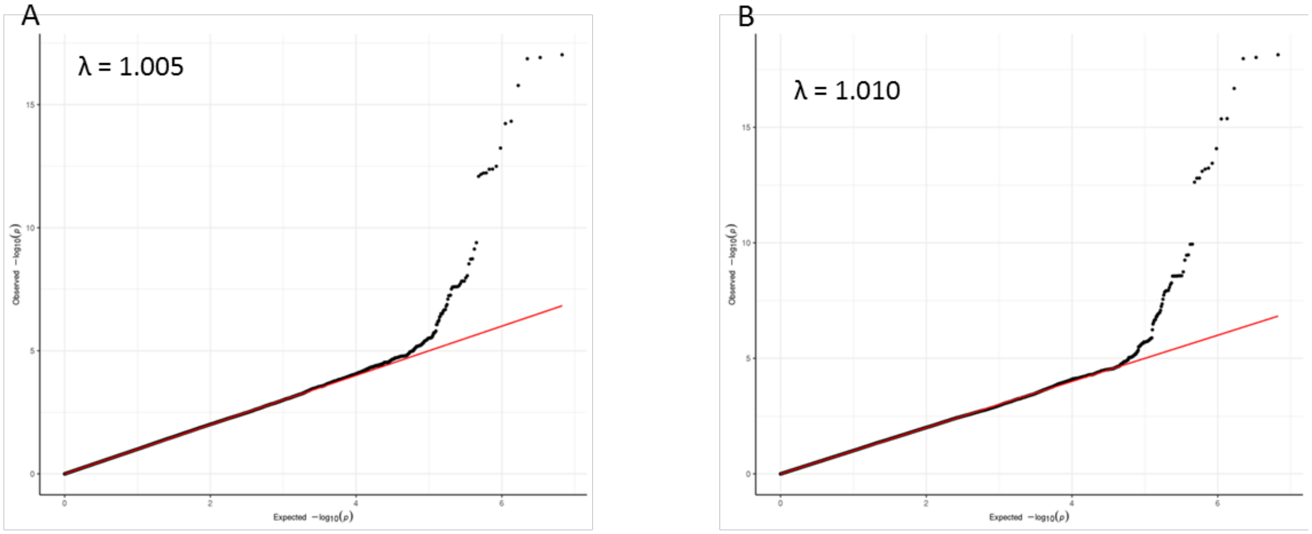

Figure S11: Q-Q plots for combined cohort p-values following imputation for (A) CAA with Braak and Thal adjustment; (B) CAA without Braak and Thal adjustment. The genomic inflation factor (lambda) indicates that there is no evidence of inflation of the test statistic.

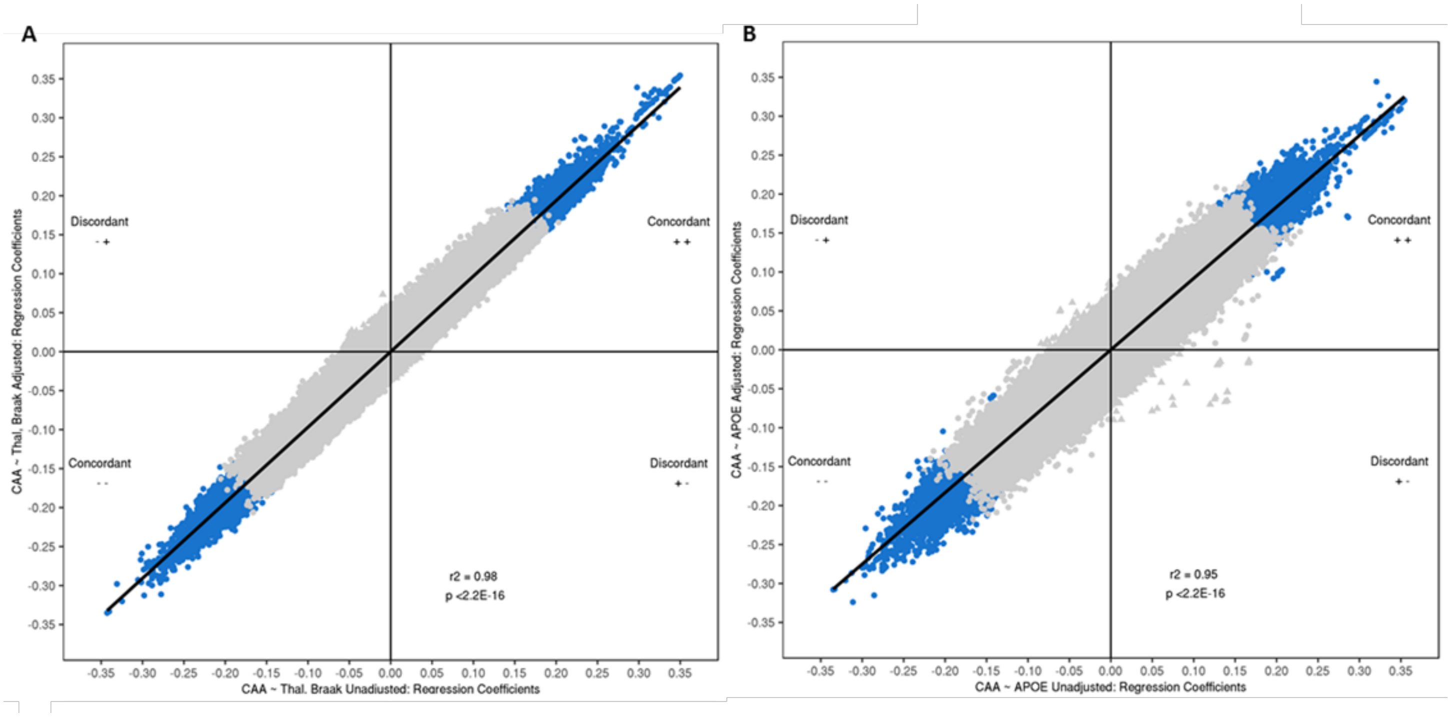

Figure S12: CAA GWAS results under various models tested are highly similar. Correlation plots comparing beta values for regression models **a**. with and without adjustment for AD neuropathology, and **b**. with AD neuropathology, with and without adjustment for *APOE*ε2 and *APOE*ε4 alleles. Each colored point represents one SNP positioned based on the regression beta values for association with CAA under the two indicated models. SNPs with a p-value < 0.05 in both models are colored in blue; all others are colored in grey. Circles represent SNPs with concordant beta values under both models; triangles represent SNPs with discordant beta values between the two models. The spearman rank correlation coefficient (r2) and p-value for the two sets of beta values are indicated on the plot.

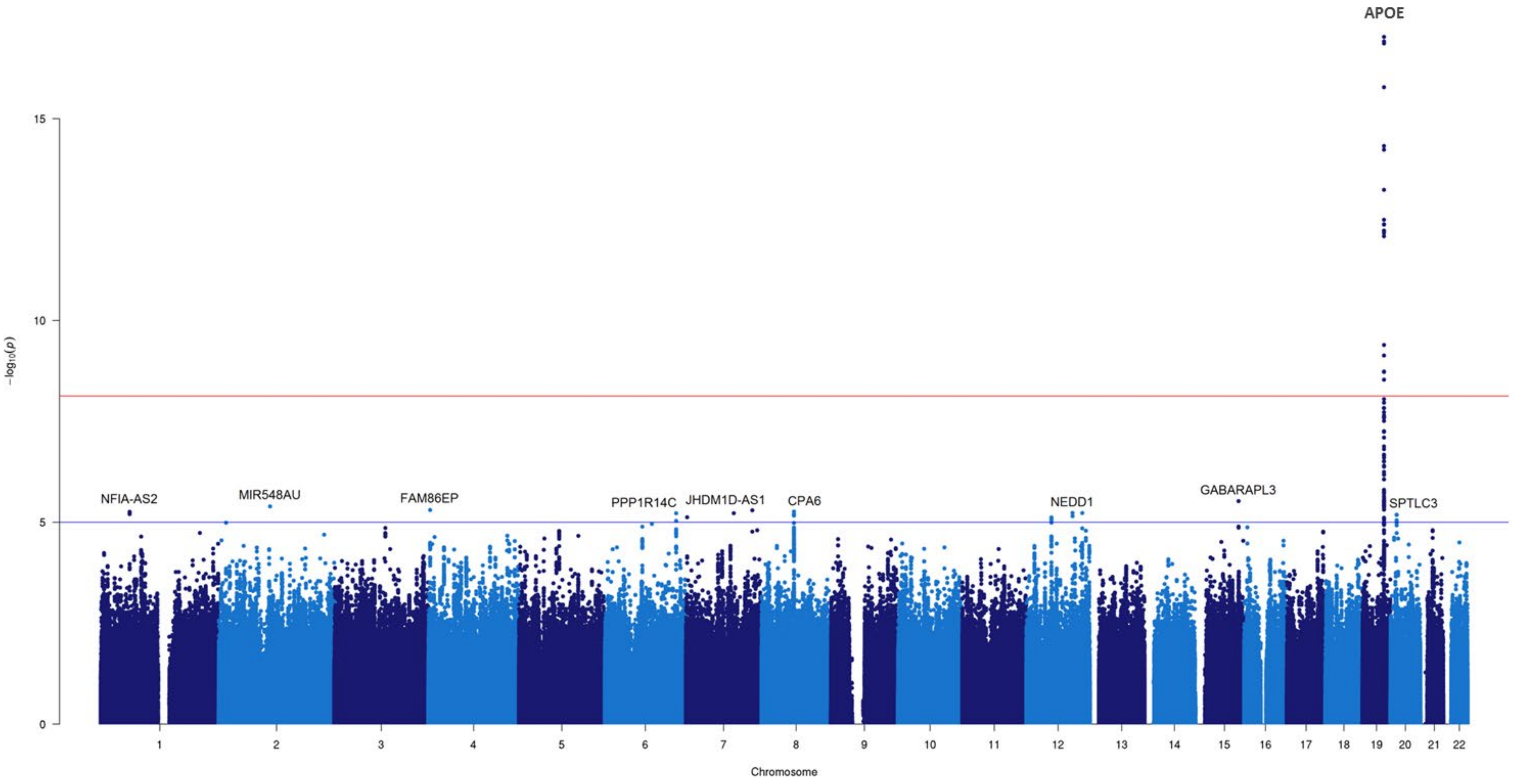

Figure S13: Manhattan plot representing results of genome-wide association results for CAA. Results are shown for the primary model where square root adjusted average CAA scores were tested for association with genome-wide variants adjusting for Age, Sex, batch, PC1-3, Thal phase and Braak stage.

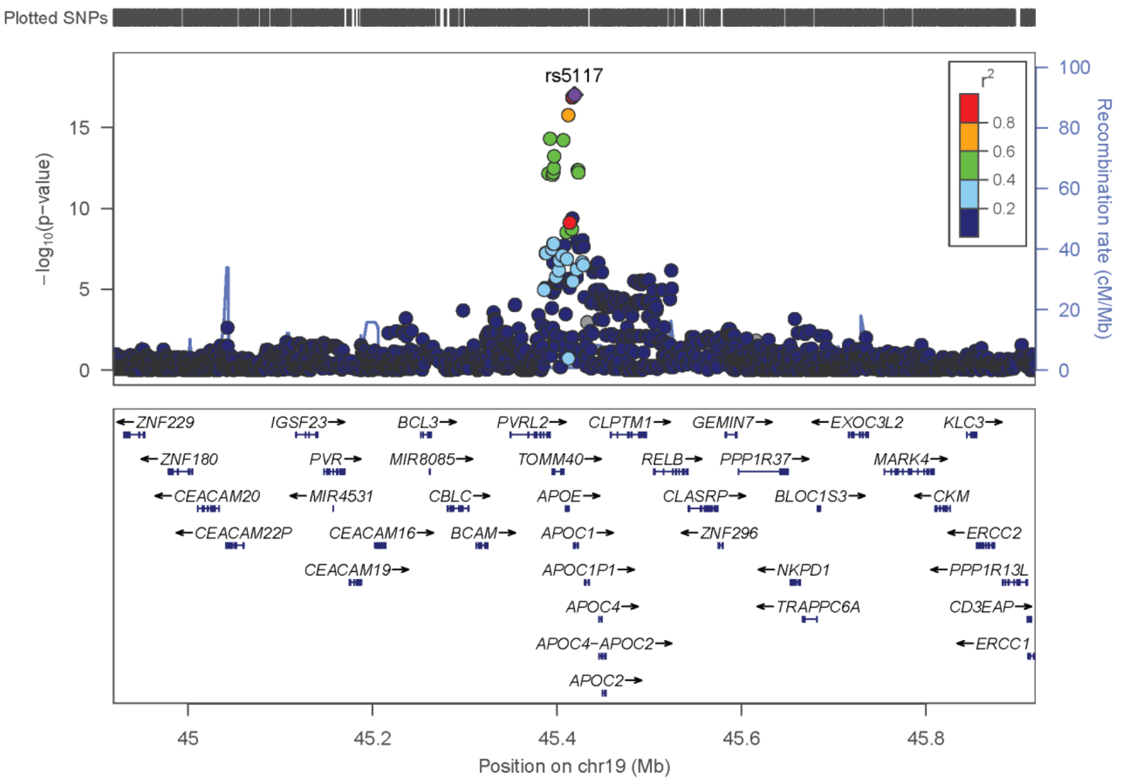

Figure S14: *APOE* is the most significant locus associated with CAA pathology in AD cases. Locus Zoom plot showing association of variants at the *APOE* locus with CAA. The most significant variant (rs5117, Chr19: 45418790) is indicated in purple, with 500kb flanking region 5' and 3' of this variant included in the plot. The association p-value is shown on the Y-axis and linear position on the chromosome on the X axis. Each point on the plot represents one variant; the colors of the points indicate the linkage disequilibrium (r2) value with the index variant (rs5117).

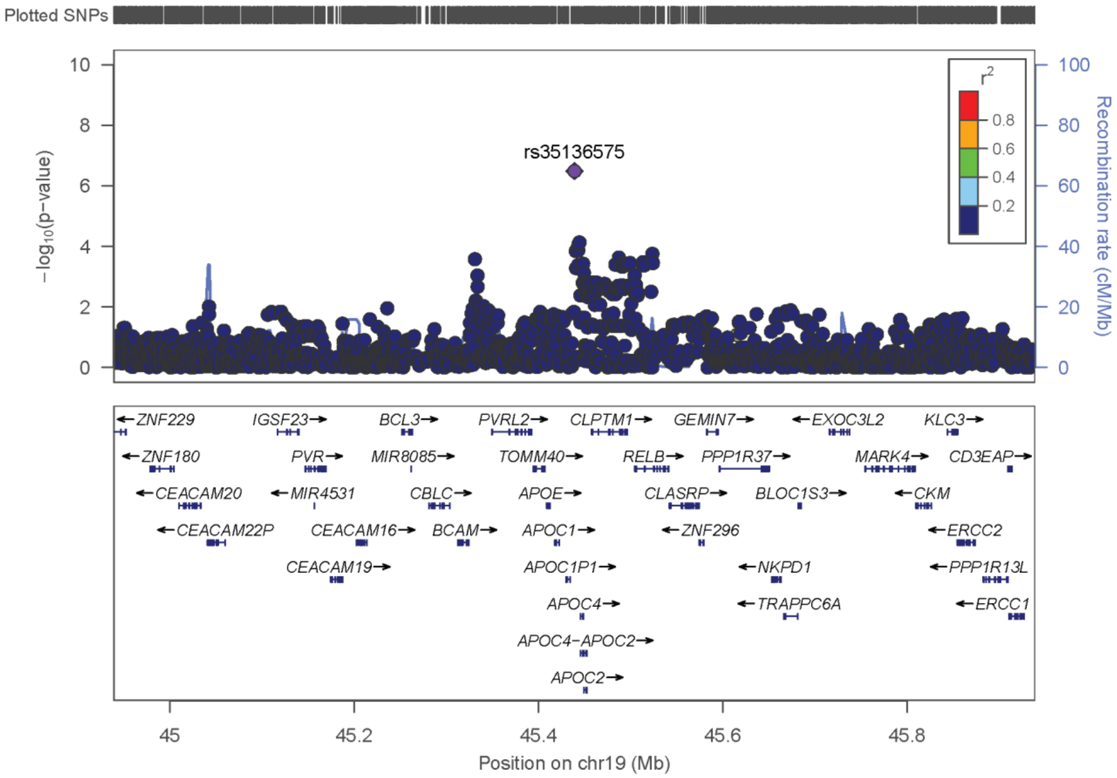

Figure S15: A variant at the *APOE* locus is the most significant variant associated with increased CAA pathology after controlling for *APOE*ε2 and ε4 alleles. Locus zoom plot showing results for association of variants with CAA in the region flanking rs35136575, adjusting for Braak, Thal, and *APOE*ε2 and ε4 alleles. See Figure S15 for additional details.

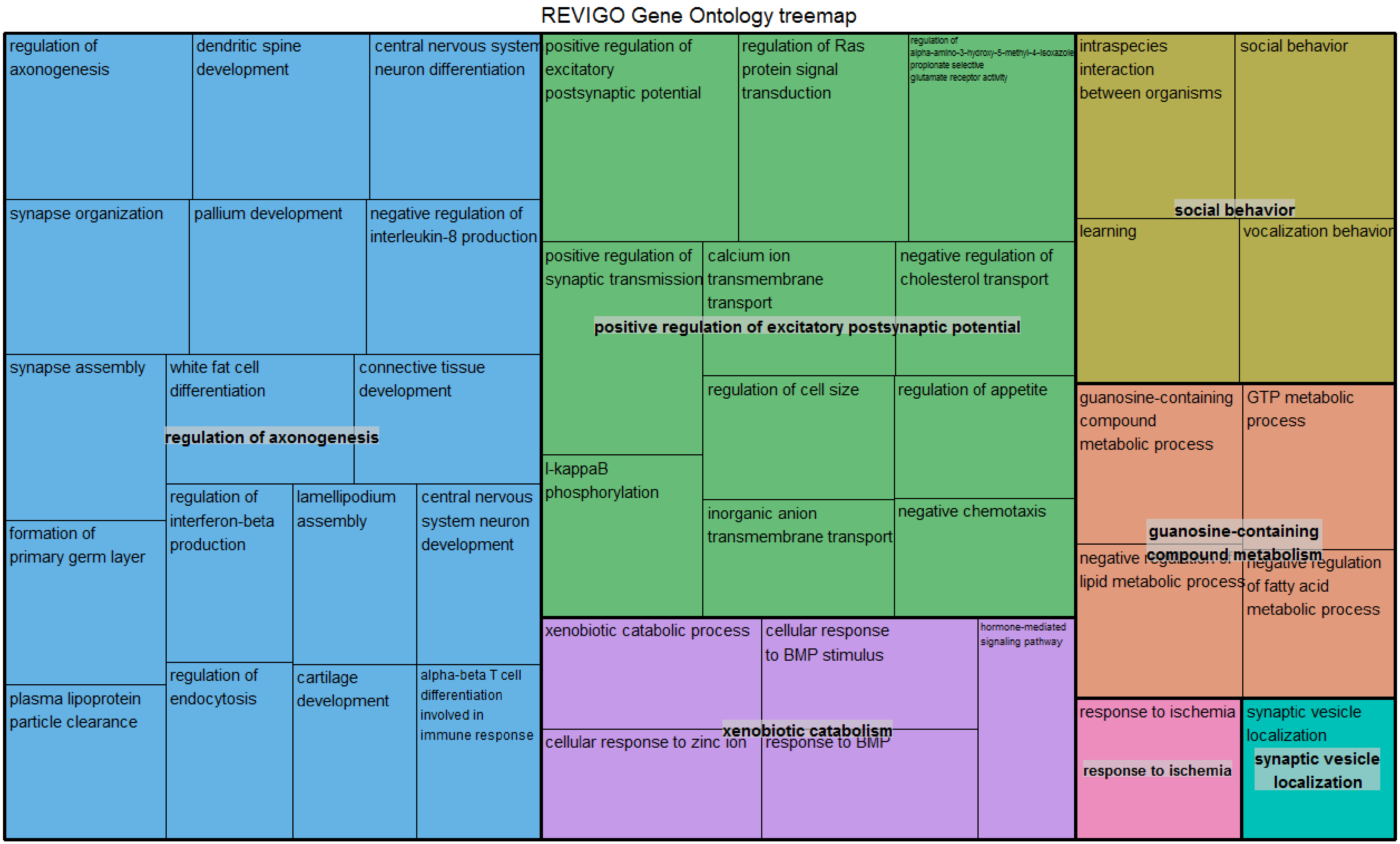

Figure S16: Gene Ontology Biological processes enriched for genes associated with CAA in AD cases under the primary model. REVIGO[15] was used to organize GO-BP, and generate treemap plots summarizing GO-BP, with an FDR q-value < 0.05. GO-BP are organized based on similarity, with similar boxes sharing color and located proximal, box size reflects GO-BP pvalue; the most significant GO-BP in each plot is named in the central area of each group of similar terms.

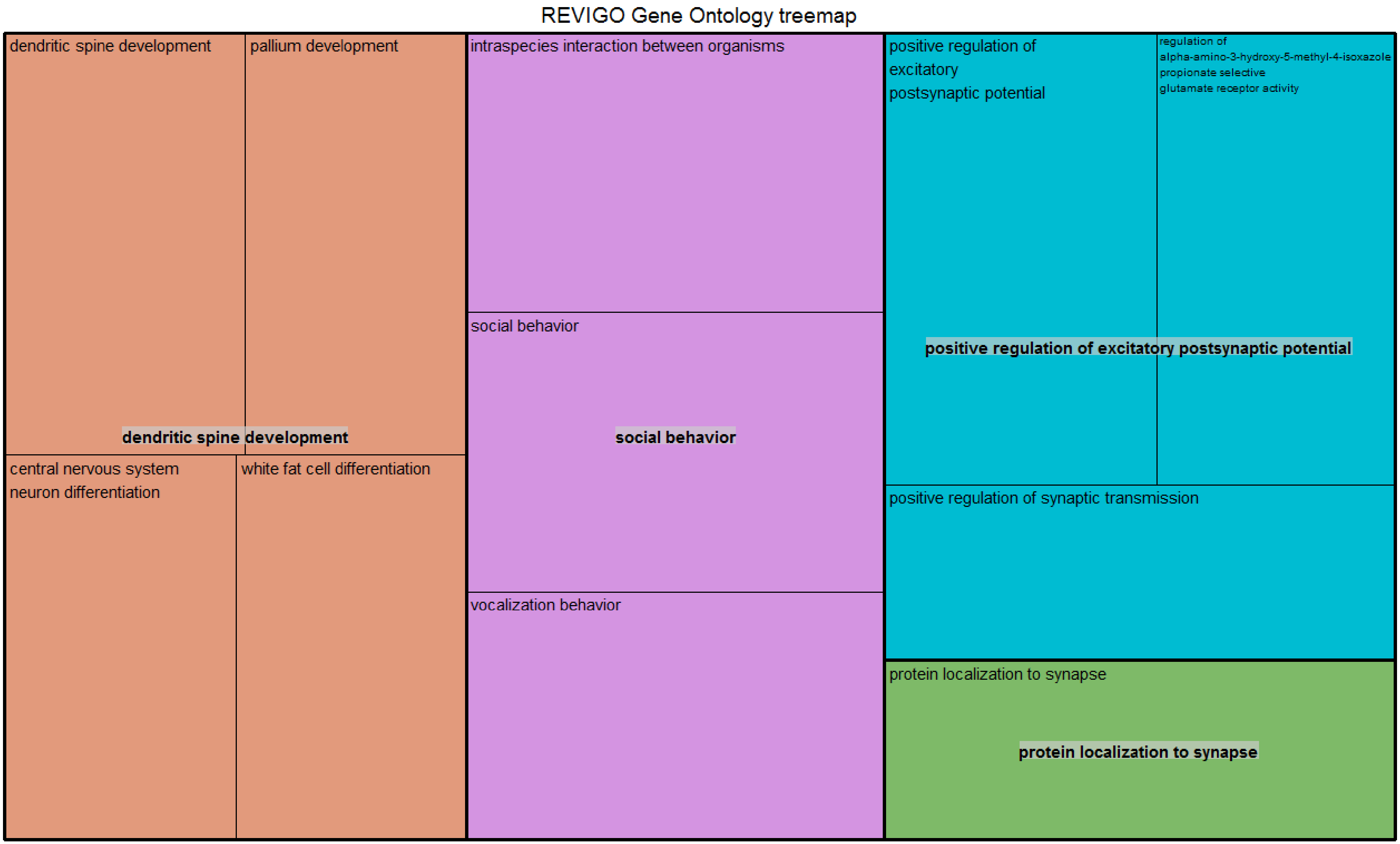

Figure S17. Gene Ontology Biological processes enriched for genes associated with CAA in AD cases after adjustment for APOEε2 and APOEε4 alleles. See Figure S16 for legend.

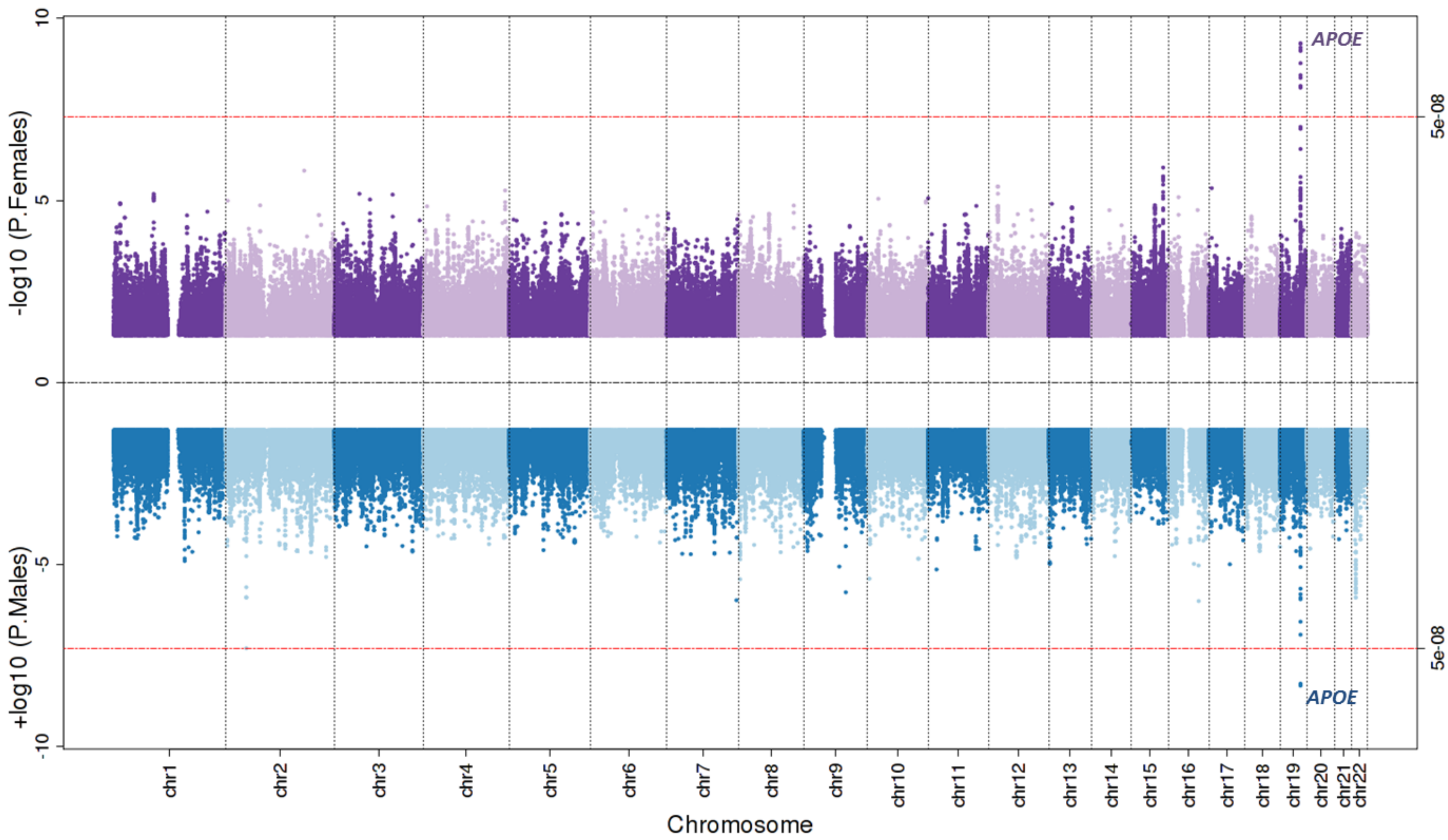

Figure S18: Miami plot illustrating results of genome-wide association analysis for square root CAA conducted in Female (upper panel) and Male (lower panel) participants, separately.

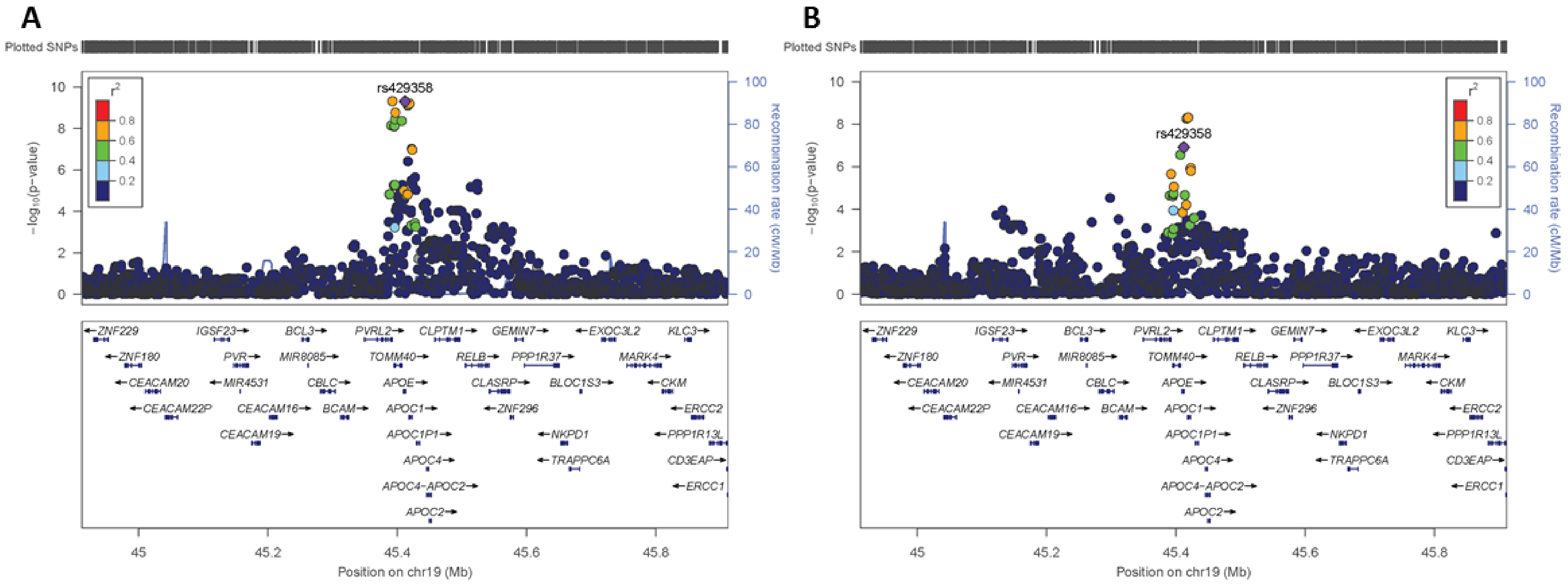

Figure S19: Locus Zoom plots of CAA association with SNPs at the *APOE* locus in females (A) and males (B) separately. The *APOE*ε4 tagging variant, rs429358, is indicated as the purple point in each plot; the colors of the other points indicate the linkage disequilibrium (r2) value with this variant (rs429358). See figure S15 for additional legend details.

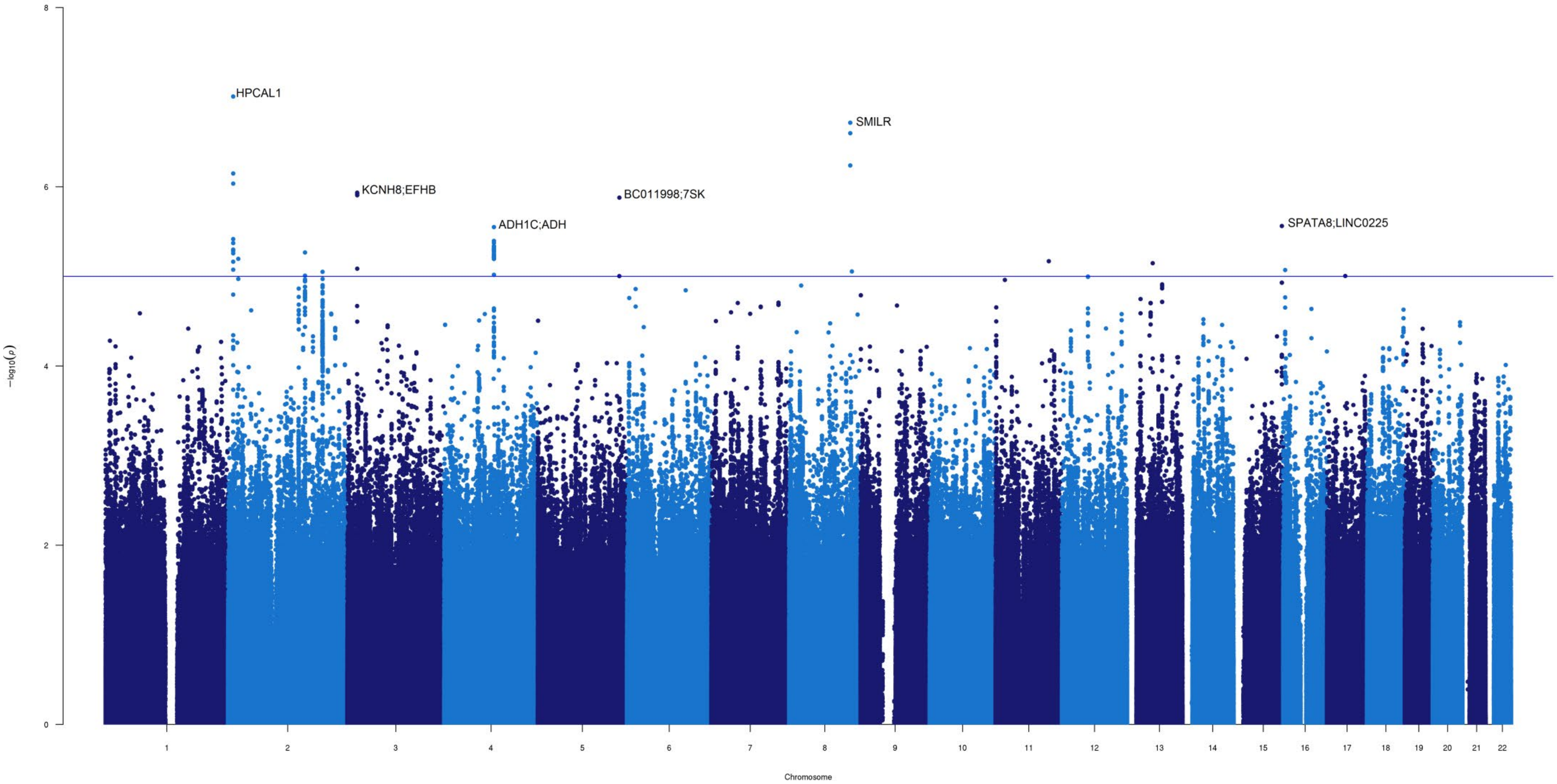

Figure S20: Manhattan plot representing results of genome-wide CAA sex-interaction analysis. Small p-values indicate SNP has sex-divergent association with CAA.

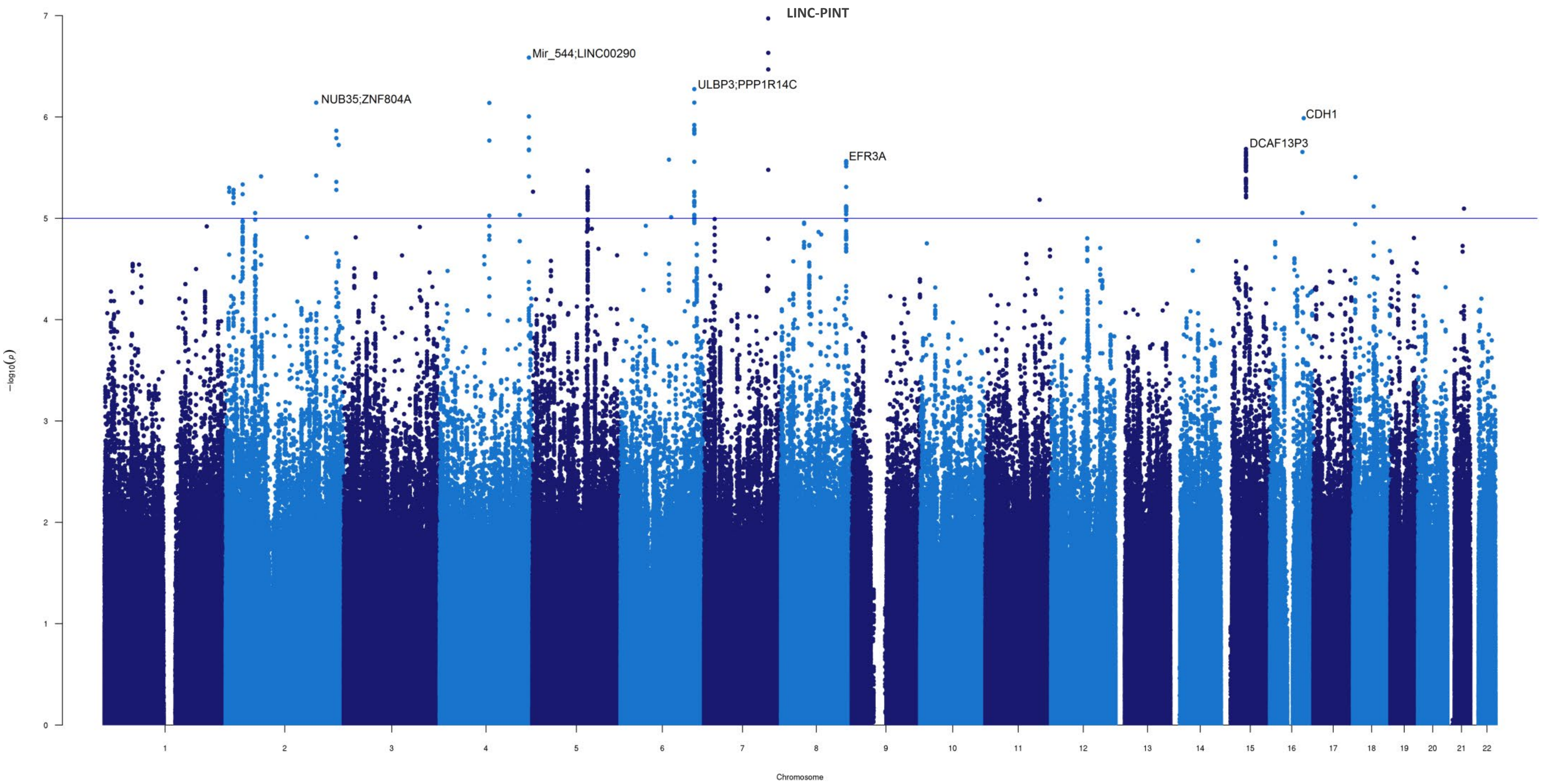

Figure S21: Manhattan plot representing results of genome-wide CAA *APOE*ε4 status (+/-) interaction analysis. Small p-values indicate SNP has *APOE*ε4-divergent association with CAA. *LINC-PINT* locus has the most significant *APOE* interaction for CAA association.

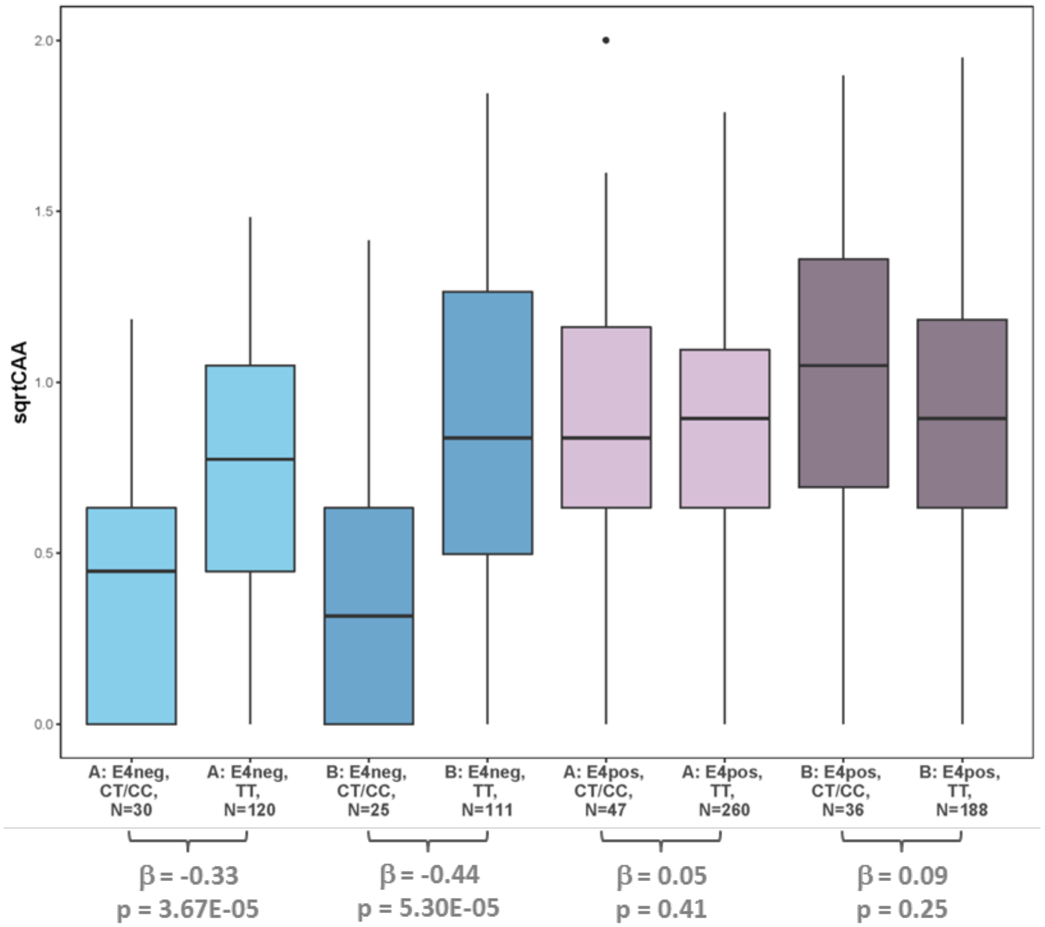

Figure S22: Distribution of square root transformed average CAA scores by *APOE*ε4 status and *LINC-PINT* rs10234094 minor allele (C) carrier status, in two genotyping batches A and B (N=817). In the *APOE*ε4 negative subset (E4neg), CAA scores are significantly *lower* in rs10234094 minor allele carriers (CT/CC) than in TT homozygotes. In the *APOE*ε4 positive subset (E4pos), CAA scores are not significantly different between rs10234094 minor allele carriers (CT/CC) and TT homozygotes. These differences are consistent across both genotyping batches (A and B). Prior to plotting, imputed gene dosages were converted to "hard calls"; four individuals were excluded due to dosages outside of the defined ranges (Methods), sample size per group is indicated. P-values were obtained from a dominant model (CT+CC), adjusting for Age, Sex, Braak stage, Thal phase and 3 population principal components.

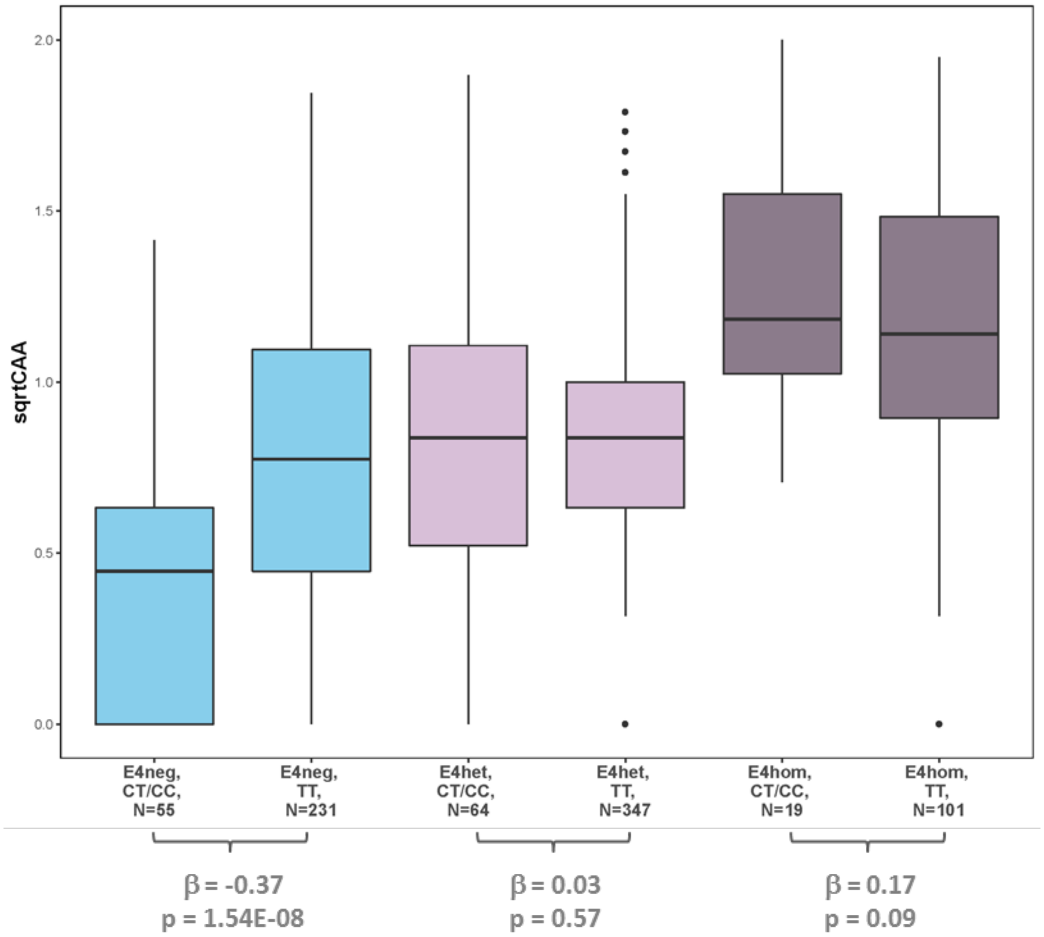

Figure S23: Distribution of square root transformed average CAA scores by *APOE*ε4 dose and *LINC-PINT* rs10234094 minor allele (C) carrier status (N=817). In the *APOE*ε4 negative subset (E4neg), CAA scores are significantly *lower* in rs10234094 minor allele carriers (CT/CC) than in TT homozygotes. In the *APOE*ε4 heterozygote (E4het), and *APOE*ε4 homozygote (E4hom) subsets CAA scores are not significantly different between rs10234094 minor allele carriers (CT/CC) and TT homozygotes. Prior to plotting, imputed gene dosages were converted to "hard calls"; four individuals were excluded due to dosages outside of the defined ranges (Methods), sample size per group is indicated. P-values were obtained from a dominant model (CT+CC), adjusting for genotyping batch, Age, Sex, Braak stage, Thal phase and 3 population principal components.

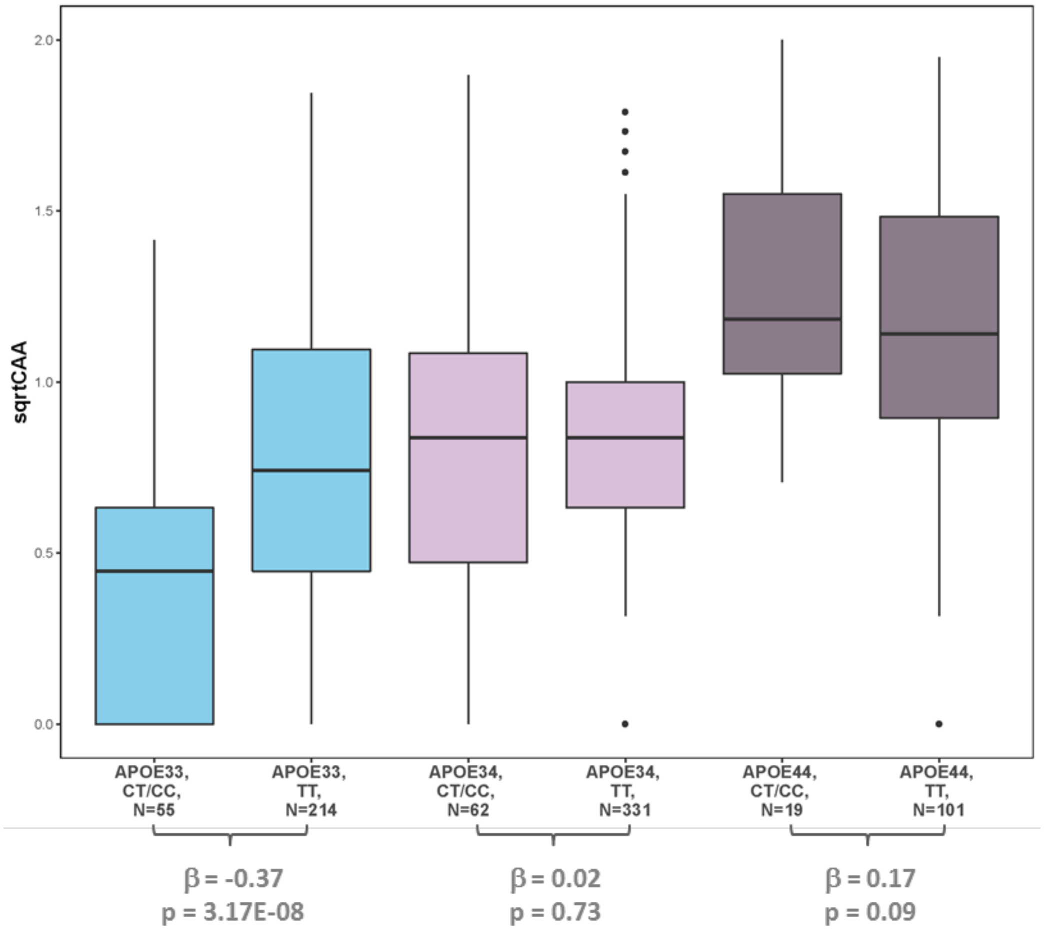

Figure S24: Distribution of square root transformed average CAA scores by *APOE* haplotype and *LINC-PINT* rs10234094 minor allele (C) carrier status (N=782). In the *APOE*ε3 homozygotes, CAA scores are significantly *lower* in rs10234094 minor allele carriers (CT/CC) than in TT homozygotes. In the *APOE*ε34 group, and *APOE*ε4 homozygote subsets CAA scores are not significantly different between rs10234094 minor allele carriers (CT/CC) and TT homozygotes. Prior to plotting, imputed gene dosages were converted to “hard calls”; four individuals were excluded due to dosages outside of the defined ranges (Methods), and individuals with *APOE*ε23 or *APOE*ε24 genotypes were removed. Sample size per group is indicated. P-values were obtained from a dominant model (CT+CC), adjusting for genotyping batch, Age, Sex, Braak stage, Thal phase and 3 population principal components.

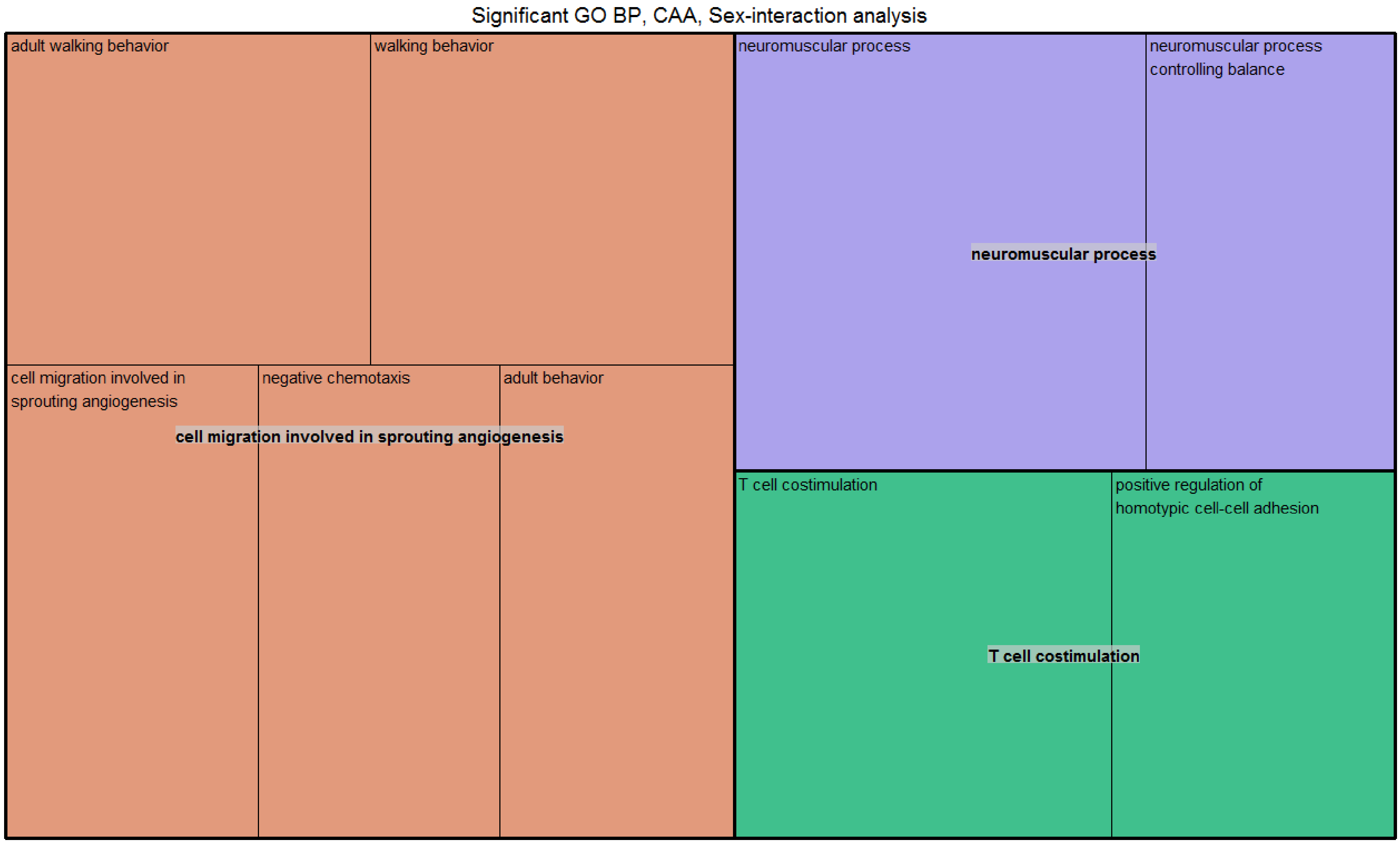

Figure S25: Gene Ontology Biological processes enriched for genes associated with CAA in the sex-interaction analysis. See Figure S16 for legend.

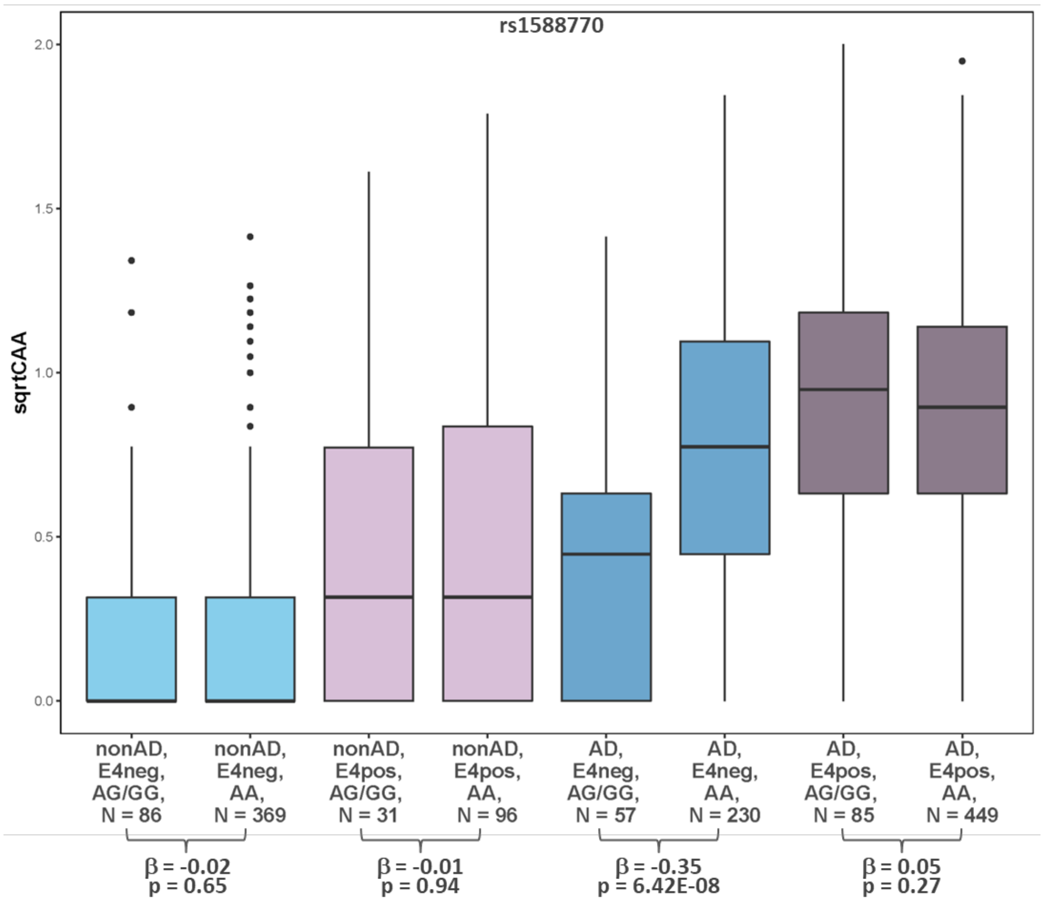

Figure S26: Distribution of square root transformed average CAA scores by diagnosis (AD/nAD), *APOE*e4 status (+/-) and *LINC-PINT* rs1588770 minor allele (G) carrier status (N=1,403). Sample size per group is indicated. P-values were obtained from a dominant model (CT+CC), adjusting for genotyping batch, Age, Sex, Braak stage, Thal phase, and 3 population principal components (AD's only).

A

Sample	Diagnosis	Sex	AgeAtDeath	RIN	APOE	RNAseq Expression	RNAscope Batch	Hscore
S1	AD	F	82	8.7	33	Low	1	0.167
S2	AD	M	75	8.7	34	Low	1	0.240
S3	AD	M	82	7.2	33	High	1	0.425
S4	AD	F	83	7.5	34	High	1	0.421
S5	AD	F	77	9.7	33	Low	2	0.427
S6	AD	F	90+	8.4	34	Low	2	0.423
S7	AD	F	85	8	33	High	2	0.539
S8	AD	F	76	8.5	34	High	2	0.678

C

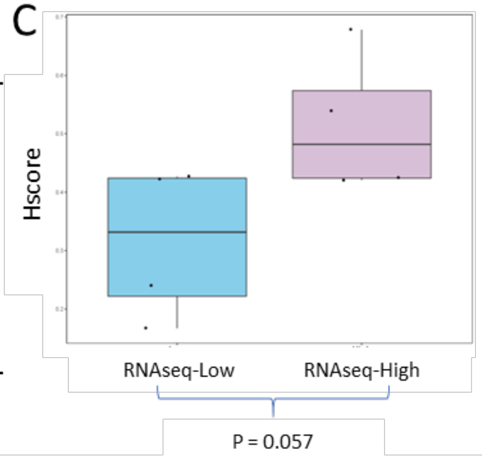

B

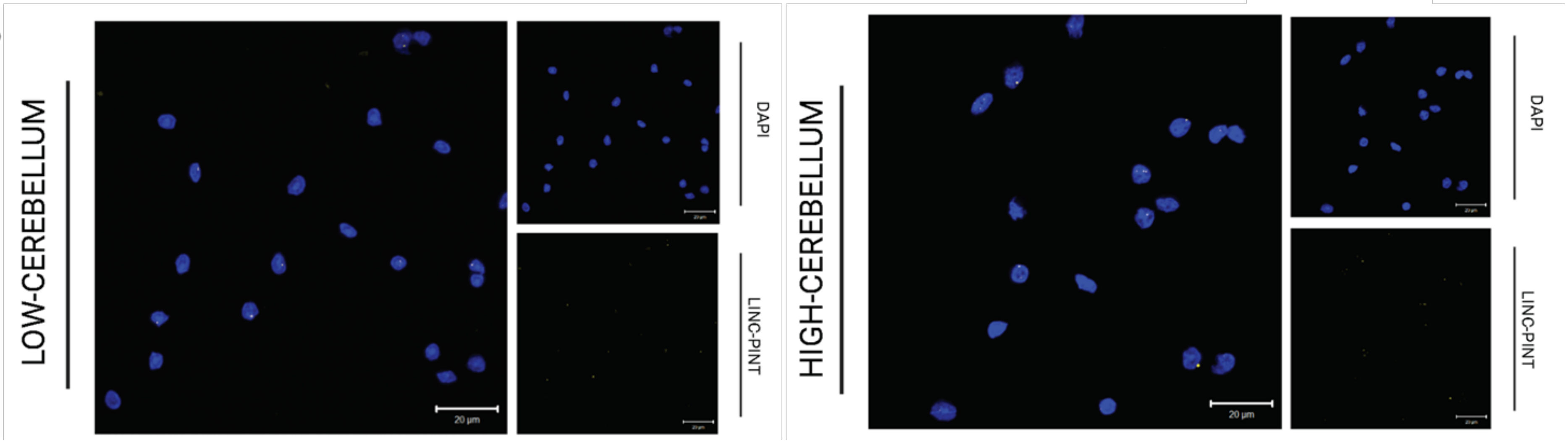

Figure S27: Visualization of *LINC-PINT* expression through *in situ* hybridization. A) Eight cerebellum tissue of AD patients that show high and low *LINC-PINT* expression in RNAseq study were selected. B) LINC-PINT probe (ACDBiotech - 477631) overlaid with DAPI. Five images per sample were captured and H-score calculated. C) The difference in H-score between high and low expressing nuclei were analyzed via Mann-Whitney test; high cerebellar nuclei confirmed the trend for increased *LINC-PINT* expression compared to low cerebellar samples (n=8).

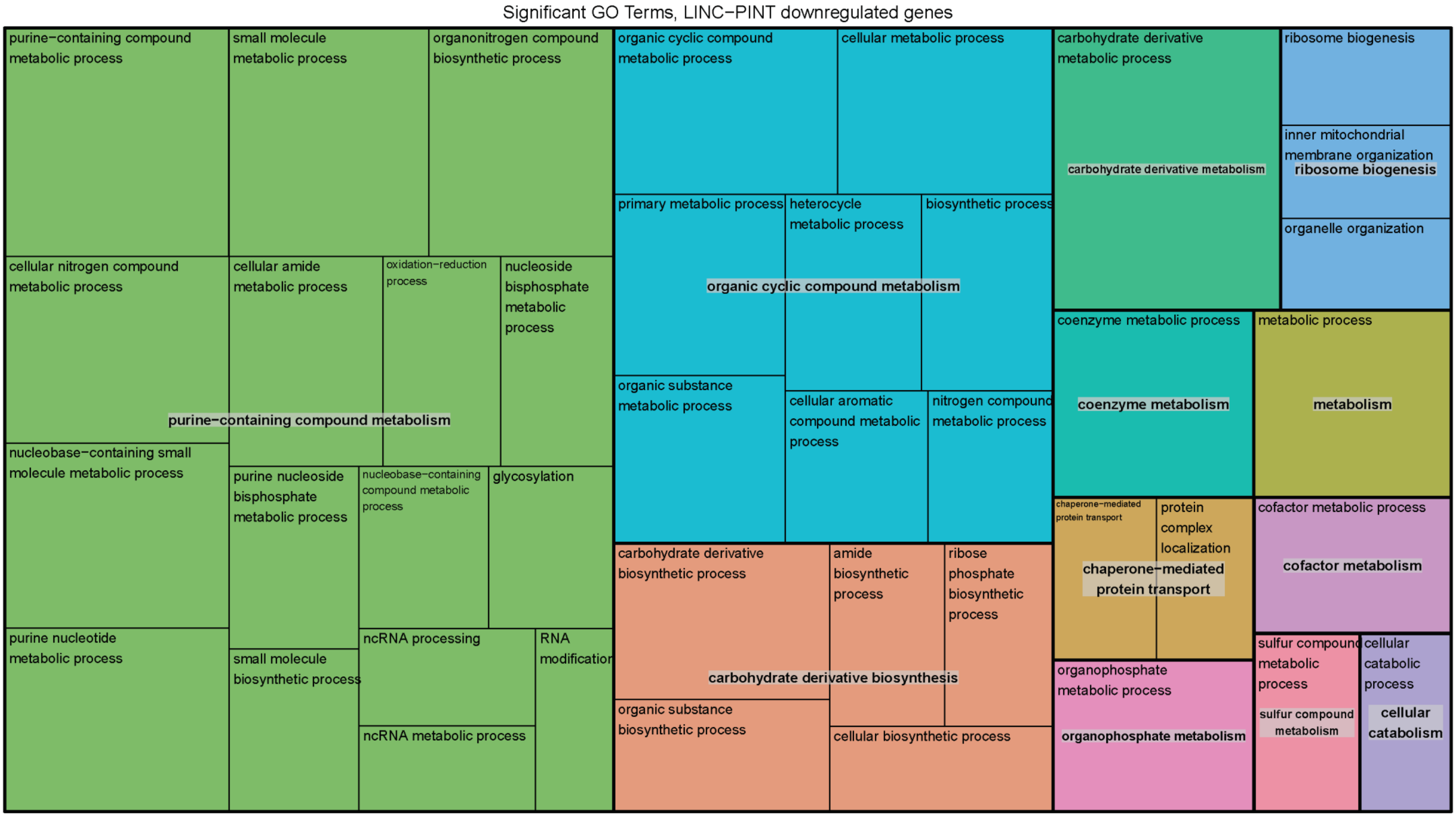

Figure S28: Gene Ontology Biological processes enriched for genes inversely associated with *LINC-PINT* brain gene expression. See Figure S16 for legend.

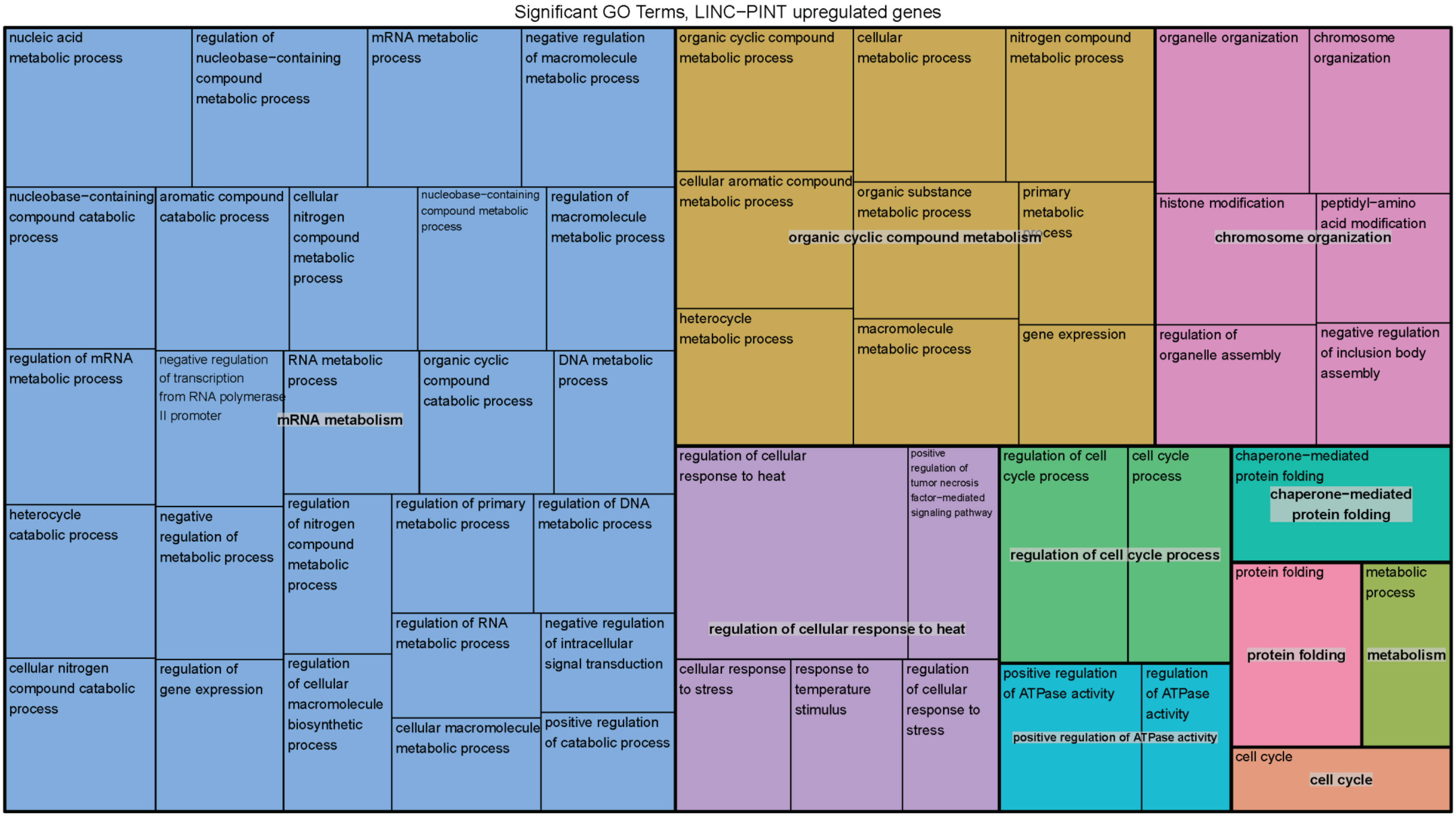

Figure S29: Gene Ontology Biological processes enriched for genes positively associated with *LINC-PINT* brain gene expression. See Figure S16 for legend.

References cited in the supplement

1. Murray, M.E. *et al.* Neuropathologically defined subtypes of Alzheimer's disease with distinct clinical characteristics: a retrospective study. *Lancet Neurol* **10**, 785-96 (2011).
2. Murray, M.E. *et al.* Clinicopathologic and 11C-Pittsburgh compound B implications of Thal amyloid phase across the Alzheimer's disease spectrum. *Brain* **138**, 1370-81 (2015).
3. Chang, C.C. *et al.* Second-generation PLINK: rising to the challenge of larger and richer datasets. *Gigascience* **4**, 7 (2015).
4. Purcell, S. *et al.* PLINK: a tool set for whole-genome association and population-based linkage analyses. *Am J Hum Genet* **81**, 559-75 (2007).
5. Allen, M. *et al.* Human whole genome genotype and transcriptome data for Alzheimer's and other neurodegenerative diseases. *Sci Data* **3**, 160089 (2016).
6. Allen, M. *et al.* Conserved brain myelination networks are altered in Alzheimer's and other neurodegenerative diseases. *Alzheimers Dement* **14**, 352-366 (2018).
7. Hansen, K.D., Irizarry, R.A. & Wu, Z. Removing technical variability in RNA-seq data using conditional quantile normalization. *Biostatistics* **13**, 204-16 (2012).
8. Boyle, A.P. *et al.* Annotation of functional variation in personal genomes using RegulomeDB. *Genome Res* **22**, 1790-7 (2012).
9. Rentzsch, P., Witten, D., Cooper, G.M., Shendure, J. & Kircher, M. CADD: predicting the deleteriousness of variants throughout the human genome. *Nucleic Acids Res* **47**, D886-D894 (2019).
10. Logsdon, B. *et al.* Meta-analysis of the human brain transcriptome identifies heterogeneity across human AD coexpression modules robust to sample collection and methodological approach. *bioRxiv* **[Preprint]**, https://doi.org/10.1101/510420 (2019).
11. Bates, D., Machler, M., Bolker, B.M. & Walker, S.C. Fitting Linear Mixed-Effects Models Using lme4. *Journal of Statistical Software* **67**, 1-48 (2015).
12. Kenward, M.G. & Roger, J.H. Small sample inference for fixed effects from restricted maximum likelihood. *Biometrics* **53**, 983-97 (1997).
13. Kuznetsova, A., Brockhoff, P.B. & Christensen, R.H.B. lmerTest Package: Tests in Linear Mixed Effects Models. *Journal of Statistical Software* **82**, 1-26 (2017).
14. Zhang, Y. *et al.* Purification and Characterization of Progenitor and Mature Human Astrocytes Reveals Transcriptional and Functional Differences with Mouse. *Neuron* **89**, 37-53 (2016).
15. Supek, F., Bosnjak, M., Skunca, N. & Smuc, T. REVIGO summarizes and visualizes long lists of gene ontology terms. *PLoS One* **6**, e21800 (2011).
16. McKhann, G. *et al.* Clinical diagnosis of Alzheimer's disease: report of the NINCDS-ADRDA Work Group under the auspices of Department of Health and Human Services Task Force on Alzheimer's Disease. *Neurology* **34**, 939-44 (1984).
17. De Jager, P.L. *et al.* A multi-omic atlas of the human frontal cortex for aging and Alzheimer's disease research. *Sci Data* **5**, 180142 (2018).
18. Wang, M. *et al.* The Mount Sinai cohort of large-scale genomic, transcriptomic and proteomic data in Alzheimer's disease. *Sci Data* **5**, 180185 (2018).
19. Mirra, S.S. *et al.* The Consortium to Establish a Registry for Alzheimer's Disease (CERAD). Part II. Standardization of the neuropathologic assessment of Alzheimer's disease. *Neurology* **41**, 479-86 (1991).
20. Patterson, N., Price, A.L. & Reich, D. Population structure and eigenanalysis. *PLoS Genet* **2**, e190 (2006).
21. Price, A.L. *et al.* Principal components analysis corrects for stratification in genome-wide association studies. *Nat Genet* **38**, 904-9 (2006).
22. McCarthy, S. *et al.* A reference panel of 64,976 haplotypes for genotype imputation. *Nat Genet* **48**, 1279-83 (2016).
23. Das, S. *et al.* Next-generation genotype imputation service and methods. *Nat Genet* **48**, 1284-1287 (2016).
24. Loh, P.R. *et al.* Reference-based phasing using the Haplotype Reference Consortium panel. *Nat Genet* **48**, 1443-1448 (2016).

25. Wang, K., Li, M. & Hakonarson, H. ANNOVAR: functional annotation of genetic variants from high-throughput sequencing data. *Nucleic Acids Res* **38**, e164 (2010).
26. Van der Auwera, G.A. *et al.* From FastQ data to high confidence variant calls: the Genome Analysis Toolkit best practices pipeline. *Curr Protoc Bioinformatics* **43**, 11 10 1-11 10 33 (2013).
27. Jun, G. *et al.* Detecting and estimating contamination of human DNA samples in sequencing and array-based genotype data. *Am J Hum Genet* **91**, 839-48 (2012).
28. Kent, W.J. BLAT--the BLAST-like alignment tool. *Genome Res* **12**, 656-64 (2002).
